# Benchmarking Fourier Transform Ion Cyclotron Resonance Mass Spectrometry against Conventional Methods for the Characterization of Solvent-Fractionated Kraft Lignin

**DOI:** 10.1021/acs.biomac.6c01199

**Published:** 2026-07-28

**Authors:** Wiebke Hadwich, Klara Sander, Erica Brendler, Martina Bremer, Steffen Fischer, Carla Vogt, Jan Zuber

**Affiliations:** † Institute of Analytical Chemistry, 26545 TU Bergakademie Freiberg, Lessingstrasse 45, Freiberg 09599, Germany; ‡ Institute of Plant and Wood Chemistry, 9169 TU Dresden, Pienner Strasse 19, Tharandt 01737, Germany

## Abstract

Lignins are heterogeneous aromatic biopolymers whose structural complexity depends strongly on botanical origin and isolation process. Comprehensive characterization therefore typically relies on multiple complementary techniques, including gel permeation chromatography (GPC), Fourier transform infrared spectroscopy (FT-IR), pyrolysis-gas chromatography/mass spectrometry (Py-GC/MS), and nuclear magnetic resonance (NMR) spectroscopy, resulting in labor-intensive workflows and complex data integration. Here, ultrahigh-resolution Fourier transform ion cyclotron resonance mass spectrometry (FT-ICR-MS) is systematically benchmarked against these established methods using solvent-fractionated spruce kraft lignin as a model system. Complementary electrospray ionization (ESI) and graphite-assisted laser desorption/ionization (GALDI), combined with tandem mass spectrometry (MS/MS) and heterospectroscopic correlation analysis, reproduce fraction-dependent trends in oxygenation, aromatic condensation, and functional group distribution consistent with FT-IR, Py-GC/MS, and quantitative NMR data. While limitations remain for molar mass distributions and linkage quantification, FT-ICR-MS can serve as an advanced, high-throughput complementary screening platform that enables streamlined, comparative molecular-level lignin profiling with reduced analytical complexity.

## Graphical abstract

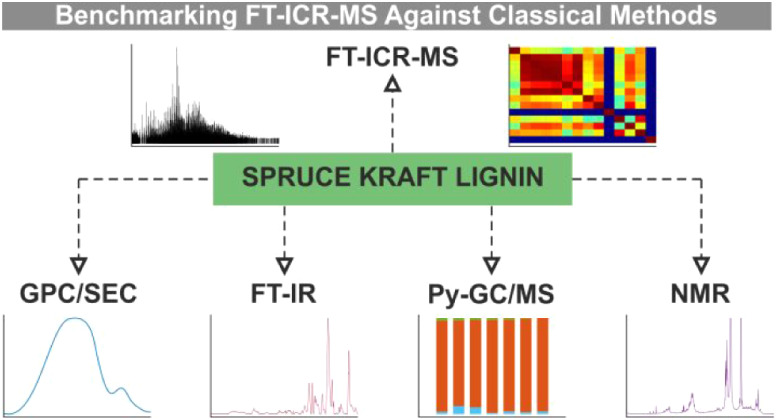

## 1 Introduction

Lignin is one of the most abundant renewable biopolymers on earth, accounting for up to 30% of the organic carbon in the biosphere and representing the second most abundant component of lignocellulosic biomass after cellulose.^1–3^ As an amorphous, heterogeneous polyphenolic macromolecule, lignin provides structural rigidity to plant cell walls and plays a key role in the resistance of plants against microbial attack.^2^ Beyond its biological role, lignin is increasingly recognized as a valuable resource for sustainable chemistry and material sciences. Due to its aromatic structure and rich functional group chemistry, lignin has attracted interest as a feedstock for renewable fuels, fine chemicals, resins, and advanced materials (hydrogels, depot fertilizers, humus substitutes).^4–10^ In the context of emerging biorefineries, the valorization of lignin is seen as an essential factor for improving the overall economics and sustainability of biomass utilization.^3,11^

Despite its abundance and potential, lignin remains underutilized compared to polysaccharides, largely because of its structural complexity. The chemical structure of lignin is not uniform but depends strongly on botanical origin, isolation process, and subsequent treatment. Softwood lignins are typically dominated by guaiacyl-type monolignols (G), while hardwood lignins also contain syringyl (S) structures and annual plants incorporate p-hydroxyphenyl units (H) in significant amounts.^12^ Technical lignins, such as kraft lignins, are further modified during industrial pulping processes, resulting in extensive structural heterogeneity, cleavage of interunit linkages, condensation reactions, and incorporation of sulfur functionalities.^2,12^ This variability hampers both the reproducibility of downstream applications and the establishment of reliable structure–property relationships. Consequently, an advanced analytical characterization of lignin is indispensable for both fundamental studies and applied research.^13–15^

Traditionally, the structural analysis of lignin relies on a combination of complementary analytical techniques. Nuclear magnetic resonance (NMR) spectroscopy is one of the most powerful tools in this context, offering detailed insights into interunit linkages, functional groups, and aromatic substitution patterns.^16–23^ Both one-dimensional and two-dimensional NMR methods,^24–29^ such as heteronuclear single quantum coherence (HSQC)^28,30,31^ and heteronuclear multiple bond correlation (HMBC),^29,32^ have been widely employed to elucidate lignin structure. However, the technique requires relatively large amounts of purified material, sophisticated instrumentation, and significant expertise in spectral interpretation. Furthermore, solubility limitations and insufficient sample homogeneity often complicate NMR analysis of lignins.^33,34^

Pyrolysis coupled with gas chromatography and mass spectrometry (Py-GC/MS) represents another widely used approach. By thermally fragmenting lignin into smaller volatile products, this method enables the identification and semiquantification of monomeric building blocks (H, G, S) and their derivatives. While informative, Py-GC/MS provides only indirect information about the native polymer structure, as the pyrolysis process induces secondary reactions and degradation artifacts. In addition, the technique primarily captures information on low-molecular-weight constituents, whereas much of the macromolecular complexity of lignin remains inaccessible.^35–39^

Gel-permeation chromatography (GPC), often utilized in its size-exclusion chromatography (SEC) form, is widely used to obtain molar mass distributions of lignin fractions.^40–42^ However, GPC results depend strongly on experimental factors including derivatization (e.g., acetylation), choice of solvent, reliable calibration standards, and data treatment, which all can lead to biases in reported molecular weights.^43,44^

Fourier transform infrared spectroscopy (FT-IR) has also been extensively applied to lignins. It is fast, nondestructive, and requires minimal sample preparation. FT-IR provides valuable information on functional groups, such as hydroxyl, carbonyl, and aromatic vibrations, and can be used for fingerprinting different lignin types.^16,32^ However, due to overlapping vibrational bands and limited resolution, FT-IR is not suitable for detailed structural elucidation. Instead, it is typically used in combination with other methods for comparative or qualitative purposes.^37^

The combined use of GPC, FT-IR, Py-GC/MS, and NMR reflects the general consensus that no single analytical technique is currently sufficient to provide a comprehensive description of lignin. While each method contributes unique insights, the need for multiple, often labor-intensive, techniques poses a significant bottleneck for routine lignin characterization. This situation has motivated researchers to explore alternative approaches that might deliver more holistic information from a single analysis.

In recent years, ultrahigh-resolution mass spectrometry (UHR-MS), particularly Fourier transform ion cyclotron resonance mass spectrometry (FT-ICR-MS), has emerged as a powerful tool for the characterization of complex natural organic matter.^2,3,45–48^ FT-ICR-MS provides unmatched mass resolving power and mass accuracy, enabling the assignment of molecular formulas to thousands of individual compounds in a single spectrum.^49,50^ This capability has revolutionized the study of dissolved organic matter, petroleum, and humic substances, and it is increasingly being applied to lignin and related biopolymers.^25,51–58^

The advantage of FT-ICR-MS lies in its ability to capture the immense molecular diversity of lignin at an unprecedented level of detail. By resolving thousands of isobaric species, it allows the construction of van Krevelen diagrams,^59,60^ Kendrick mass plots,^61–64^ and other visualization tools^25^ that map the chemical space of lignin constituents. Through such analyses, insights into oxygenation patterns, aromaticity, and degrees of unsaturation can be gained. Importantly, FT-ICR-MS does not require chemical derivatization or extensive fractionation, making it an attractive candidate for streamlining lignin analysis.^55,65^

However, despite its advantages, the role of FT-ICR-MS in lignin characterization remains under discussion. While the method excels at capturing molecular-level diversity and providing formula-level assignments, it inherently lacks information about connectivity, three-dimensional structure, and specific linkage motifs.^43,65^ This limitation raises the question of whether FT-ICR-MS can truly replace or only complement classical techniques such as NMR or Py-GC/MS. Another open question concerns how UHR spectral data can be translated into chemically meaningful information. While thousands of molecular formulas can be assigned, the link between these formulas and actual structural motifs remains indirect. Moreover, ionization biases, matrix effects, and the challenge of analyzing high-molecular-weight fractions all influence the scope of information obtained.^43,45,52^ These considerations underscore the need for careful benchmarking of FT-ICR-MS against established methods.

In this work, we systematically evaluate the extent to which FT-ICR-MS can serve as a central analytical tool for lignin fraction characterization by directly comparing it against established techniques, namely GPC, FT-IR, Py-GC/MS, as well as solution- and solid-state NMR. A fractionated spruce kraft lignin is used as a model system to generate chemically distinct lignin subdomains, enabling a structured comparison across methods. By combining complementary soft ionization approaches (ESI and GALDI), high-resolution molecular formula assignment, tandem MS fragmentation analysis, and chemometric heterospectroscopic correlation with NMR and FT-IR data, we demonstrate which structural trends and functional motifs traditionally derived from multiple classical techniques are reproducibly captured by FT-ICR-MS. At the same time, we delineate the methodological boundaries of FT-ICR-MS with respect to molar mass determination, linkage-specific information, and bulk structural quantification. On this basis, the study provides a critical assessment of how FT-ICR-MS can serve as a high-throughput complementary screening platform, and how its targeted integration can substantially reduce the time and analytical complexity associated with traditional multi-instrument workflows.

## 2 Experimental Section

### 2.1 Preparation of Kraft Lignin Fractions

The starting material was a commercially available spruce kraft lignin, Indulin AT (IAT), produced by Westvaco Inc. using the LignoBoost process.^12^ Fractionation was carried out using mixtures of deionized water and acetone (Carl Roth GmbH & Co. KG, purity: 99.9%) in order to separate polar and less polar lignin components. The extraction began with 100% (*v/v*) water (H_2_O) and the acetone content was subsequently increased in 10% (*v/v*) increments. Each extraction step was performed twice with the same solvent mixture to minimize variations caused by solubility limits. The procedure was continued until the remaining insoluble fraction was less than 5% (*w/w*), which occurred at a water:acetone ratio of 40:60 (*v/v*).

This sequential solvent fractionation strategy was specifically chosen to generate lignin subfractions with relatively narrow molar mass distributions and varying, polarity-dependent impurity profiles. Because the chemical differences between subfractions of a single parent lignin are much more subtle than those between lignins of different botanical origins, this sample series provided a highly challenging test set to evaluate the sensitivity of FT-ICR-MS in mapping minor compositional shifts.

Typically, 100 g of lignin was dispersed in 2 L of the respective solvent and stirred for 2 h at room temperature. The undissolved material was then separated by centrifugation for 30 min at 9000 rpm (Rotixa 50 S, Andreas Hettich GmbH). The resulting solutions were spray-dried using a Büchi 190 mini spray dryer equipped with a 0.5 mm nozzle, using an inlet temperature of 135–140 °C and a flow rate of 600 mL/min. Because the energy at the inlet is primarily consumed by solvent evaporation, the collected powder is not significantly heated, thereby preventing bond cleavage compared to isothermal drying.^66^ The insoluble residue was air-dried overnight, weighed, and subsequently utilized in the next extraction step with a higher acetone content.

For the characterization of the different lignin fractions, only the first extract of each water-acetone solvent system was evaluated, as no significant analytical differences were observed between the first and second extractions of the same solvent mixture.

### 2.2 FT-IR

FT-IR spectra of the spruce kraft lignin fractions were recorded using a Tensor 27 spectrometer (Bruker Optics GmbH) equipped with an attenuated total reflectance (ATR) diamond unit. Measurements were performed with 32 scans and a spectral resolution of 4 cm^–1^. Baseline correction was carried out using the *OPUS* software package (Version 7.2; Bruker Optics GmbH). The processed spectra were exported as .txt files and subsequently imported into *MATLAB R2025b* (The MathWorks, Inc.) for further data processing and visualization.

### 2.3 GPC

The molar mass distributions of the lignin fractions were determined by SEC. The spray-dried samples were dissolved in dimethyl sulfoxide (DMSO) (Carl Roth GmbH & Co. KG, purity: 99.99%) containing 1% (*w/w*) NaNO_3_, using a sample-to-solvent ratio of 1:1 (*w/w*). The solutions were shaken overnight at room temperature and subsequently filtered into vials through CHROMAFILXtra PTFE 45/20 syringe filters (Machery-Nagel GmbH & Co. KG).

GPC measurements were performed using an AZURA SEC system (Knauer Wissenschaftliche Geräte GmbH) equipped with the components P 6.1L, RID 2.1L, ASM 2.1L, and AS 6.1L. Separation was achieved using two size exclusion columns (Polargel M, Agilent Technologies; DMSO-Phil-P-250, AppliChrom GmbH) in combination with a Polargel M precolumn (Agilent Technologies). Analyses were conducted using a column temperature of 60 °C, a flow rate of 0.3 mL/min, and an operating pressure of 42 bar. Detection was carried out using a UV/Vis detector at a wavelength of 254 nm.

The system was calibrated with dextran standards in the molar mass range of 180–250 000 Da and a refractive index detector (RID). The detection unit was calibrated using Phenol Red. Data acquisition, processing, and evaluation were performed using *ClarityChrom 8.1* software (Knauer Wissenschaftliche Geräte GmbH).

### 2.4 Py-GC/MS

Py-GC/MS analyses were performed on approximately 250–400 μg per sample using an Agilent Technologies 7890D GC system coupled to a 5977 MSD. Pyrolysis was carried out at 450 °C with a Frontier Lab EGA/PY-3030D pyrolyzer. Separation of the pyrolysis products was achieved on a ZB-5MS capillary column (30 m × 0.25 mm) using a temperature program from 50 to 240 °C at a heating rate of 4 K/min. Compound identification was performed using the NIST 2014 mass spectral library, whereby only signals accounting for at least 0.5% of the overall peak integration area and substances with a library match score of at least 70% were further evaluated.

To enable a more detailed analysis of polar functional groups, such as hydroxyl and carboxyl moieties, the lignin fractions of higher polarity (water:acetone ratios 100:0–70:30 (*v*/*v*)) were subjected to silylation prior to GC/MS analysis. For this purpose, 10 mg of dried lignin sample was treated with 2 mL of 2 M HCl in methanol and heated at 100 °C for 2 h. After cooling, 100 μL of pyridine and 200 μL of an internal standard solution (150 mg phenyl-β-d-galactopyranoside in 10 mL pyridine) were added. From the resulting mixture, 1 mL of supernatant was transferred and dried overnight in a vacuum desiccator.

Subsequently, 500 μL of pyridine and 150 μL of the silylation reagent (bis­(trimethylsilyl)­acetamide (BSA) (Supelco, Inc.) and trimethylchlorosilane (TMSC) (Merck KGaA)) were added to the dried residue, and the mixture was stirred for 30 min at room temperature. The silylated samples were finally filtered through PTFE Rotilabo syringe filters 45/13 and transferred to vials for analysis.

### 2.5 NMR

Solution NMR spectra were recorded on a Bruker Avance Neo 700 spectrometer (Bruker Biospin GmbH & Co. KG) at 298 K [^1^H (700.3 MHz), ^13^C (176.1 MHz)]. A 5 mm BBO probe was used for quantitative ^13^C and DEPT ^13^C measurements and a TXI probe (^1^H, ^13^C, ^15^N) for ^1^H and ^1^H, ^13^C-HSQC experiments. Samples (150 mg) were dissolved in 700 μL dry DMSO-*d*_6_ (Armar GmbH, deutero 99.5 at. % D) and homogenized in an ultrasonic bath for 30 min. An aliquot of 600 μL of the resulting solution was transferred to a Young tube. HSQC spectra were recorded with 256 scans for each of the 512 increments, using 25% nonuniform sampling (NUS). For quantitative ^13^C spectra, Cr­(acac)_3_ (Merck Schuchardt OHG, for synthesis, purity ≥ 98%) was added to give a final Cr^3+^ concentration of 0.01 M,^18^ except in the case of the extract prepared with 100% (*v/v*) water. As confirmed by electron spin resonance (ESR) measurements, this sample contained manganese cations at a concentration which ensured sufficiently fast relaxation. A total of 20 k scans were accumulated using an inverse-gated pulse sequence with a repetition delay of 3 s. Chemical shifts were referenced to the solvent signal of DMSO-*d*_6_ at 39.5 ppm for ^13^C and to tetramethylsilane (TMS) at 0 ppm for ^1^H NMR.

Solid-state ^13^C NMR measurements were performed at 100.6 MHz on a Bruker AVIII HD WB 400 MHz spectrometer equipped with a 4 mm DVT CP/MAS probe. Samples were packed in 4 mm ZrO_2_ rotors and spun at 14 kHz. A single-pulse experiment was carried out on one sample to obtain a quantitative reference spectrum (90° pulse, 120 s recycle delay, 1 k scans) for CP optimization. The background Teflon signal was subtracted. For CP/MAS experiments (3 s recycle delay, 20 k scans), the contact time was optimized to reflect the quantitative composition as accurately as possible, resulting in a value of 7 ms with an 80% ramp on the ^1^H channel. All measurements were performed with an acquisition time of 15 ms and SPINAL64 decoupling. Chemical shifts were referenced externally to the CH_2_ signal of adamantane (38.5 ppm relative to TMS = 0 ppm).

### 2.6 FT-ICR-MS Analyses and Data Processing

All spruce kraft lignin fractions were characterized by FT-ICR-MS using both ESI and GALDI, enabling analysis of polar and less polar constituents, including soluble, poorly soluble, and insoluble components.^25,56^

For the ESI-FT-ICR-MS analyses, solid lignin fractions were weighed into amber glass vials, dissolved in a mixture of 60% (*v/v*) H_2_O and 40% (*v/v*) DMSO to obtain stock solutions of 1 g/L. These stock solutions were filtered through PTFE syringe filters (0.22 μm, VWR Chemicals) and then diluted to 200 mg/L with a solvent mixture composed of 80% (*v/v*) methanol, 19% (*v/v*) dichloromethane (DCM), 0.9% (*v/v*) H_2_O, and 0.1% (*v/v*) triethylamine (NEt_3_). The resulting analysis solutions were introduced into the ESI source at a constant flow of 10 μL/min using a 1 mL gastight syringe.

For the GALDI-MS analyses, 15 mg of each lignin fraction was mixed with 15 mg of high-purity graphite in amber glass vials, then suspended in 200 μL acetonitrile (ACN) containing 10 mM sodium carbonate (Na_2_CO_3_) and sonicated for 10 min. 1.2 μL of the suspension was spotted onto a MALDI steel target and allowed to air-dry.

All solvents used were of HPLC grade (Merck Chemicals, Carl Roth GmbH & Co. KG, Chemsolute, or VWR Chemicals); graphite powder (purity ≥ 99.9%, 5 μm) was obtained from Mikro to Nano, and Na_2_CO_3_ (purity ≥ 99.5%) from Sigma-Aldrich.

Mass spectrometric analyses were conducted on a 15 T solariX FT-ICR-MS (Bruker Daltonics) equipped with a dual ESI/MALDI source (MALDI: Smart Beam II, frequency-tripled Nd:YAG laser, λ = 355 nm, 3 ns pulse duration, 500 μJ pulse energy, 170 kW peak power, and 1.5 W average power). For all ESI-FT-ICR-MS analyses, the capillary voltage was set to +3,448 V with an end-plate offset of −500 V, nebulizer gas pressure of 1.0 bar, dry gas flow of 5.5 L/min, and a dry gas temperature of 255 °C, while GALDI-MS measurements employed an ultralarge laser focus with 20 laser shots, a plate offset of −60 V, a deflector plate voltage of −180 V, and a laser frequency of 500 Hz. All spectra were recorded in the negative ion mode over *m*/*z* 153.48–3000.00 with a Q1 mass of *m*/*z* 170. Data sets of 8 M were acquired by accumulating 256 scans with a time-of-flight (TOF) of 0.85 ms, and the resolving power was R = 800,000 at *m*/*z* 400.

Tandem MS experiments were conducted on 84 representative ions spanning a wide range (*m*/*z* 301.07–657.23) to capture the structural complexity of lignin. These ions were isolated with the quadrupole unit of the FT-ICR-MS, followed by collision-induced dissociation (CID) using argon as collision gas and collision energies (*CE*s) of 13–22 eV. Ion accumulation times (ESI-MS/MS), laser power, shot number (GALDI-MS/MS), and TOF settings were adjusted for each precursor ion individually. 64 scans were accumulated for the detection of product ions in a *m*/*z* range of 46.05–1000.00 using a data set size of 2 M.

Data calibration and evaluation of FT-ICR-MS and tandem MS experiments as well as the heterospectroscopic correlation of NMR and MS/MS data sets were conducted according to previously published routines.^25,56,67^

For each solvent-fractionated sample, the evaluated FT-ICR-MS and tandem MS results represent the average of the respective analytical acquisitions. The analytical reproducibility and instrument variance of the applied FT-ICR-MS and tandem MS workflows have been extensively described in previous studies.^68–70^

## 3 Results and Discussion

### 3.1 Fractionation Results

Fractionation of IAT was performed using solvent mixtures with acetone contents of up to 60% (*v/v*). Water-rich fractions usually dissolve polar molecules such as sugars and low-molecular-weight lignins. As the acetone content increases, the solvent polarity usually decreases, leading to an enhanced extraction of higher-molecular-weight lignins and nonpolar compounds like terpenes or fats (as supported by the Py-GC/MS results discussed below).

The resulting mass fractions of the individual extracts are summarized in Figure 1. Apart from the solvent mixture containing 70% (*v/v*) water (W) and 30% (*v/v*) acetone (A)), the amount of lignin dissolved in the second extraction step was consistently lower than in the first step. The largest fractions by mass were obtained with W:A ratios of 50:50 (*v/v*) and 40:60 (*v/v*), which together accounted for nearly half of the total recovered material.

**Figure 1:**
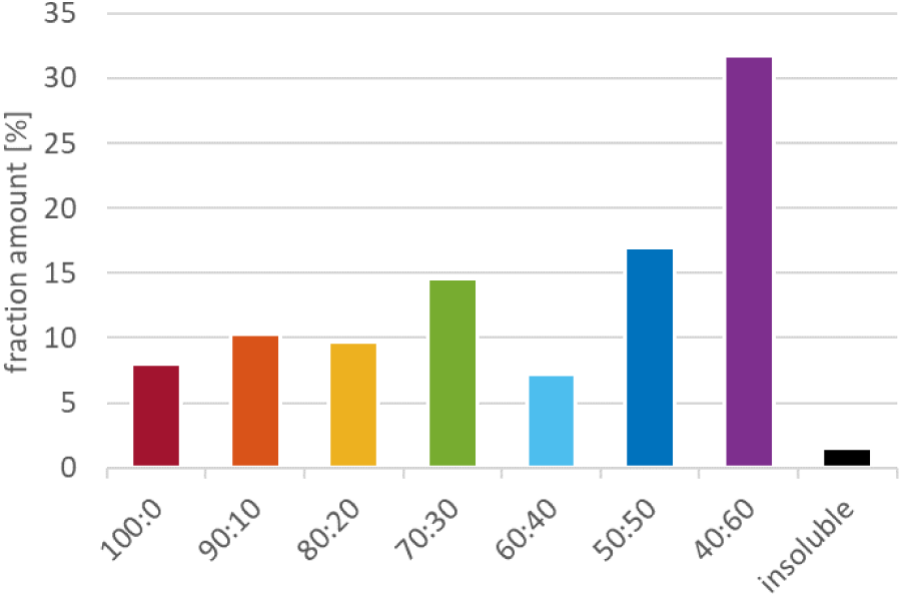
Mass fractions of the IAT fractions as a function of water (W):acetone (A) ratios (*v/v*).

### 3.2 Characterization of Spruce Kraft Lignin Fractions by FT-IR

The FT-IR spectra of the lignin fractions are shown in Figure 2. The fraction soluble in pure water (W:A 100:0 (*v/v*)) exhibits pronounced differences compared to the other samples. Several high-intensity bands at 1120–1130, 1032, and 1268 cm^–1^ can be attributed primarily to saccharides.^71–73^ In addition, the presence of a distinct band at approximately 900 cm^–1^, together with its splitting into two separate bands, indicates the presence of mono- or oligomeric species.^74^ Nevertheless, a significant absorption at 1515 cm^–1^ is also observed, confirming the presence of aromatic structures. This interpretation is further supported by characteristic guaiacol bands at 817 and 1325 cm^–1^ detected in the spectrum.^71–76^

**Figure 2:**
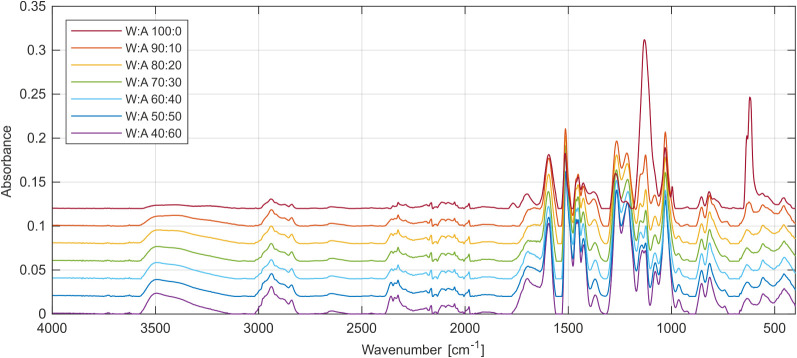
Baseline corrected FT-IR spectra for all analyzed spruce kraft lignin fractions. FT-IR spectra are plotted with an offset for visualization purposes.

Across the lignin fraction series, the relative contribution of aromatic compounds increases gradually with increasing acetone content up to 40% (*v/v*), as reflected by the *I*_1515_/*I*_1125_ ratio (see Table S1 in the Supporting Information), and then decreases slightly at higher acetone contents. Consequently, the highest aromatic purity is observed for fractions obtained with a W:A ratio between 80:20 (*v/v*) and 60:40 (*v/v*). For fractions containing more than 40% (*v/v*) acetone, the intensity of saccharide-related bands continues to decrease, while bands associated with aliphatic C–H stretching vibrations at 2840 and 2875 cm^–1^ become more pronounced. This behavior may indicate the presence of terpenes or fatty acids^73,77^ as impurities (also see Py-GC/MS results).

Although not characteristic of the native lignin backbone, the 2000–2500 cm^–1^ region exhibits weak signals relevant to technical lignins and process impurities.^73^ The band at approximately 2400 cm^–1^ corresponds to thiols introduced during the kraft process. Its decreasing intensity with higher solvent polarity is consistent with our Py-GC/MS results. While signals between 2350–2500 cm^–1^ can also indicate proteins, elemental analysis confirmed minimal nitrogen content, suggesting protein concentrations are below the detection limit, with any remaining amine bands obscured by overlapping lignin or carbohydrate signals. Absorption bands below 2400 cm^–1^ are primarily attributed to atmospheric components (primarily CO_2_), as ambient air cannot be entirely excluded during ATR sample preparation.

In the carbonyl stretching region, minor shifts in band positions are also evident. The fraction soluble in pure water (W:A 100:0 (*v/v*)) exhibits a band at approximately 1700 cm^–1^, which can be mainly assigned to acetyl groups originating from hemicelluloses.^71,75,76^ The intensity of this band decreases rapidly with increasing acetone content but rises again at acetone ratios of 50% (*v/v*) and above, likely due to the presence of lignin-carbohydrate complexes (LCCs).^78^ Simultaneously, a slight increase in the absorption at 1660 cm^–1^, attributed to alkenic C=C stretching vibrations in coniferyl units,^72^ is observed.

The strong peak at about 620 cm^–1^ can be attributed to C–S vibrations of alkyl sulfides, which are formed during the pulping process.^78^ The intensity of this band decreases with increasing acetone content, especially in water-rich fractions (W:A 100:0–80:20 (*v/v*)).

A detailed table summarizing the most relevant infrared absorption bands and their corresponding wavenumber regions for lignocellulosic materials is provided in the Supporting Information (see Table S2).

### 3.3 Molar Mass Distribution by GPC

The molar mass distributions of the main lignin fractions, determined by SEC, are presented in Figure 3. The curves shown correspond to the intensities recorded by the UV detector and therefore primarily reflect the aromatic components. All fractions exhibit multimodal distributions. The mass-averaged molecular weights (*M*_*w*_), corresponding polydispersity (*PD*) indices, and their relative proportions are summarized in Table 1. Although the positions of the peak maxima vary among the fractions, the average molecular weight generally increases with increasing acetone content, especially for the main peak (no. 3, see Table 1).

**Figure 3:**
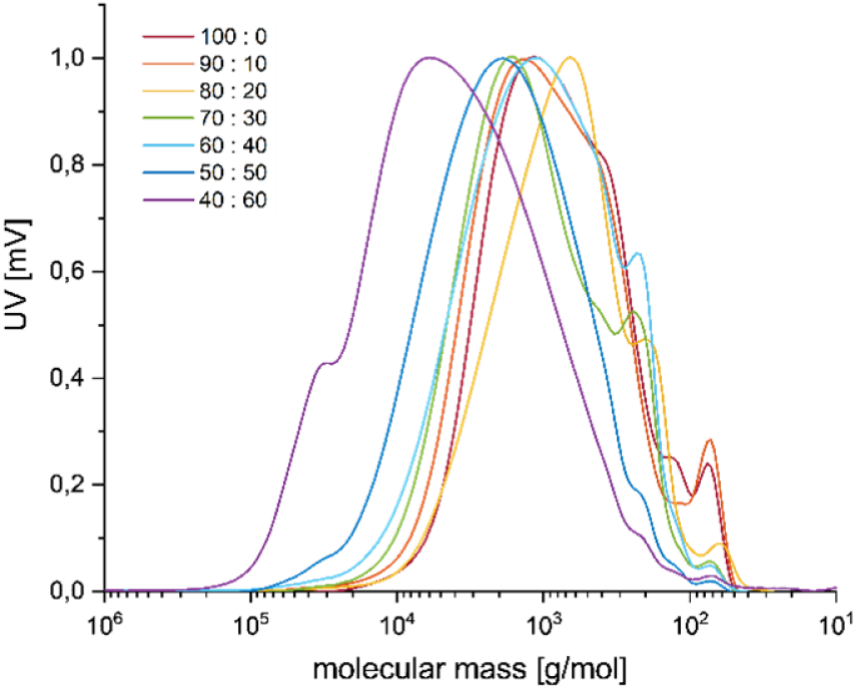
Molar mass distribution of spruce kraft lignin fractions (W:A solvent mixtures), as determined by GPC.

**Table 1:** Mass-Averaged Molecular Weights (*M_w_*), Polydispersity (*PD*) Indices, and Fractional Amounts by Molecular Weight Range of All Lignin Fractions Analyzed by GPC

		Fraction (W:A (*v/v*))						
		100:0	90:10	80:20	70:30	60:40	50:50	40:60
**Peak 1**	***M***_***w***_ [g/mol]	75	75	65	60	60	60	60
	***PD***	1.04	1.04	1.15	1.20	1.18	1.21	1.15
	**Amount [%]**	4.7	5.5	3.0	1.7	1.0	0.5	9.1
**Peak 2**	***M***_***w***_ [g/mol]			215	225	210		
	***PD***			1.07	1.09	1.18		
	**Amount [%]**			13.0	14.3	1.07		
**Peak 3**	***M***_***w***_ [g/mol]	1,625	2,000	2,045	2,805	2,860	4,585	6,983
	***PD***	2.86	3.00	2.16	2.29	2.8	4.11	3.99
	**Amount [%]**	92.3	94.5	84.0	84.0	86.3	99.5	90.3
**Peak 4**	***M***_***w***_ [g/mol]							60,295
	***PD***							1.25
	**Amount [%]**							0.6

It is important to note that the GPC data represent relative, apparent molar masses. Calibration against highly branched dextran was utilized as it better maps the hydrodynamic volume of lignin molecules than linear polystyrene. Consequently, significant but predictable deviations occur especially in the low molar mass range (e.g., peak 1 < 100 g/mol; see Section S3).

The molar mass distributions of the fractions soluble in pure water and W:A 90:10 (*v/v*) are very similar and are largely confined to values below 1500 g/mol. At W:A 80:20 (*v/v*), an additional low-molecular-weight peak appears, which is also present in fractions obtained with up to 40% (*v/v*) acetone. In particular, the fractions W:A 70:30 and 60:40 (*v/v*) show closely comparable distributions. A pronounced increase in average molecular weight is observed for fractions W:A 50:50 and 40:60 (*v/v*), with the latter containing a high molecular weight component exceeding 60 000 g/mol. Concurrently, the polydispersity of the main component increases in these acetone-rich fractions.

### 3.4 Py-GC/MS

The composition of the lignin fractions was also investigated by Py-GC/MS with particular focus on lignin content, the type and proportion of impurities, and the H/G/S ratio. Phenolic pyrolysis products classified as lignin-derived building blocks were selected based on literature data^39,79–82^ as well as complementary studies on monomeric and dimeric lignin model compounds. Other phenolic compounds were assigned to the category of aromatic extractives. An analogous classification approach was applied to the pyrolysis products originating from carbohydrates.

Due to the large number of compounds detected, a fully quantitative evaluation was not feasible. Therefore, relative peak areas were used for comparative analysis. Aromatic, phenolic, terpenoid, and fat- or wax-derived compounds exhibit comparable interactions with the GC column, whereas highly polar carbohydrates are underrepresented due to the low polarity of the utilized column. As a result, water-rich fractions, which were expected to contain most of the free carbohydrates, were additionally analyzed after silylation and in solution by GC/MS. In addition, only peaks that could be assigned to compounds in the NIST database with a match probability of at least 70% were considered.

Figure 4 summarizes the lignin content of the individual fractions as determined by Py-GC/MS. In all cases, the lignin content remains below 75%. Two distinct regions exhibiting a correlation between acetone content and lignin content can be identified, suggesting structural differences in the lignin fractions. In both regions, the lignin content increases with increasing acetone concentration. The lowest lignin content, approximately 60%, is observed for the fraction W:A 70:30 (*v/v*).

**Figure 4:**
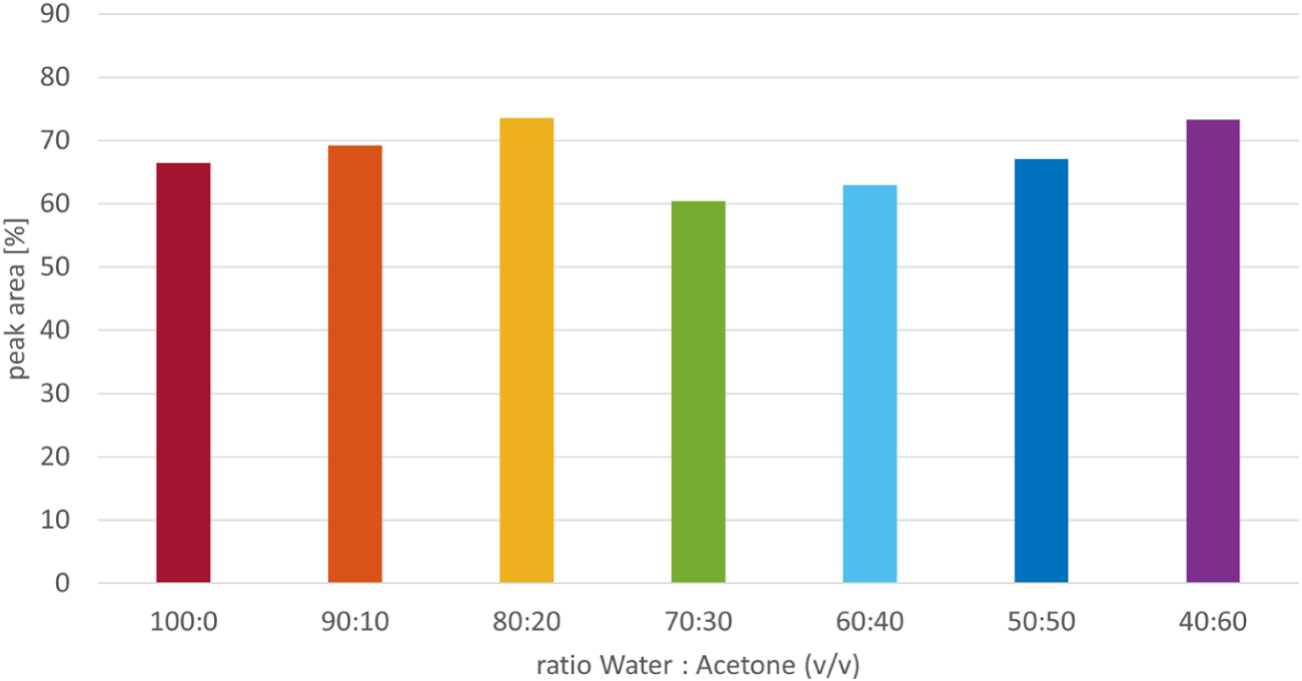
Lignin content of IAT fractions based on Py-GC/MS results and relative peak areas.

Furthermore, Figure S1 presents the H/G/S ratios of the lignin fractions. As expected for a softwood lignin, G units dominate, accounting for more than 94% of the total lignin. The remaining fraction consists of H and S units. The relative contributions of these units decrease slightly with increasing acetone content in the solvent mixture.

Pyrolysis of the lignin fractions yielded six predominant products, all originating from G building blocks. They are listed in Table 2 together with their relative abundances in the respective fractions. Only compounds with peak area contributions ≥1% are reported.

**Table 2:** Dominant Pyrolysis Products of IAT Fractions, Based on the Py-GC/MS Results

Relative amount [%]						
Pyrolysis product	Guaiacol	Creosol	2-Methoxy-4-vinylphenol	Vanillin	Vanillic acid	Coniferyl alcohol
**Molecular weight** [g/mol]	121.1142	138.1644	150.1754	152.1477	168.1467	180.2011
100:0	18.36	2.94	5.94	2.08	17.53	-
90:10	23.63	3.38	6.27	5.86	12.78	-
80:20	30.35	4.64	8.20	3.08	5.26	-
70:30	21.19	5.21	9.19	2.92	3.45	4.28
60:40	15.54	7.51	7.65	3.61	3.96	3.78
50:50	11.31	9.45	7.53	2.77	4.39	4.13
40:60	11.19	10.90	9.40	3.18	5.09	5.83

The relative proportions of these pyrolysis products vary with the acetone content of the solvent system. In acetone-rich fractions, a pronounced decrease in the abundance of guaiacol is observed. Its high concentration in fraction W:A 100:0 (*v/v*) indicates the presence of this compound primarily as monomer in this fraction. However, guaiacol is also a characteristic pyrolysis product of β-O-4 and β-β′ linkages of the resorcinol type in lignin dimers, as shown in own model compound experiments, which explains the initial increase in its proportion at low acetone contents. These fractions are therefore assumed to consist mainly of monomeric and smaller oligomeric lignin species (also see GPC results).

Furthermore, the proportion of creosol increases steadily with rising acetone content. Creosol is a typical pyrolysis product of dimeric β-β′ linkages of the secoisolariciresinol type, as evidenced in our own model compound experiments.

For both, vanillin and guaiacol, an initial increase in relative abundance is observed, followed by stabilization at an approximately constant level. Vanillin is primarily formed during the pyrolysis of lignin dimers containing 5–5′ linkages (own model experiments), which are known to occur more frequently in coniferous lignin.^12^

No specific dimeric linkage could be clearly assigned to one of the main pyrolysis products, 2-methoxy-4-vinylphenol. Since pyrolysis behavior is influenced by a range of structural factors beyond simple monomer units, the development and investigation of additional oligomeric model compounds, comprising at least three monomer units and incorporating diverse linkage types, are required to further elucidate lignin structures based on pyrolysis products.

A range of impurities was identified in the lignin fractions by analysis of the Py-GC/MS chromatograms (also see Figure 5). These impurities were categorized into sulfur-containing compounds originating from kraft pulping, carbohydrates, nitrogen-containing compounds, aromatic extractives, condensed hydrocarbons, terpenes (lipids), and fatty acids (lipids). The sulfur-containing species, mainly methyl sulfides and methanethiol, show a decreasing trend with increasing acetone content in the extraction solvent, due to the polarity of these functional groups.

**Figure 5:**
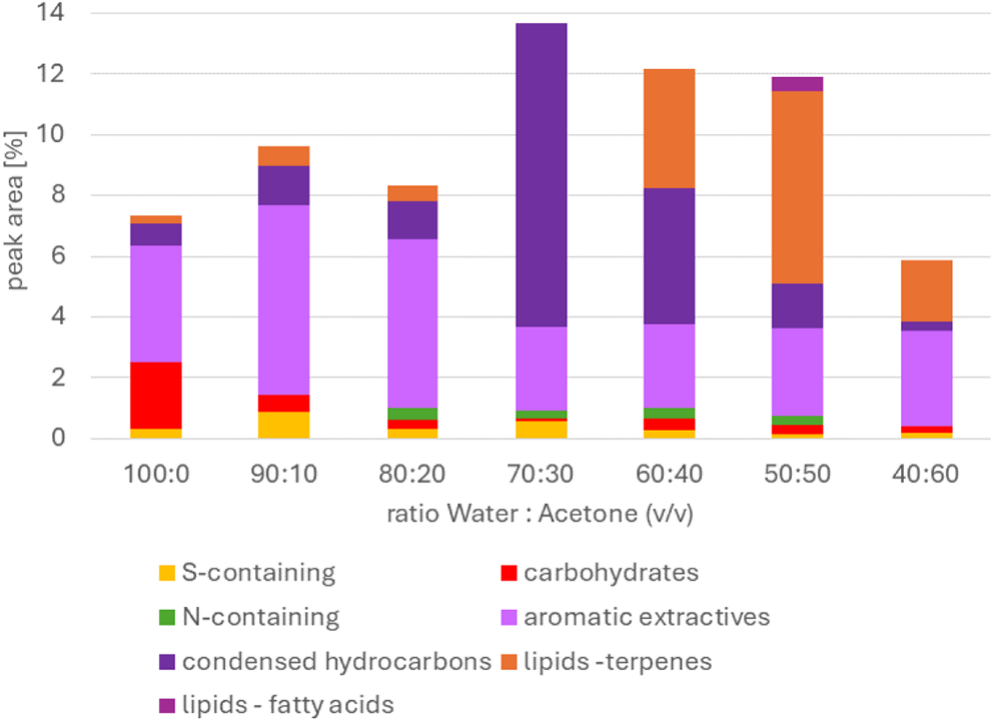
Impurity types and contents of spruce kraft lignin fractions as determined by Py-GC/MS.

As expected, carbohydrates are most abundant in fractions obtained with water-rich solvent mixtures, and the proportion of aromatic extractives decreases in the same direction. Monomeric aromatic compounds are therefore assumed to be preferentially present in these water-rich fractions. A notably high content of condensed hydrocarbon compounds is observed in the fractions W:A 70:30 and 60:40 (*v/v*). This effect is largely attributed to naphthalene- and phenanthrene-derived compounds. Light components formed during low-temperature pyrolysis, such as phenols and guaiacol, can act as precursors for polycyclic aromatic hydrocarbons (PAHs). However, PAH formation typically occurs at higher temperatures.^35,83^ Another major condensed aromatic compound is 6-hydroxy-2″,3′,4″-trimethoxy-1,1′-biphenyl, which primarily occurs in fraction W:A 90:10 (*v/v*). It is likely formed via condensation of low-molecular-weight lignin products. Alternatively, it may also originate as a pyrolysis product of 5–5′ linked lignin units.

At an acetone content of 40%, terpenes become increasingly prominent, with abietic acid derivatives being the dominant species. In addition, small amounts of octadecenoic acids are detected in the two acetone-richest fractions, indicating the presence of fatty acid-derived impurities.

The chromatograms of the silylated samples reveal the presence of TMS derivatives of both carbohydrate and lignin components, with the lignin-derived compounds accounting for approximately twice the proportion of the carbohydrate-derived species. Figure S3 compares the total peak areas obtained from Py-GC/MS analysis of the lignin fractions with those from GC/MS analysis of the silylated samples. The comparison shows that the contribution of carbohydrates detected after derivatization is negligible and can therefore be disregarded. The GC/MS investigations were therefore limited to the first fractions (W:A 100:0 to 70:30 (*v/v*)), where the highest amounts of free carbohydrates were expected, as the aim was to confirm the comparatively low carbohydrate content. In addition to the inherently low carbohydrate content of IAT, this observation suggests that only a fraction of the carbohydrate moieties remains as intact saccharides after the pulping process, while a substantial portion has already undergone structural modification.

### 3.5 NMR Characterization of the Lignin Fractions

Solution- and solid-state NMR spectroscopy were further employed to characterize the overall structural composition of the lignin samples. For the dissolved samples, ^1^H, quantitative ^13^C, DEPT, and ^1^H, ^13^C-HSQC spectra were recorded. The quantitative ^13^C measurements enable estimation of the relative proportions of structural elements in the lignin fractions, following the approach of Capanema et al.,^18^ while the two-dimensional (2D) NMR experiments provide more detailed structural differentiation.

Figure 6 presents a comparison of the ^13^C NMR spectra of the fractionated lignins. The corresponding ^1^H NMR spectra are provided in the SI (see Figure S4).

**Figure 6:**
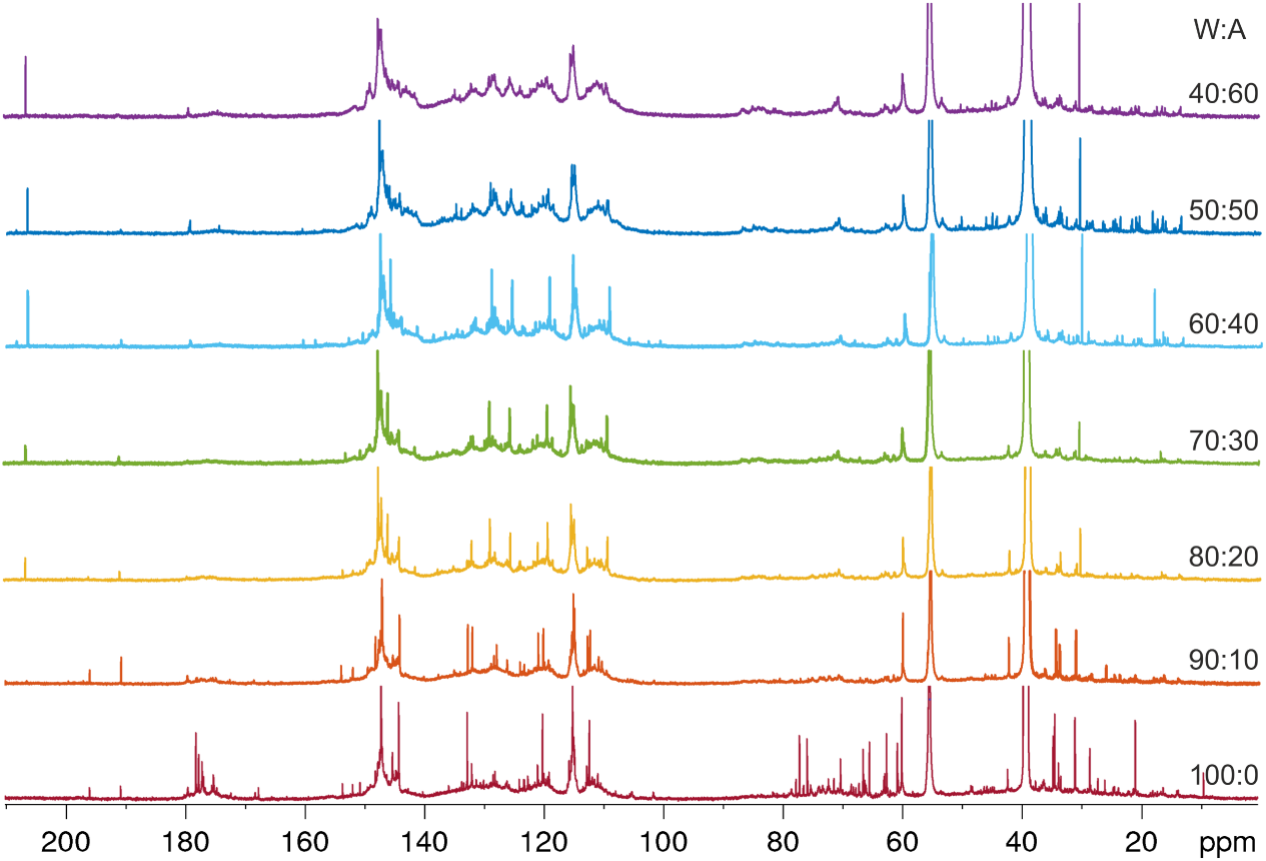
Quantitative ^13^C NMR spectra of fractionated spruce kraft lignins in DMSO-*d*_6_ with decreasing polarity of the solvent extraction mixture, scaled to the intensity of the methoxy signal at 56 ppm. The signal intensity is cut off for the methoxy carbons (56 ppm, at 50%) and the solvent (DMSO-*d*_6_, 39.5 ppm). The signals at 30.6 and 206.5 ppm originate from acetone.

As expected, the spectra exhibit signal groups in similar chemical shift ranges. Signals corresponding to ketones and aldehydes appear with very low intensity between 200 and 185 ppm. Carbonyl groups of acids and esters are observed from 185 to 165 ppm. Here, the high proportion of carboxylic acid groups in fraction W:A 100:0 (*v/v*) is particularly notable, reflecting the preferential extraction of the respective compounds by the most polar solvent. In addition to the acidic groups in lignins, these groups are often bound to hemicelluloses, which corresponds to the large share of carbohydrate-based structures detected as impurities by FT/IR and Py-GC/MS (also see Figures 2 and 5). The region between 165 and 100 ppm is characteristic of aromatic carbon environments and is dominated by oxygen-substituted structural units just below 150 ppm. A typical feature of spruce lignin, which consists predominantly of G units, is the almost complete absence of aromatic signals below 110 ppm, where the C_2_ and C_6_ positions of S units would normally resonate.

The aliphatic carbon region below 90 ppm is dominated by the signal of methoxy groups attached to the aromatic ring. At δ < 50 ppm, only a limited number of signals with low intensity are expected from lignin structures. The presence of additional signals in this region indicates various accompanying components. More polar fractions are dominated by signals attributable to alkyl residues of carboxylic acids. In contrast, the less polar extracts display a large number of signals with similar intensity in the 40–20 ppm region, suggesting the presence of terpene-derived compounds, consistent with the Py-GC/MS results. Analyses of a commercial colophony (Cello Rosin by Royal Oak, Inc.) obtained from spruce wood, which contains abietic acids (diterpenoid), their derivatives, and related isomers, produced spectra with very similar signal patterns throughout the NMR spectrum. Signals attributable to abietic acid could be identified on the basis of literature data.^84,85^

In addition, fraction W:A 100:0 (*v/v*) is characterized by numerous signals between 80 and 60 ppm, which are typical of C–OH and C–OR groups in carbohydrates, which again is consistent with the Py-GC/MS results.

Overall, a progressive increase in line width and a reduction in the number of sharp signals are observed with decreasing polarity of the extraction solvent, particularly in the aromatic carbon region. This behavior confirms the results of GPC and Py-GC/MS, that extracts obtained with more polar solvent mixtures contain a higher proportion of smaller molecules and oligomers, whereas less polar solvents increasingly solubilize larger molecules in which polar functional groups are incorporated in between lignin subunits.

The signal intensities of the quantitative ^13^C NMR spectra were further evaluated following the procedure of Capanema et al.^18^ (for more details, see Section S6). Minor modifications were made to the integration routine originally proposed for milled wood lignins in the literature:^18^ (i) the carboxylic acid region was expanded by introducing an additional subregion extending up to 181 ppm, and (ii) DEPT measurements revealed that the subregion from 140–125 ppm also contains small amounts of aromatic CH groups, rather than exclusively quaternary aromatic carbons, while the region from 77–65 ppm includes CH_2_ groups, likely originating from nonlignin components (also see Figure S5). The assignment and nomenclature of structural elements were performed in accordance with previously published studies and are given in Table S4.^17–19,24,27,28^

Figure 7 illustrates the intensity distributions of selected structural groups for the different extraction mixtures, while Figure S6 provides an overview of all spectral regions evaluated.

**Figure 7:**
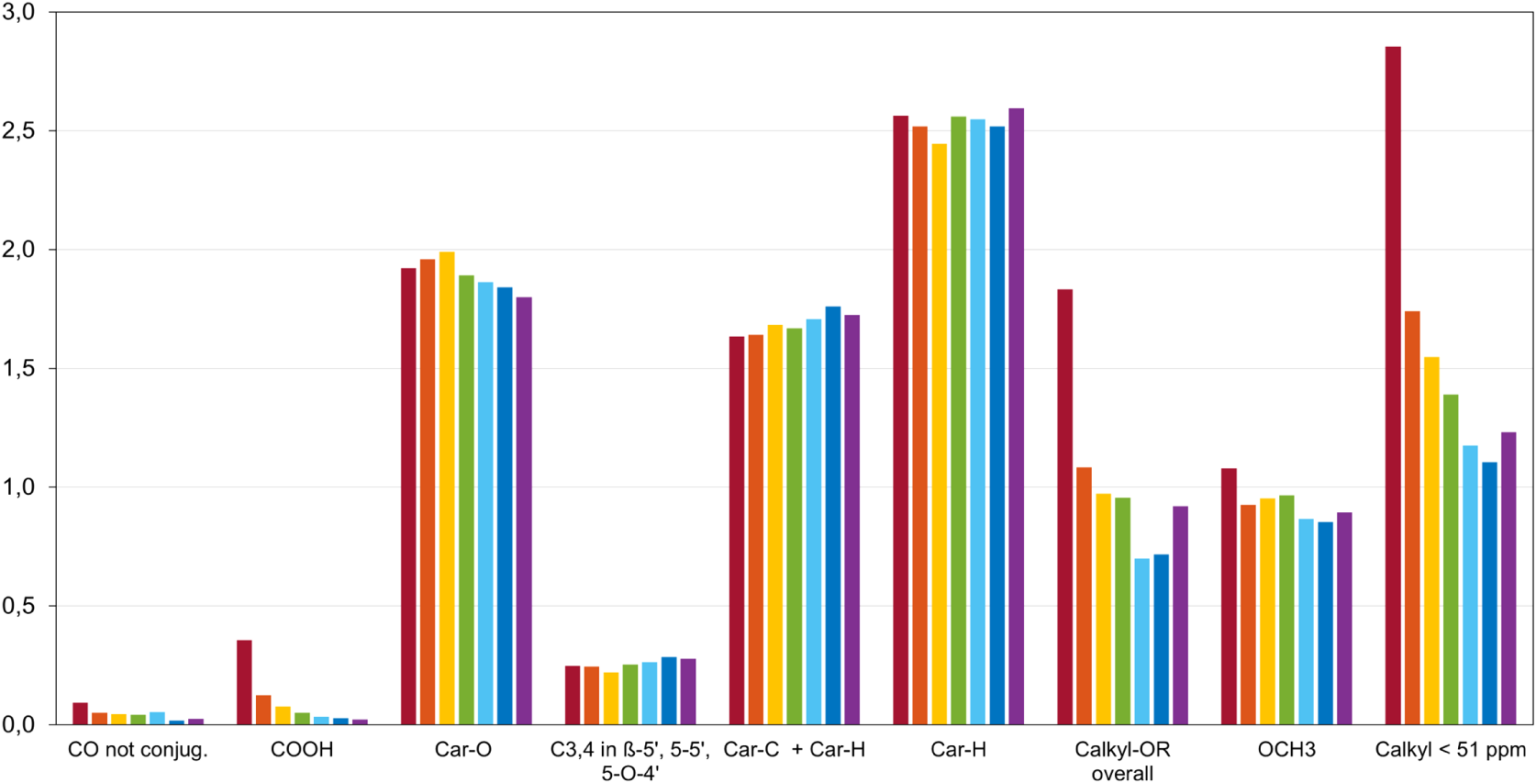
Distribution of selected structural elements within the discussed spruce kraft lignin fractions according to quantitative ^13^C NMR analyses, scaled to the intensity of the aromatic and vinylic carbon atoms (100–165 ppm, *I* = 6.12): From left to right: W:A 100:0, 90:10, 80:20, 70:30, 60:40, 50:50, 40:60 (*v/v*).

In the carbonyl region, a clear reduction of these structural elements is observed as the polarity of the extraction mixture decreases. Furthermore, in the aromatic region, the proportion of C–O structures declines slightly, whereas the proportion of C–C bonds increases. Both trends reflect the progressively higher degree of cross-linking and molar mass in the lignin fractions extracted with higher acetone amounts, resulting from the formation of additional C–O–C and C–C linkages. In the aliphatic structural region, a markedly higher content of aliphatic structures, especially in water-rich fractions, is evident, particularly for fraction W:A 100:0 (*v/v*). These signals mainly originate from secondary and primary alcohol units as well as ester functionalities within the carbohydrates as identified by the previously discussed methods.

The relative content of methoxy groups decreases slightly with decreasing solvent polarity, further indicating enhanced cross-linking within the lignin structure. Nevertheless, values just below one relative to the aromatic base units remain characteristic of G-type lignin, as the underlying monomer guaiacol contains one methoxy substituent. In the least polar fraction W:A 40:60 (*v/v*), the intensity of signals below 51 ppm increases again, reflecting the presence of abietic acid and related compounds identified in this sample.

The assignment in the ^13^C NMR spectra was also supported by 2D ^1^H, ^13^C-HSQC measurements. The main structural groups observed are illustrated for fraction W:A 70:30 (*v/v*) in Figures S9–S11. Overall, the analyses corroborate the Py-GC/MS results, which indicate that the lignin fractions consist predominantly of G structural units.

Because of the relatively low detection sensitivity of NMR spectroscopy for ^13^C nuclei, comparatively concentrated solutions are required for the analysis of lignins. To ensure that the solution-state measurements fully represent the entire sample and are not affected by solubility limitations, solid-state NMR analyses were additionally performed for all fractions. Although the spectral resolution in solid-state NMR is inherently lower, similar trends for the key structural elements were observed as a function of solvent mixture polarity. This confirms that the liquid-state NMR results are representative of the material investigated. Solid-state NMR data are provided in the SI in Figures S7 and S8.

### 3.6 Mass Spectrometric Characterization of Spruce Kraft Lignin Fractions

To evaluate the potential of UHR FT-ICR-MS as a stand-alone and integrative tool for lignin characterization, all solvent-fractionated spruce kraft lignin samples were analyzed using two complementary soft ionization techniques, ESI and GALDI, and compared to the results obtained by conventional analytical methods. The combination of both ion sources was selected to maximize chemical space coverage, as lignin oligomers differ strongly in polarity, aromaticity, and functional group density.

The overview spectra of all fractions acquired by ESI(−)-FT-ICR-MS and GALDI(−)-FT-ICR-MS are shown in Figure 8. In general, both ionization techniques covered a comparable mass range of approximately *m*/*z* 200–1000, demonstrating that all fractions contain complex mixtures of lignin-derived mono- to oligomeric species rather than only low-molecular weight fragments. Importantly, systematic differences between fractions were observed, which follow the general fractionation trends derived from GPC and spectroscopic analysis, presented previously. Fractions obtained with increasing acetone content displayed a tendency toward a broader distribution and stronger signals at higher *m*/*z* values, indicating an enrichment of larger and more structurally diverse oligomeric species. This finding is consistent with the GPC results, where acetone-rich fractions were associated with increased molecular weight and altered molar mass distributions. Nonetheless, all presented data show that FT-ICR-MS does not fully reflect the molar mass distribution of lignin fractions obtained by GPC, because only a chemically and physically biased subset of molecules is naturally detected by FT-ICR-MS. Especially varying ionization efficiencies, mass-dependent transmissions, aggregation, and in-source fragmentation favor low-molar-mass, polar species, leading to an underrepresentation of higher-molar-mass components. Furthermore, the lack of commercially available, large well-defined lignin oligomer standards (e.g., trimers and tetramers) currently prevents the determination of absolute response factors to fully account for matrix effects and signal suppression. Nevertheless, FT-ICR-MS still provides detailed access to molecular trends and structural characteristics within the detectable low-molar-mass lignin species. For a more robust comparison with the GPC results, the number-average (*M*_*n*_), weight-average (*M*_*w*_), and peak (*M*_*p*_) molar masses calculated from the FT-ICR-MS data sets are provided in Table S5.

**Figure 8:**
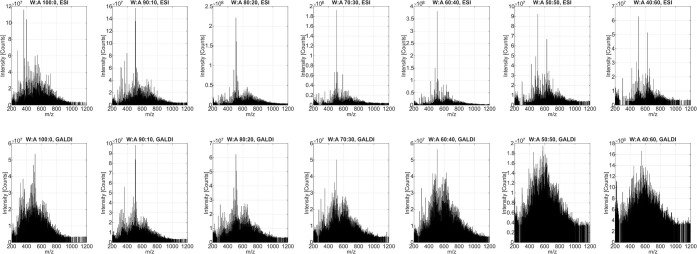
ESI­(−)- and GALDI(−)-FT-ICR-MS mass spectra of all analyzed fractions of the spruce kraft lignin, generated by different water/acetone solvent mixtures (W:A 100:0–40:60 (*v/v*)).

Furthermore, a quantitative comparison of spectral characteristics (peak number, total ion current (TIC), and mean *m*/*z*; see Section S10 in the SI) further highlights method-specific selectivity effects. For ESI-MS, peak number, TIC and mean *m*/*z* increased from W:A 100:0 (*v/v*) to 80:20 (*v/v*) and decreased toward 40:60 (*v/v*), with an additional pronounced increase for the 60:40 (*v/v*) fraction. In contrast, GALDI-MS provided more comparable peak numbers and TIC values across the fractions while showing a slight increase in mean *m*/*z* with increasing acetone content. This supports the interpretation that GALDI enables enhanced access to larger and less polar lignin oligomers, while ESI preferentially detects smaller, more oxygenated, and readily deprotonated structures.^25,56^

To enable a chemically intuitive interpretation beyond mass distribution alone, van Krevelen analysis (see Section S11 in the SI) was further applied to the assigned molecular formulas, and the intensity-weighted compound class composition^59^ is summarized in Figure 9. For all fractions and both ionization methods, the dominant contribution was attributed to the lignin region, confirming that all fractions remained highly lignin-rich after solvent fractionation. Despite the overall lignin dominance, subtle but systematic trends between fractions were evident. In particular, the acetone-rich fractions showed increasing contributions from condensed hydrocarbons, especially for the GALDI-MS data sets, indicating a compositional shift toward structures with lower oxygen content and increased aromaticity/condensation. This finding provides molecular-level support for the trends discovered by Py-GC/MS and NMR, where later fractions were interpreted as being enriched in more condensed and less polar lignin motifs. In addition, minor contributions from lipids, carbohydrates, and related classes were detected at low levels by ESI- and GALDI-FT-ICR-MS in all fractions, suggesting that the fractionation primarily reorganizes lignin-intrinsic chemical subdomains rather than introducing substantial nonlignin contaminants.

**Figure 9:**
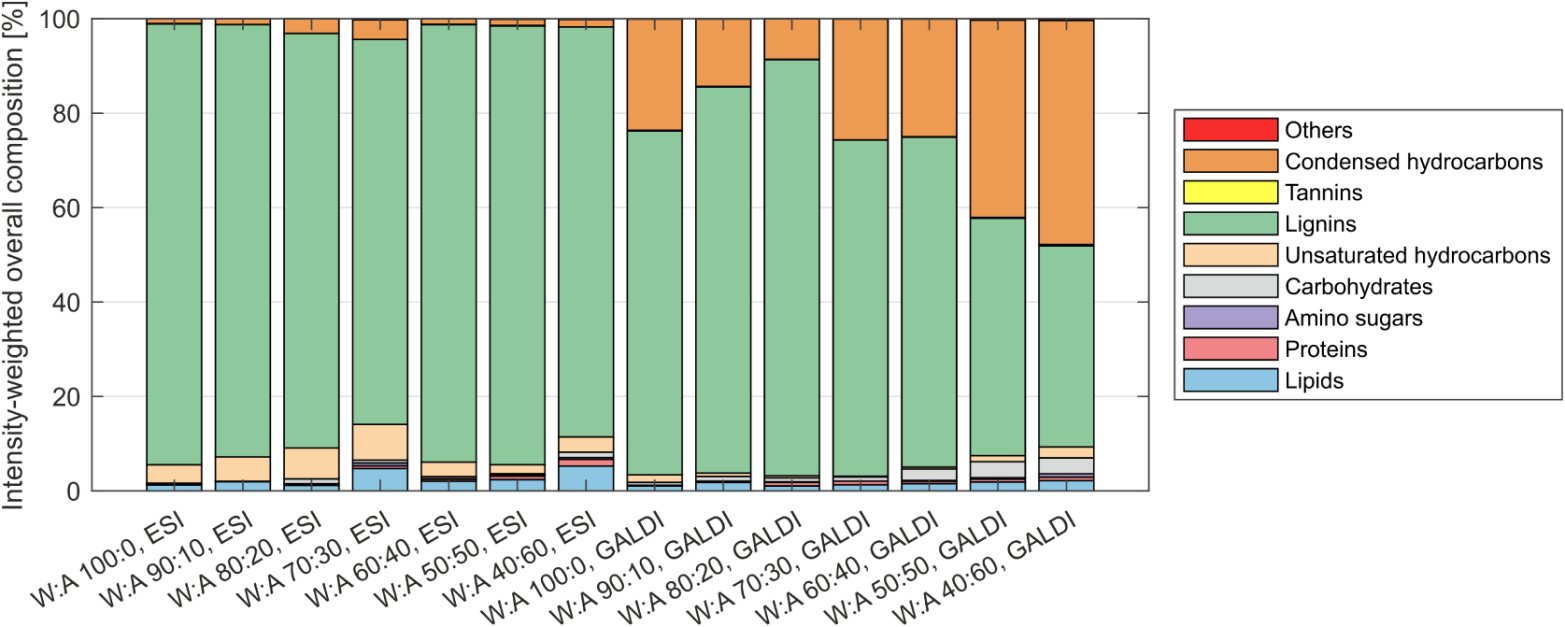
Intensity-weighted relative composition of the different spruce kraft lignin fractions, as calculated according to the van Krevelen plots of the FT-ICR-MS data (also see Figure S13). The compound classes and their respective regions were adapted from literature.^59^

Furthermore, a statistical comparison of heteroatomic classes is presented in Figures S15 and S16, showing the total number of assigned molecular formulas and the relative abundance distribution to the detected biopolymer ions. Across all fractions, oxygen-only classes clearly dominated, consistent with the oxygen-rich nature of kraft lignin due to abundant hydroxyl, ether, and methoxy functionalities. The most frequent oxygen classes occurred across a broad range (O_3_–O_16_), reflecting the mixture of structurally diverse lignin-derived oligomers detected in each fraction.

The fraction-dependent oxygenation patterns also fit well into the interpretation derived from previous analyses: the water-rich fractions were characterized by strong contributions of oxygen-rich classes, whereas acetone-rich fractions showed a more pronounced representation of less oxygenated and structurally condensed compositions. This is consistent with the progressive shift toward more aromatic/condensed structures observed in the van Krevelen class composition (also see Figure 9), and it reinforces the functional group trends discussed previously (e.g., fraction-dependent variation in oxygenated lignin substructures as observed by Py-GC/MS and NMR).

In addition to oxygen-only classes, sulfur-containing classes were detected and showed strong ion source dependence. ESI-MS exhibited the highest contribution of sulfur-containing molecular formulas (notably S_1_O_8_–S_1_O_12_ classes), while GALDI-MS detected sulfur-containing species only at low levels. This behavior is consistent with the stronger sensitivity of ESI toward polar and highly oxygenated kraft-lignin-related structures. In the context of the research presented in this paper, this observation provides complementary evidence that solvent fractionation leads to an enrichment of highly polar (and potentially kraft-process-modified) lignin components in the water-rich fractions, while acetone-rich fractions are increasingly dominated by less polar and more condensed lignin populations.

These trends are also structurally verifiable using plots of the carbon number (*n*_*C*_) vs the double bond equivalent (*DBE*) for specific heteroatomic classes. As an example, *n*_*C*_-*DBE* plots for classes O_9_–O_12_ are visualized in Figures S17 and S18. The GALDI-MS analyses reveal a higher number of detected data points and generally higher *n*_*C*_ and *DBE* values than ESI-MS, indicating, again, that GALDI preferentially accesses larger, more unsaturated, and less strongly functionalized (i.e., less polar) lignin-derived molecules, whereas ESI is biased toward smaller, highly functionalized and more polar species. Across both ionization techniques, an overall increase in *n*_*C*_ and *DBE* values with increasing fraction number is observed, demonstrating that larger and highly aromatic lignin structures are progressively enriched with increasing acetone content, with sulfur- and oxygen-containing as well as purely oxygen-containing molecules extending into highly aromatic regions (ESI: *DBE* ≤ 30; GALDI: *DBE* ≤ 40).

To further understand structural differences between fractions beyond elemental composition, MS/MS experiments were performed and evaluated using 35 lignin-characteristic product ions.^25,57,58,86^ The overall number of detected product ions and their summed intensities for both ionization techniques are compiled in Figure 10. Across all fractions, the most frequently detected neutral losses included CH_3_, CH_4_, 2 × CH_3_, OH, H_2_O, CH_2_O, CH_4_O, CO_2_, HCOOH, and combined losses such as CH_3_ + CHO and CH_3_ + HCOOH. These fragmentation products are fully consistent with lignin-derived oligomers containing methoxy and hydroxyl functionalities and thus support the G-rich structural character expected for spruce kraft lignin. These lignin structures are therefore most likely interconnected predominantly through β-O-4′ and/or β-β′ linkages.^57,58,86^

**Figure 10:**
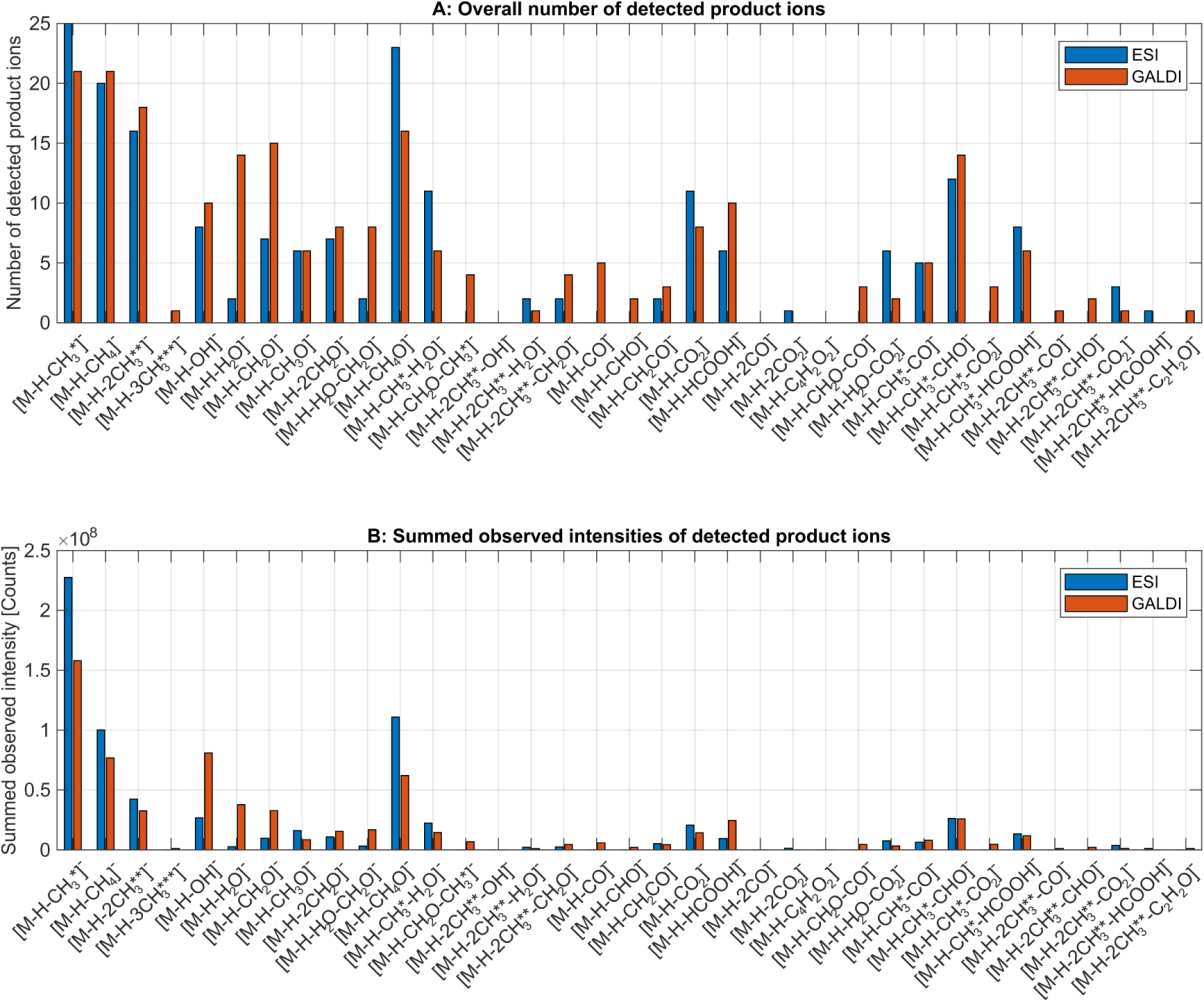
Comparison of the overall number (A) and summed observed intensity (B) of the 35 lignin characteristic product ions, as detected by ESI- and GALDI-FT-ICR-MS/MS for 42 isolated precursor ions (six ions in seven fractions) per ionization source.

Beyond confirming the general lignin structural backbone, the MS/MS results also refine the fractionation narrative established earlier. Polar solvent fractions showed fragmentation patterns that more frequently involved carboxyl-related losses (e.g., CO_2_ and HCOOH), suggesting a higher abundance of oxygenated and more polar structural motifs. This is consistent with the higher representation of oxygen-rich classes in ESI (also see Figure S15) and supports the interpretation from FT-IR, GPC, Py-GC/MS and NMR that these fractions contain more functionalized lignin substructures. In contrast, acetone-rich solvent fractions were comparatively enriched in condensed aromatic compositions (also see Figure 9), and the MS/MS product ion patterns increasingly reflected fragmentation pathways associated with methoxy/methyl losses and reduced prominence of carbonyl-related neutral losses, consistent with a higher contribution of structurally condensed motifs. Notably, carbonyl/aldehyde-related neutral losses were far more pronounced in GALDI-MS/MS than in ESI-MS/MS, indicating that GALDI accesses a lignin subpopulation with higher aromaticity and lower polarity that is less efficiently ionized under ESI conditions. Accordingly, the MS/MS fragmentation provides an additional structural dimension by correlating fraction-specific molecular formula populations with characteristic neutral loss pathways, demonstrating that the applied solvent fractionation yields chemically distinct spruce kraft lignin subdomains rather than a simple redistribution of molecular size.

Furthermore, in the SI (see Section S14), a correlation heatmap is provided that visualizes the similarities and differences in fragmentation patterns among the selected *m*/*z* values across the fractions. Overall, this MS/MS similarity analysis shows that fragmentation patterns are largely conserved across fractions and ionization techniques for several key precursor ions, most notably at *m*/*z* 301.07 (C_16_H_14_O_6_, [M–H]^−^) and 351.12 (C_21_H_20_O_5_, [M–H]^−^). However, deviations, particularly for fraction W:A 100:0 (*v/v*) under ESI conditions and for higher-mass ions with limited product-ion statistics, indicate increased structural complexity and/or compositional heterogeneity in specific fractions rather than fundamentally different fragmentation mechanisms.

To further illustrate the robustness of the observed fragmentation trends, a representative precursor ion, *m*/*z* 507.17 (C_28_H_28_O_9_, [M–H]^−^), that was consistently detected in all analyzed lignin fractions by both ESI- and GALDI-FT-ICR-MS was examined (see Section S15 in the SI). Under ESI conditions, strong similarities in fragmentation behavior are observed between fraction W:A 90:10 (*v/v*) and all subsequent fractions with higher acetone content, whereas fraction W:A 100:0 (*v/v*) shows distinctly lower similarity due to the occurrence of additional carboxyl-related product ions (e.g., [M–H–CO_2_]^−^, [M–H–HCOOH]^−^, [M–H–2CO_2_]^−^), indicating a higher abundance of carboxyl-functionalized structures in the water-rich fractions and a progressive shift toward less externally oxygenated, more condensed motifs with increasing acetone content. For GALDI-MS/MS, high similarities are mainly observed between the fractions W:A 100:0 (*v/v*) and 90:10 (*v/v*) as well as among W:A 90:10 (*v/v*), 60:40 (*v/v*), and 50:50 (*v/v*). Pronounced differences, particularly the strong separation of the fractions W:A 70:30 (*v/v*) and 50:50 (*v/v*) and the increased prominence of methyl-loss and aldehyde-related fragmentations in acetone-richer fractions, highlight a clear structural transition occurring around fraction W:A 80:20 (*v/v*) and 70:30 (*v/v*). This is also schematically reflected by two structure proposals for the molecule C_28_H_28_O_9_, as visualized in Figure S24. However, it must be noted that assigned molecular formulas can correspond to multiple structural isomers, and isomerism as well as collision-energy-dependent fragmentation complicate absolute structural determinations. Therefore, the synthesis of targeted, larger oligomer standards will be crucial for future comprehensive MS/MS validation.

### 3.7 Combination of the Different Analytical Data Sets

To establish a direct, statistically supported link between lignin structural motifs and characteristic MS/MS fragmentation behavior, the MS/MS data (relative intensities of characteristic product ions) were combined with the quantitative ^13^C NMR segment integrals (also see Table S4) using heterospectroscopic correlation analysis.^67^ This chemometric concept, previously introduced for lignin-related multianalytical data integration,^25^ enables the identification of covarying structural features across fundamentally different analytical domains by calculating correlation coefficients Φ for all parameter pairs. Here, heterospectroscopic correlation was applied to correlate MS/MS product ion pathways (neutral losses) with NMR chemical shift regions assigned to distinct lignin substructures, thereby providing a molecularly interpretable bridge between fragmentation pathways and structural features. Nonetheless, due to the relatively low number of fractions analyzed (*n* = 7), these observed trends should be interpreted cautiously as qualitative structural correlations rather than definitive quantitative predictive models.

The correlation heatmaps derived from combining MS/MS data of all isolated precursor ions (*n* = 42 per ion mode) with quantitative ^13^C NMR intervals are shown in Figure 11 for ESI-MS/MS vs ^13^C NMR and in Figure 12 for GALDI-MS/MS vs ^13^C NMR. Overall, both correlation matrices display chemically plausible and highly structured correlation patterns, demonstrating that the observed product ions are not randomly distributed but reflect systematic structural variation among the solvent fractions.

**Figure 11:**
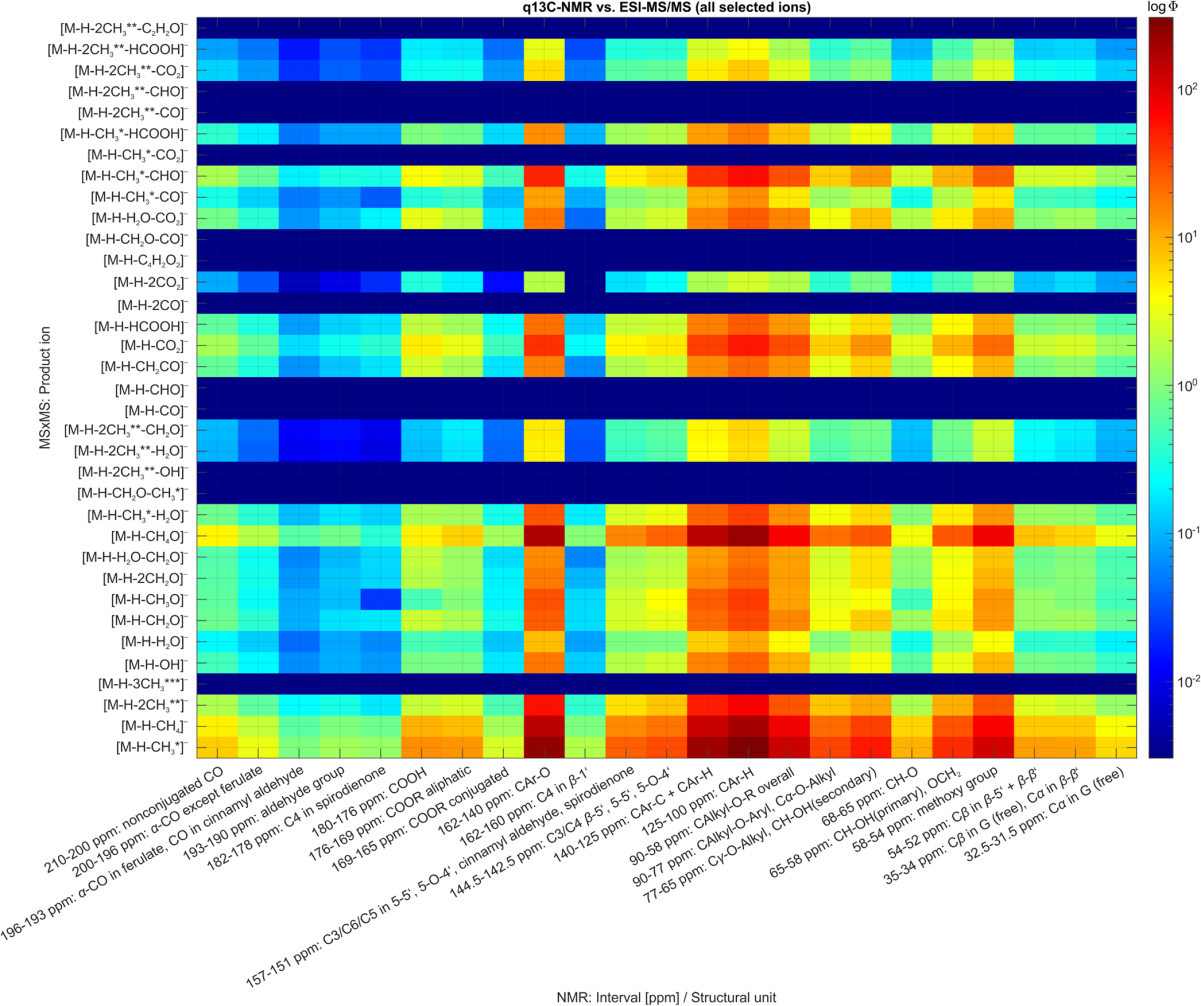
Correlation heatmap as a result of the heterospectral combination of quantitative ^13^C NMR segments and ESI-MS/MS data sets for all isolated precursor ions (total sum: 42). The calculated correlation coefficients^67^ Φ are presented logarithmic and color-coded.

**Figure 12:**
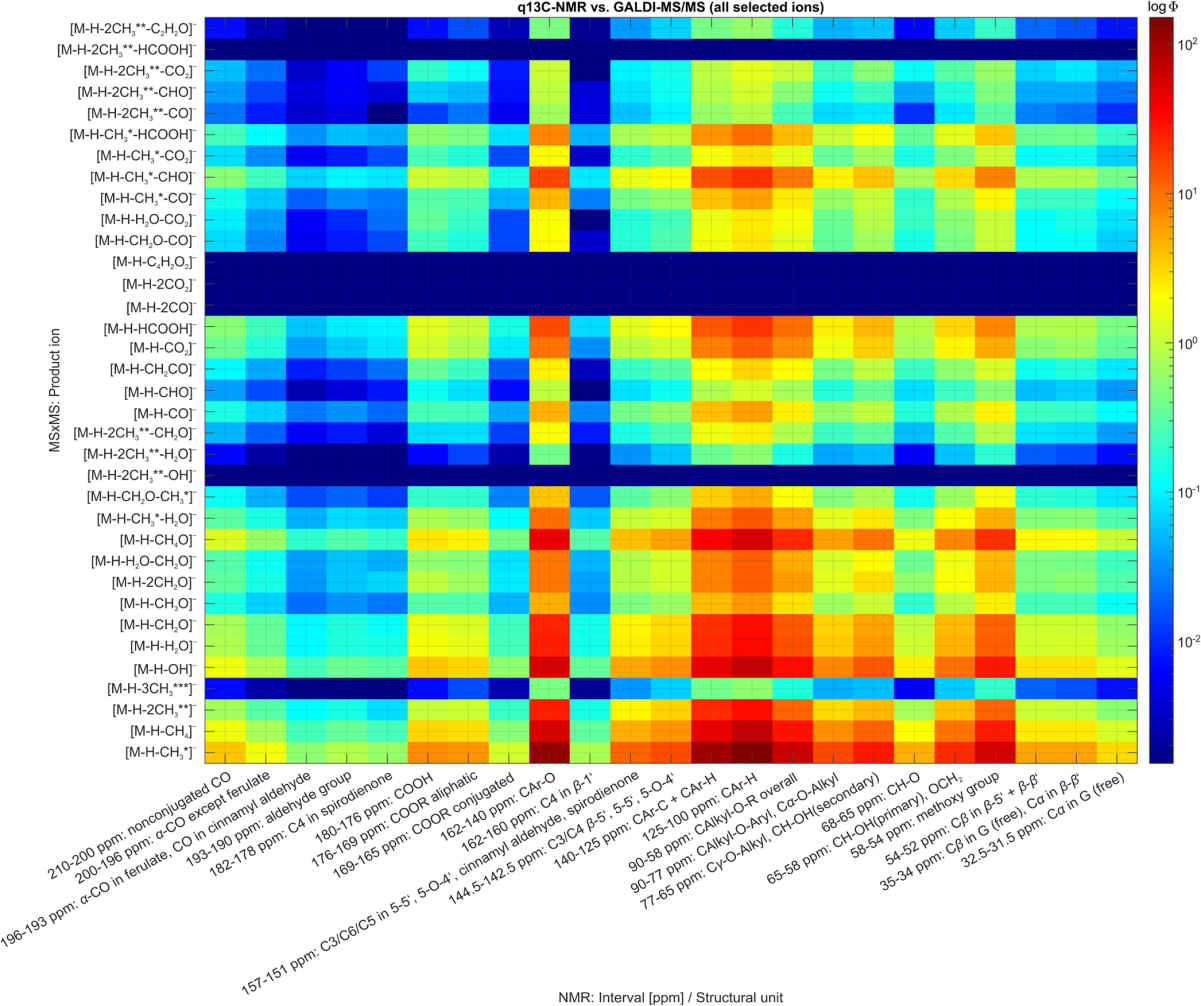
Correlation heatmap as a result of the heterospectral combination of quantitative ^13^C NMR segments and GALDI-MS/MS data sets for all isolated precursor ions (total sum: 42). The calculated correlation coefficients^67^ Φ are presented logarithmic and color-coded.

A prominent correlation block was found between methoxy-related NMR regions (notably the methoxy group signal at approximately 56–54 ppm and aromatic C_Ar_-O environments at approximately 157–151 ppm) and the product ions [M–H–CH_3_*]^−^ as well as [M–H–2CH_3_**]^−^. This confirms that methyl-loss-driven MS/MS pathways are directly connected to methoxylated G-type lignin units, consistent with the G-dominated spruce origin and the structural assignment derived from NMR. In addition, product ions linked to oxygen-containing losses such as [M–H–OH]^−^, [M–H–H_2_O]^−^, and [M–H–CH_4_O]^−^ show enhanced correlations with aromatic and side-chain NMR regions associated with oxygenated environments, supporting that hydroxyl- and methoxy-containing motifs are key determinants of fragmentation behavior in these kraft lignin fractions.

A second, highly relevant, correlation block links carboxyl-/carbonyl-related NMR signals to MS/MS losses associated with acidic functionality. In particular, [M–H–CO_2_]^−^ and [M–H–HCOOH]^−^ correlate with NMR regions assigned to nonconjugated carbonyl groups and COOH/COOR motifs. This observation supports the interpretation that carboxyl-related losses occur predominantly in precursor populations enriched in polar oxygenated substructures.

Besides these major motifs, correlation patterns are also found for NMR features assigned to aromatic carbon environments (e.g., C_Ar_-H and C_Ar_-C), confirming that the most intense MS/MS products are tightly linked to the aromatic core chemistry of lignin oligomers, rather than being exclusively determined by aliphatic side chains.

Although ESI- and GALDI-MS/MS based heatmaps share the same overall structural framework, they differ in correlation intensity and coverage. ESI-MS/MS derived correlations are generally more pronounced for fragment ions associated with highly polar oxygenated end groups (e.g., CO_2_, HCOOH, and H_2_O losses), consistent with the ionization selectivity described previously. In contrast, GALDI-MS/MS correlations show broader coverage for methyl-loss-driven pathways and aromatic structural motifs, reflecting that GALDI accesses a wider range of less polar and more condensed lignin oligomers that are underrepresented under ESI conditions. This observation further supports the conclusion that both ionization techniques probe overlapping but nonidentical lignin subspaces and therefore provide complementary structural insight.

These heterospectroscopic correlation trends were further examined using MS/MS fragmentation data of a single precursor ion that was detected in all lignin fractions by both ESI- and GALDI-FT-ICR-MS (*m*/*z* 507.17; C_28_H_28_O_9_, [M–H]^−^). The relative intensities of the corresponding product ions were also correlated with the quantitative ^13^C NMR data (see Section S16 in the SI). The resulting correlation patterns and structural trends closely matched those obtained from the heatmap analysis of all isolated ions, indicating that this precursor ion represents comparable lignin substructures. These correlations further suggest the presence of at least two G aromatic units with hydroxyl and carboxyl functionalities and, particularly in the case of GALDI-MS/MS, aldehyde groups located in close proximity to the methoxy-substituted aromatic carbons, which is also reflected in the proposed structures for this molecule in Figure S24.

To further validate and extend the heterospectroscopic interpretation beyond NMR, heterospectroscopic correlation was also applied to link MS/MS fragmentation to FT-IR spectral information. The resulting FT-IR-MS/MS correlation heat maps for all selected precursor ions are shown in Figures S27 and S28 (FT-IR vs ESI-MS/MS and FT-IR vs GALDI-MS/MS, respectively). Both correlation maps reveal that methyl, CH_2_O, and CH_4_O losses show strong correlations in spectral regions characteristic of C–H stretching (3000–2800 cm^–1^) and deformation vibrations (1470–1350 cm^–1^), whereas OH and H_2_O losses correlate strongly with the ν­(O–H) (3600–3200 cm^–1^) and ν­(C=C) (∼1600 cm^–1^) regions, indicating lignin-typical structures and a close structural relationship between methoxy and hydroxy groups. In addition, HCOOH and CO_2_ losses correlate markedly with the ν­(O–H) and ν­(C=O) (1850–1650 cm^–1^) regions, supporting their origin from carboxyl groups, while methyl and aldehyde losses occur predominantly in the C–H stretching and deformation region. The dominant correlations with ν­(C=C) suggest that fragmentation primarily takes place at the aromatic backbone rather than at interunit linkages.

In addition, the targeted FT-IR-MS/MS correlation evaluation for a representative precursor ion at *m*/*z* 507.17 (C_28_H_28_O_9_, [M–H]^−^) is provided in Figures S29 and S30. The observable correlation patterns are similar to those obtained for all ions, with distinct features mainly associated with fragment ions containing C=O-bearing functional groups. In particular, the [M–H–CO]^−^ loss shows clear correlations only in GALDI-MS/MS with ν­(O–H), ν­(C–H), ν­(C=O), and ν­(C=C) regions, indicating CO moieties derived from carbonyl, carboxyl, or ester groups located close to aromatic OH and methoxy substituents. Comparable distributions for CO_2_, HCOOH, and CHO neutral losses and the consistent presence of aldehyde-related correlations in both ESI- and GALDI-MS/MS support the assumption that water-rich fractions (W:A 100:0–80:20 (*v/v*)) are enriched in carboxyl-containing molecules, whereas acetone-rich fractions (W:A 70:30–40:60 (*v/v*)) contain more condensed and even aldehyde-rich structures (also see Figure S24).

## 4 Conclusions

This study demonstrates that UHR-MS, when applied with complementary soft ionization techniques and supported by MS/MS and chemometric correlation analysis, can capture a substantial fraction of the structural information traditionally obtained through multiple classical lignin analysis methods. Across solvent-fractionated spruce kraft lignin samples, FT-ICR-MS reproducibly resolved fraction-dependent trends in oxygenation, aromaticity, condensation degree, and heteroatom content that closely mirror conclusions drawn from FT-IR, GPC, Py-GC/MS, and quantitative NMR.

In particular, FT-ICR-MS proves to be a powerful alternative for comparative and trend-driven lignin analysis alongside classical bulk spectroscopic methods. Changes in functional group distribution, aromatic condensation, and oxygen content, typically inferred from FT-IR band ratios, Py-GC/MS product distributions, or NMR segment integrals, are directly observable at the molecular-formula level via van Krevelen analysis, heteroatom class statistics, and *n*_*C*_-*DBE* illustrations. The strong agreement between MS-derived trends and other analytical data indicates that FT-ICR-MS serves as an advanced, high-resolution complementary screening tool for lignin fractions, especially when the objective is to compare processing conditions, fractionation schemes, or feedstocks rather than to obtain absolute structural quantification.

Moreover, tandem MS substantially extends the interpretive power of FT-ICR-MS beyond elemental composition. The consistent occurrence of lignin-characteristic neutral losses and their statistically validated correlation with NMR- and FT-IR-derived structural motifs demonstrate that MS/MS fragmentation patterns encode chemically meaningful information on methoxylation, hydroxylation, carbonyl functionalities, and aromatic substitution. This enables FT-ICR-MS/MS to serve as a robust complementary approach to Py-GC/MS for functional motif identification, offering the distinct advantage of avoiding thermal degradation artifacts while preserving direct links to intact oligomeric species.

However, the study also highlights clear boundaries of UHR-MS. FT-ICR-MS does not provide direct access to interunit connectivity, linkage frequencies, or absolute molar mass distributions. Ionization selectivity and mass-dependent transmission biases lead to an underrepresentation of high-molecular-weight and weakly ionizable lignin populations, preventing FT-ICR-MS from replacing GPC for quantitative molar mass analysis or NMR for explicit linkage elucidation. Likewise, structural isomerism and three-dimensional architecture remain inaccessible without other analytical constraints.

Taken together, these findings support a shift from method accumulation to analytical reductionism in lignin characterization. FT-ICR-MS can reduce the reliance on several classical techniques by serving as a powerful, high-throughput complementary analytical platform for lignin fraction comparison, compositional mapping, and functional group trend analysis, while a reduced set of orthogonal methods, most critically GPC, Py-GC/MS, and NMR, can be selectively applied where absolute structural resolution is required. The integration of heterospectroscopic correlation further bridges the gap between molecular-level mass spectrometric data and classical structural descriptors, transforming FT-ICR-MS from a descriptive compositional tool into a mechanistically interpretable technique. While the specific structural trends and correlations established in this study are demonstrated using solvent-fractionated spruce kraft lignin samples, ongoing comparative studies further indicate that these FT-ICR-MS routines are transferable to lignins of diverse botanical origins and pulping processes.

In future studies, the development of well-defined lignin oligomer standards and curated MS/MS fragmentation libraries will be essential to further consolidate FT-ICR-MS as a quantitative and predictive tool. In addition, the availability of larger, well-defined lignin oligomer standards will provide a common structural reference framework, thereby greatly simplifying and strengthening data interpretation for complementary lignin analysis techniques such as Py-GC/MS and NMR. Within these boundaries, targeted integration of FT-ICR-MS substantially reduces the analytical burden of conventional multi-instrument workflows, enabling a faster, more information-dense, and more scalable approach to profiling complex technical lignins.

## Supplementary Material



## Data Availability

In addition, raw data, compound and integration tables, as well as correlation data for all analyzed lignin fractions underlying this study, have been deposited in Zenodo (DOI: 10.5281/zenodo.21259994).

## Notes

Overview of characteristic FT-IR bands of lignocellulosic material; H/G/S ratios of spruce kraft lignin fractions; validation data for GPC calibration; Py-GC/MS analyses of silylated lignin fractions; ^1^H NMR spectra of spruce kraft lignin fractions; quantitative ^13^C NMR and DEPT spectra of all fractions; evaluation of solid-state quantitative ^13^C NMR analyses; evaluation of liquid-state ^13^C NMR analyses; ^1^H, ^13^C-HSQC spectral data of a selected lignin fraction; M_n_, M_w_, and M_p_ values derived from FT-ICR-MS data sets and comparison of peak number, total ion current, and mean m/z of all FT-ICR-MS analyses; van Krevelen plots with statistical evaluation of all MS lignin analyses; statistical comparison of heteroatomic classes of lignin fractions; nC-DBE plots of compound classes O_9_–O_12_; correlation of MS/MS fragmentation patterns of all selected precursor ions; chemometric evaluation of the fragmentation pattern of one specific precursor ion (507.17 Da); heterospectroscopic correlation of ^13^C NMR and MS/MS data for one specific precursor ion (507.17 Da); heterospectroscopic correlation of FT-IR and MS/MS data for all lignin fractions; heterospectroscopic correlation of FT-IR and MS/MS data for one specific precursor ion (507.17 Da); supporting FIAT LignoMassStructure (PDF)

## References

[1] Nordström Y., Norberg I., Sjöholm E., Drougge R. (2013). A new softening agent
for melt spinning of softwood kraft lignin. J. Appl. Polym. Sci..

[2] Zhang R., Qi Y., Ma C., Ge J., Hu Q., Yue F.-J., Li S.-L., Volmer D. A. (2021). Characterization
of Lignin Compounds
at the Molecular Level: Mass Spectrometry Analysis and Raw Data Processing. Molecules.

[3] Abou-Dib A., Aubriet F., Hertzog J., Vernex-Loset L., Schramm S., Carré V. (2022). Next Challenges for the Comprehensive
Molecular Characterization of Complex Organic Mixtures in the Field
of Sustainable Energy. Molecules.

[4] Huang C., Xu C., Meng X., Wang L., Zhou X. (2022). Editorial: Isolation,
Modification, and Characterization of the Constituents (Cellulose,
Hemicellulose, Lignin, et al.) in Biomass and Their Bio-Based Applications. Front. Bioeng. Biotechnol..

[5] Jahn A., Hoffmann A., Blaesing L., Kunde F., Bertau M., Bremer M., Fischer S. (2020). Lignin from Annual
Plants as Raw
Material Source for Flavors and Basic Chemicals. Chem. Ing. Technol..

[6] La
Rosa J. M. D., Liebner F., Pour G., Knicker H. (2013). Partitioning
of N in growing plants, microbial biomass and soil organic matter
after amendment of N-ammonoxidized lignins. Soil Biol. Biochem..

[7] Passauer L., Fischer K., Liebner F. (2011). Preparation
and physical characterization
of strongly swellable oligo­(oxyethylene) lignin hydrogels. Holzforschung.

[8] Pecina H., Kühne G., Bernaczyk Z., Wienhaus O. (1991). Lignin-Phenol-Bindemittel
für die Holzwerkstoffherstellung. Holz
Roh-Werkst.

[9] Pospiech D., Korwitz A., Eckstein K., Komber H., Jehnichen D., Suckow M., Lederer A., Arnhold K., Göbel M., Bremer M., Hoffmann A., Fischer S., Werner A., Walther T., Brünig H., Voit B. (2019). Fiber formation and
properties of polyester/lignin blends. J. Appl.
Polym. Sci..

[10] Rosas J. M., Berenguer R., Valero-Romero M. J., Rodriguez-Mirasol J., Cordero T. (2014). Preparation of Different
Carbon Materials by Thermochemical
Conversion of Lignin. Front. Mater..

[11] Sheldon R. A. (2014). Green and
sustainable manufacture of chemicals from biomass: state of the art. Green Chem..

[12] Sharma, S. ; Kumar, A. Lignin: Biosynthesis and Transformation for Industrial Applications; Springer International Publishing, 2020. 10.1007/978-3-030-40663-9.

[13] Lupoi J. S., Singh S., Parthasarathi R., Simmons B. A., Henry R. J. (2015). Recent
innovations in analytical methods for the qualitative and quantitative
assessment of lignin. Renewable Sustainable
Energy Rev..

[14] Rojas M., Fonseca F. G., Hornung U., Funke A., Dahmen N. (2025). Synthetic
Lignin Oligomers: Analytical Techniques, Challenges, and Opportunities. ChemSuschem.

[15] Brandner D. G., Gracia Vitoria J., Kenny J. K., Bussard J. R., Jang J. H., Woodworth S. P., Vanbroekhoven K., Román-Leshkov Y., Beckham G. T. (2025). Lignin Extraction and Condensation as a Function of
Temperature, Residence Time, and Solvent System in Flow-through Reactors. ACS Sustainable Chem. Eng..

[16] Han Y., Simmons B. A., Singh S. (2023). Perspective
on oligomeric products
from lignin depolymerization: their generation, identification, and
further valorization. Ind. Chem. Mater..

[17] Capanema E.
A., Balakshin M. Y., Kadla J. F. (2004). A comprehensive approach for quantitative
lignin characterization by NMR spectroscopy. J. Agric. Food Chem..

[18] Capanema E. A., Balakshin M. Y., Kadla J. F. (2005). Quantitative characterization of
a hardwood milled wood lignin by nuclear magnetic resonance spectroscopy. J. Agric. Food Chem..

[19] Crestini C., Melone F., Sette M., Saladino R. (2011). Milled wood lignin:
a linear oligomer. Biomacromolecules.

[20] Korntner P., Sumerskii I., Bacher M., Rosenau T., Potthast A. (2015). Characterization
of technical lignins by NMR spectroscopy: optimization of functional
group analysis by 31P NMR spectroscopy. Holzforschung.

[21] Lundquist K., Aasen A. J., Daasvatn K., Forsgren B., Gustafsson J.-Å., Högberg B., Becher J. (1980). NMR Studies of Lignins.
4. Investigation of Spruce Lignin by 1H NMR Spectroscopy. Acta Chem. Scand.

[22] Xia Z., Akim L. G., Argyropoulos D. S. (2001). Quantitative
(13)C NMR analysis of
lignins with internal standards. J. Agric. Food
Chem..

[23] Zeng J., Helms G. L., Gao X., Chen S. (2013). Quantification of wheat
straw lignin structure by comprehensive NMR analysis. J. Agric. Food Chem..

[24] Del
Río J. C., Rencoret J., Prinsen P., Martínez T., Ralph J., Gutiérrez A. (2012). Structural characterization of wheat
straw lignin as revealed by analytical pyrolysis, 2D-NMR, and reductive
cleavage methods. J. Agric. Food Chem..

[25] Dütsch L., Sander K., Brendler E., Bremer M., Fischer S., Vogt C., Zuber J. (2024). Chemometric Combination of Ultrahigh
Resolving Mass Spectrometry and Nuclear Magnetic Resonance Spectroscopy
for a Structural Characterization of Lignin Compounds. ACS Omega.

[26] Karlsson M., Romson J., Elder T., Emmer Å., Lawoko M. (2023). Lignin Structure
and Reactivity in the Organosolv Process Studied by NMR Spectroscopy,
Mass Spectrometry, and Density Functional Theory. Biomacromolecules.

[27] Sette M., Wechselberger R., Crestini C. (2011). Elucidation of lignin structure by
quantitative 2D NMR. Chem. - Eur. J..

[28] Yuan T.-Q., Sun S.-N., Xu F., Sun R.-C. (2011). Characterization
of lignin structures and lignin-carbohydrate complex (LCC) linkages
by quantitative 13C and 2D HSQC NMR spectroscopy. J. Agric. Food Chem..

[29] Vuković J. P., Tišma M. (2024). The role of
NMR spectroscopy in lignocellulosic biomass
characterisation: A mini review. Food Chem.:
Mol. Sci..

[30] Sette M., Lange H., Crestini C. (2013). Quantitative
HSQC Analyses of Lignin:
A Practical Comparison. Comput. Struct. Biotechnol.
J..

[31] Talebi
Amiri M., Bertella S., Questell-Santiago Y. M., Luterbacher J. S. (2019). Establishing lignin structure-upgradeability relationships
using quantitative 1H-13C heteronuclear single quantum coherence nuclear
magnetic resonance (HSQC-NMR) spectroscopy. Chem. Sci..

[32] Romanenko I., Kurz F., Baumgarten R., Jevtovikj I., Lindner J.-P., Kundu A., Kindler A., Schunk S. A. (2022). Lignin
Depolymerization in the Presence of Base, Hydrogenation Catalysts,
and Ethanol. Catalysts.

[33] Mao J., Chen N., Cao X. (2011). Characterization
of humic substances
by advanced solid state NMR spectroscopy: Demonstration of a systematic
approach. Org. Geochem.

[34] Rönnols J., Jacobs A., Aldaeus F. (2017). Consecutive
determination of softwood
kraft lignin structure and molar mass from NMR measurements. Holzforschung.

[35] Kawamoto H. (2017). Lignin pyrolysis
reactions. J. Wood Sci..

[36] Jiang G., Nowakowski D. J., Bridgwater A. V. (2010). Effect of the Temperature on the
Composition of Lignin Pyrolysis Products. Energy
Fuels.

[37] Scholze B., Meier D. (2001). Characterization of the water-insoluble fraction from pyrolysis oil
(pyrolytic lignin). Part I. PY–GC/MS, FTIR, and functional
groups. J. Anal. Appl. Pyrolysis.

[38] Houston R. W., Elder T. J., Abdoulmoumine N. H. (2022). Investigation
into the Pyrolysis
Bond Dissociation Enthalpies (BDEs) of a Model Lignin Oligomer Using
Density Functional Theory (DFT). Energy Fuels.

[39] Hoffmann A., Bremer M., Fischer S. (2019). Determination
of Monomeric Building
Blocks by Py-GC/MS of Lignins from Agricultural Residues. Austin Biomol Open Access.

[40] Gaugler E. C., Radke W., Vogt A. P., Smith D. A. (2021). Molar mass
determination
of lignins and characterization of their polymeric structures by multi-detector
gel permeation chromatography. J Anal Sci Technol..

[41] Tolbert A., Akinosho H., Khunsupat R., Naskar A. K., Ragauskas A. J. (2014). Characterization
and analysis of the molecular weight of lignin for biorefining studies. Biofuels, Bioprod. Biorefin..

[42] Ma Q., Zhang X. (2022). An integral method
for determining the molecular composition of lignin
and its application. Sci. Rep..

[43] Papp D., Carlström G., Nylander T., Sandahl M., Turner C. (2024). A Complementary
Multitechnique Approach to Assess the Bias in Molecular Weight Determination
of Lignin by Derivatization-Free Gel Permeation Chromatography. Anal. Chem..

[44] Yang J., Sun M., Jiao L., Dai H. (2021). Molecular Weight Distribution and
Dissolution Behavior of Lignin in Alkaline Solutions. Polymers.

[45] Cui C., Zhu L., Shi Z., Zhou Z., Qi F. (2025). Guidelines for Identifying
the Structure of Heavy Phenolics in Lignin Depolymerization by using
High-Resolution Tandem Mass Spectrometry. ChemSuschem.

[46] Hertzog J., Carre V., Le Brech Y., Mackay C. L., Dufour A., Masek O., Aubriet F. (2017). Combination of electrospray ionization,
atmospheric pressure photoionization and laser desorption ionization
Fourier transform ion cyclotronic resonance mass spectrometry for
the investigation of complex mixtures - Application to the petroleomic
analysis of bio-oils. Anal. Chim. Acta.

[47] Hertzog J., Mase C., Hubert-Roux M., Afonso C., Giusti P., Barrère-Mangote C. (2021). Characterization
of Heavy Products
from Lignocellulosic Biomass Pyrolysis by Chromatography and Fourier
Transform Mass Spectrometry: A Review. Energy
Fuels.

[48] Qi Y., Fu P., Volmer D. A. (2022). Analysis
of natural organic matter via fourier transform
ion cyclotron resonance mass spectrometry: an overview of recent non-petroleum
applications. Mass Spectrom. Rev..

[49] Marshall A. G., Hendrickson C. L., Jackson G. S. (1998). Fourier transform ion cyclotron resonance
mass spectrometry: A primer. Mass Spectrom.
Rev..

[50] Nikolaev E. N., Kostyukevich Y. I., Vladimirov G. N. (2016). Fourier transform ion cyclotron resonance
(FT ICR) mass spectrometry: Theory and simulations. Mass Spectrom. Rev..

[51] Andrianova A. A., Di Prospero T., Geib C., Smoliakova I. P., Kozliak E. I., Kubátová A. (2018). Electrospray
Ionization
with High-Resolution Mass Spectrometry as a Tool for Lignomics: Lignin
Mass Spectrum Deconvolution. J. Am. Soc. Mass
Spectrom..

[52] Guthrie J. D., Rowell C. E. R., Anyaeche R. O., Alzarieni K. Z., Kenttämaa H. I. (2023). Characterization of the degradation products of lignocellulosic
biomass by using tandem mass spectrometry experiments, model compounds,
and quantum chemical calculations. Mass Spectrom.
Rev..

[53] Rizzarelli P., Leanza M., Rapisarda M. (2023). Investigations
into the characterization,
degradation, and applications of biodegradable polymers by mass spectrometry. Mass Spec. Rev..

[54] Letourneau D. R., Marzullo B. P., Alexandridou A., Barrow M. P., O’Connor P. B., Volmer D. A. (2023). Characterizing lignins
from various sources and treatment
processes after optimized sample preparation techniques and analysis
via ESI-HRMS and custom mass defect software tools. Anal. Bioanal. Chem..

[55] Letourneau D. R., Volmer D. A. (2023). Mass spectrometry-based methods for the advanced characterization
and structural analysis of lignin: A review. Mass Spectrom. Rev..

[56] Sander K., Dütsch L., Bremer M., Fischer S., Vogt C., Zuber J. (2023). Characterization of Soluble and Insoluble Lignin Oligomers by Means
of Ultrahigh Resolving Mass Spectrometry. Energy
Fuels.

[57] Morreel K., Dima O., Kim H., Lu F., Niculaes C., Vanholme R., Dauwe R., Goeminne G., Inzé D., Messens E., Ralph J., Boerjan W. (2010). Mass spectrometry-based
sequencing of lignin oligomers. Plant Physiol.

[58] Morreel K., Kim H., Lu F., Dima O., Akiyama T., Vanholme R., Niculaes C., Goeminne G., Inzé D., Messens E., Ralph J., Boerjan W. (2010). Mass spectrometry-based
fragmentation as an identification tool in lignomics. Anal. Chem..

[59] Ayala-Ortiz C., Graf-Grachet N., Freire-Zapata V., Fudyma J., Hildebrand G., AminiTabrizi R., Howard-Varona C., Corilo Y. E., Hess N., Duhaime M. B., Sullivan M. B., Tfaily M. M. (2023). MetaboDirect: an
analytical pipeline for the processing of FT-ICR MS-based metabolomic
data. Microbiome.

[60] Rivas-Ubach A., Liu Y., Bianchi T. S., Tolić N., Jansson C., Paša-Tolić L. (2018). Moving beyond
the van Krevelen Diagram: A New Stoichiometric Approach for Compound
Classification in Organisms. Anal. Chem..

[61] Dier T. K.-F., Egele K., Fossog V., Hempelmann R., Volmer D. A. (2016). Enhanced mass defect filtering to simplify and classify
complex mixtures of lignin degradation products. Anal. Chem..

[62] Pikovskoi I. I., Kosyakov D. S. (2023). Kendrick mass defect
analysis - a tool for high-resolution
Orbitrap mass spectrometry of native lignin. Anal Bioanal Chem..

[63] Pikovskoi I. I., Kosyakov D. S., Belesov A. V. (2024). Resolution-enhanced
Kendrick mass
defect analysis for improved mass spectrometry characterization of
lignin. Int. J. Biol. Macromol.

[64] Qi Y., Hempelmann R., Volmer D. A. (2016). Two-dimensional mass defect matrix
plots for mapping genealogical links in mixtures of lignin depolymerisation
products. Anal. Bioanal. Chem..

[65] Banoub J., Delmas G.-H., Joly N., Mackenzie G., Cachet N., Benjelloun-Mlayah B., Delmas M. (2015). A critique on the structural
analysis of lignins and application of novel tandem mass spectrometric
strategies to determine lignin sequencing. J.
Mass Spectrom..

[66] Kanana C., Cresry D., Pongprayoon T. (2023). Lignin Particle Production by Spray
Dryer with Self-Assembly Technique. Mater. Sci.
Forum.

[67] Noda I. (2015). Recent developments
in two-dimensional (2D) correlation spectroscopy. Chin. Chem. Lett..

[68] Zuber J., Rathsack P., Otto M. (2017). Characterization of
a pyrolysis liquid
from a German brown coal by use of negative and positive ion mode
electrospray ionization Fourier transform ion cyclotron resonance
mass spectrometry and collision-induced dissociation. Fuel.

[69] Zuber J., Dittrich N., Rathsack P., Vogt C. (2020). Direct Mass Spectrometric
Analysis of Solid Coal Samples Using Laser Desorption/Ionization Fourier
Transform Ion Cyclotron Resonance Mass Spectrometry. Energy Fuels.

[70] Sander K., Zuber J., Brendler E., Vogt C. (2025). Development of GALDI-FT-ICR-MS
Methods for the Analysis of Xylan Oligomers. Biomacromolecules.

[71] Boeriu C. G., Bravo D., Gosselink R. J., van Dam J. E. (2004). Characterisation
of structure-dependent functional properties of lignin with infrared
spectroscopy. Ind. Crops Prod.

[72] Sun X.-F., Sun R., Fowler P., Baird M. S. (2005). Extraction and characterization of
original lignin and hemicelluloses from wheat straw. J. Agric. Food Chem.

[73] Socrates, G. Infrared and Raman characteristic group frequencies:tables and charts, 3rd ed.; Wiley, 2001.

[74] Hatakeyama H., Nagasaki C., Yurugi T. (1976). Relation of certain
infrared bands
to conformational changes of cellulose and cellulose oligosaccharides. Carbohydr. Res..

[75] Schwanninger M., Rodrigues J. C., Pereira H., Hinterstoisser B. (2004). Effects of
short-time vibratory ball milling on the shape of FT-IR spectra of
wood and cellulose. Vib. Spectrosc..

[76] Xu F., Yu J., Tesso T., Dowell F., Wang D. (2013). Qualitative and quantitative
analysis of lignocellulosic biomass using infrared techniques: A mini-review. Appl. Energy.

[77] Lucarini M., Durazzo A., Kiefer J., Santini A., Lombardi-Boccia G., Souto E. B., Romani A., Lampe A., Ferrari
Nicoli S., Gabrielli P., Bevilacqua N., Campo M., Morassut M., Cecchini F. (2020). Grape Seeds: Chromatographic
Profile of Fatty Acids and Phenolic Compounds and Qualitative Analysis
by FTIR-ATR Spectroscopy. Foods.

[78] Singh R., Singh S., Trimukhe K. D., Pandare K. V., Bastawade K. B., Gokhale D. V., Varma A. J. (2005). Lignin–carbohydrate
complexes
from sugarcane bagasse: Preparation, purification, and characterization. Carbohydr. Polym..

[79] Timell, T. E. ; Lin, S. Y. ; Dence, C. W. Methods in Lignin Chemistry; Springer: Berlin Heidelberg, 1992. 10.1007/978-3-642-74065-7.

[80] Del
Río J. C., Gutiérrez A., Rodríguez I. M., Ibarra D., Martínez T. (2007). Composition of non-woody plant lignins
and cinnamic acids by Py-GC/MS, Py/TMAH and FT-IR. J. Anal. Appl. Pyrolysis.

[81] Lopes F. J. F., Silvério F. O., Baffa D. C. F., Loureiro M. E., Barbosa M. H. P. (2011). Determination
of Sugarcane Bagasse Lignin S/G/H Ratio
by Pyrolysis GC/MS. J. Wood Chem. Technol..

[82] Sequeiros A., Labidi J. (2017). Characterization and determination of the S/G ratio
via Py-GC/MS of agricultural and industrial residues. Ind. Crops Prod..

[83] Xiao L., Hu S., Han H., Ren Q., He L., Jiang L., Su S., Wang Y., Xiang J. (2021). An insight
into the OPAHs and SPAHs
formation mechanisms during alkaline lignin pyrolysis at different
temperatures. J. Anal. Appl. Pyrolysis.

[84] Masnyk M., Butkiewicz A., Górecki M., Luboradzki R., Paluch P., Potrzebowski M. J., Frelek J. (2018). In Depth Analysis of
Chiroptical Properties of Enones Derived from Abietic Acid. J. Org. Chem..

[85] SDBS National Institute of Advanced Industrial Science and Technology. SDBS-1471 - abietic acid. https://sdbs.db.aist.go.jp/CompoundLanding.aspx?sdbsno=1471 (Accessed 2026–01–20).

[86] Prothmann J., Spégel P., Sandahl M., Turner C. (2018). Identification of lignin
oligomers in Kraft lignin using ultra-high-performance liquid chromatography/high-resolution
multiple-stage tandem mass spectrometry (UHPLC/HRMSn). Anal. Bioanal. Chem..

