# Supplementary material for: Benchmarking Fourier Transform Ion Cyclotron Resonance Mass Spectrometry against Conventional Methods for the Characterization of Solvent-Fractionated Kraft Lignin
Source: Biomacromolecules. 2026 Jul 28;27(8):5766–83. doi: 10.1021/acs.biomac.6c01199 (PMC13463541; doi:10.1021/acs.biomac.6c01199)
Supplement: Supplementary file 1 [file bm6c01199_si_001.pdf]

Benchmarking Fourier Transform Ion Cyclotron Resonance Mass Spectrometry against Conventional Methods for the Characterization of Solvent-Fractionated Kraft Lignin

- SUPPORTING INFORMATION –

Wiebke Hadwich[1], Klara Sander[1], Erica Brendler[1], Martina Bremer[2], Steffen Fischer[2], Carla Vogt[1], Jan Zuber[,1]*

[1] Institute of Analytical Chemistry, TU Bergakademie Freiberg, Lessingstrasse 45, 09599 Freiberg, Germany
[2] Institute of Plant and Wood Chemistry, TU Dresden, Pienner Strasse 19, 01737 Tharandt, Germany
* Corresponding Author

Contents

S1. Additional FT-IR results and interpretation of typical bands of lignocellulosic material.......... 3

S2. H/G/S ratios of spruce kraft lignin fractions 5

S3. Validation data for GPC calibration 6

S4. Py-GC/MS analyses of silylated lignin fractions 7

S5. ^{1}H-NMR spectra of spruce kraft lignin fractions.. 8

S6. Quantitative ^{13}C-NMR and DEPT spectra of a selected spruce kraft lignin fraction 9

S7. Evaluation of all lignin-related spectral regions of the solid-state quantitative ^{13}C-NMR analyses...11

S8. Solid-state ^{13}C-NMR analyses of all spruce kraft lignin fractions 12

S9. ^{1}H, ^{13}C-HSQC spectral data of a selected lignin fraction 14

S10. Further evaluation of mass spectral data of all analyzed spruce kraft lignin fractions........17

S11. Van Krevelen plots and overall number of compound classes of the spruce kraft lignin fractions 18

S12. Statistical comparison of heteroatomic classes of analyzed lignin fractions by ESI- and GALDI-FT-ICR-MS 20

S13. n_C-DBE plots of compound classes O_9 – O_{12} for the ESI- and GALDI-FT-ICR-MS data sets 22

S14. Correlation of MS/MS fragmentation patterns of all selected precursor ions 23

S15. Chemometric evaluation of the fragmentation pattern of a specific precursor ion..............24

S16. Hetero spectroscopic correlation of ^{13}C-NMR and MS/MS data sets for a specific precursor ion...28

S17. Hetero spectroscopic correlation of FT-IR and MS/MS data sets 30

S18. Hetero spectroscopic correlation of FT-IR and MS/MS data sets for a specific precursor ion 32

S1. Additional FT-IR results and interpretation of typical bands of lignocellulosic material

Table S1: FT-IR band ratios of lignin-typical bands at 1515 cm^{-1} (aromatic skeletal vibration) and 1215 cm^{-1} (ν(C-O)) for the different analyzed spruce kraft lignin fractions.

**Fraction (W:A (*v/v*))**	**100:0**	**90:10**	**80:20**	**70:30**	**60:40**	**50:50**	**40:60**
I_{1515} / I_{1125}	0.262	0.939	1.067	1.313	1.374	1.321	1.275

Table S2: Typical wavelength regions in FT-IR spectra of lignocellulosic material. L – lignin, C – cellulose, HC – hemicellulose.

Wavelength [cm^{-1}]	Source	Vibration type and functional group	References
3660-3014	L	in-plane v(CH), aromatic v(CH), phenolic/aliphatic v(OH)	[1, 2, 3, 7]
	C/HC	aliphatic v(OH)	
2950-2930	L	out-of-plane symmetric/asymmetric v(CH)	[2, 3, 6]
	C/HC	v(CH), v(CH_2)	
2830-2850	L	symmetric/asymmetric v(CH) in OCH_3, (guaiacyl and syringyl units)	[2, 3, 6]
2500 - 2350	Proteins	N–H stretching vibration	[4]
2400	Thiols	S–H stretching vibration	[4]
2350	CO_2 (air)	C=O stretching vibrations	[4]
1700-1735	HC	v(C=O) in acetyl and carboxyl groups	[2, 3, 7]
1690-1705	L	v(C=O) ring-conjugated sinapyl aldehyde	[6, 7]
1663	L	v(C=C) in coniferyl and sinapyl alcohol, v(C=O) in coniferyl aldehyde	[2]
1650		associated H_2O and proteins	[7]
1599-1600	L	aromatic skeletal vibrations and v(C=O) in COO^-	[2, 3, 6]
1500-1510	L	aromatic skeletal vibrations	[2, 3, 6]
1450-1455	L	asymmetric δ(CH) of O-CH_3	[2, 3]
1420-1435	L	in-plane δ(CH), δ(CH_3), aromatic skeletal vibrations	[2, 3]
	C/HC	δ(CH_2)	
1415-1430	C/HC	CH_2 bending	[8]
1370	L	symmetric δ(CH) in methoxyl groups, aliphatic v(CH) in CH_3	[1, 4, 5]
	C/HC	δ(OH) and in-plane δ(CH), symmetric bending δ(CH_3) in acetyl groups	
1320-1330	L	guaiacyl and syringyl ring breathing with v(C=O)	[2, 3, 6, 7]
	C/HC	δ(OH) in-plane	[7]
1315	C	CH_2 wagging	[8]
1270	L	guaiacyl ring breathing with v(C=O)	[1, 2, 3]
1220-1230	L	aryl-O of aryl-OH and aryl-CH_3, guaiacyl/syringyl ring breathing v(CC), v(CO), v(C=O)	[2, 4]
	C/HC	asymmetric v(COC), v(CO-O) and v(C=O) in acetyl and acid groups, OH in plane	[7]
1150-1160	H/HC	v(COC) ring or aliphatic esters	[3, 7]
1140	L	δ(CH) in-plane guaiacyl	[7]
1100-1120	H/CH	v(CC) and v(CO) in mainly secondary alcohols, ring breathing, asymmetric v(COC) glycosidic	[1]
1030	L	aromatic in-plane δ(CH), δ(CO) primary alcohols	[2, 4, 7]
	C	v(CO) in C_6-OH primary alcohol, v(CC) and v(CO)	
900	C/HC	*β*-glycosidic linkages (1→4), symmetric v(COC) in-plane	[1, 2, 8]
834	L	guaiacyl-syringyl-linkage	[7]
817	L	(CH) out-of-plane guaiacyl	[7]
400-800	L	substitution on the aromatic ring	[4]
662	C	C-OH out-of plane bending	[1, 3]

S2. H/G/S ratios of spruce kraft lignin fractions

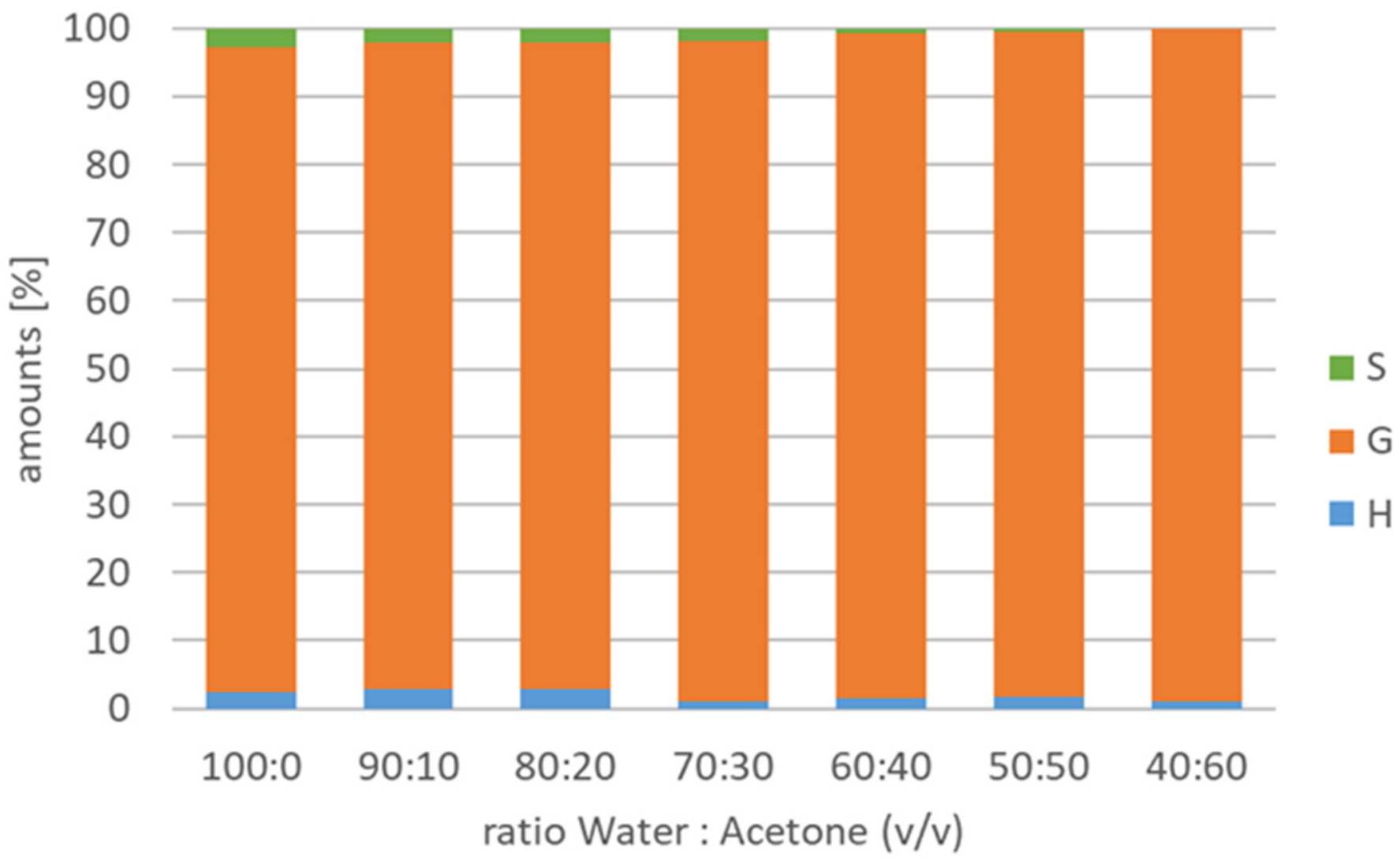

Figure S1: H/G/S lignin composition of spuce kraft lignin fractions based on Py-GC/MS results. G – guaiacyl, S – syringyl, H – p-hydroxyphenyl.

S3. Validation data for GPC calibration

Table S3: GPC estimated molecular masses for selected monomeric and dimeric lignin model substances.

**Substance**	**Molecular mass [g/mol]**	**GPC estimated molecular mass [g/mol]**
Phenol	94,11	28
2-methoxy phenol	150,18	41
2,5-dimethoxy phenol	182,17	59
Guaiacylglycerol-β-guaiacyl ether	320,34	122
Pinoresinol	358,89	131

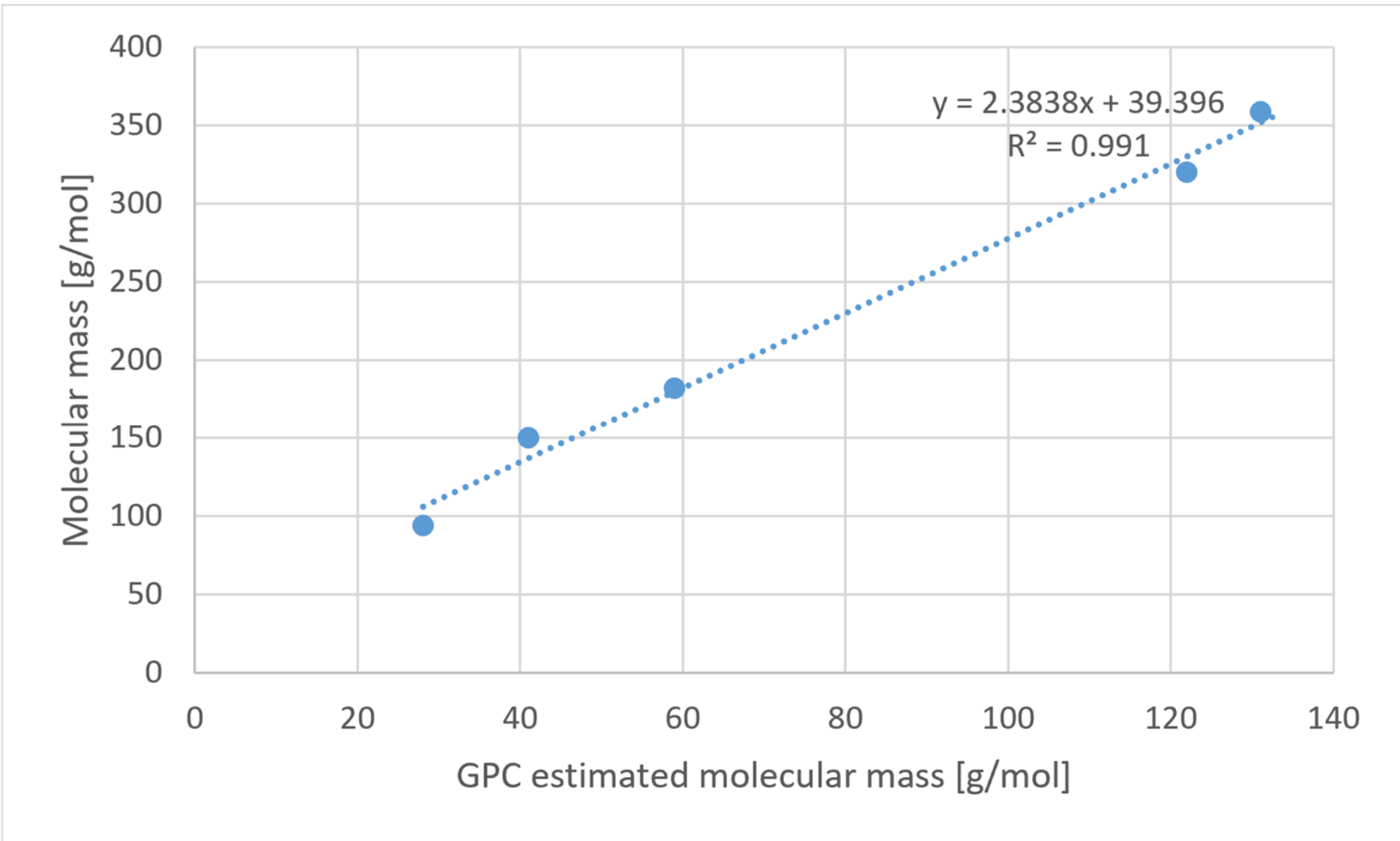

Figure S2: Linear regression of GPC estimated molecular masses and real molecular masses of selected lignin model compounds.

S4. Py-GC/MS analyses of silylated lignin fractions

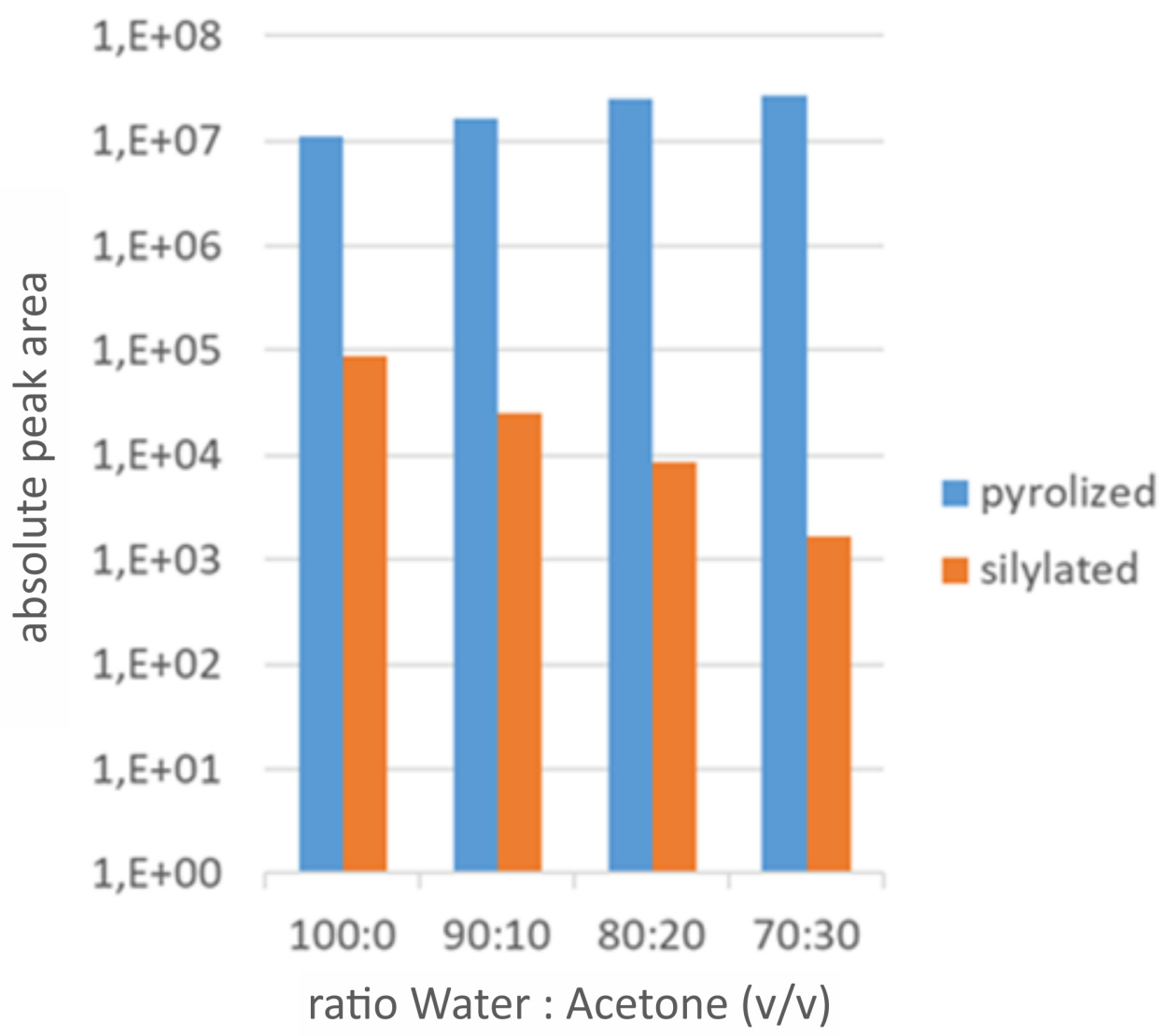

Figure S3: Comparison between peak areas resulting from Py-GC/MS and GC/MS of silylated samples standardized to 1 µg of analyzed sample.

S5. ^{1}H-NMR spectra of spruce kraft lignin fractions

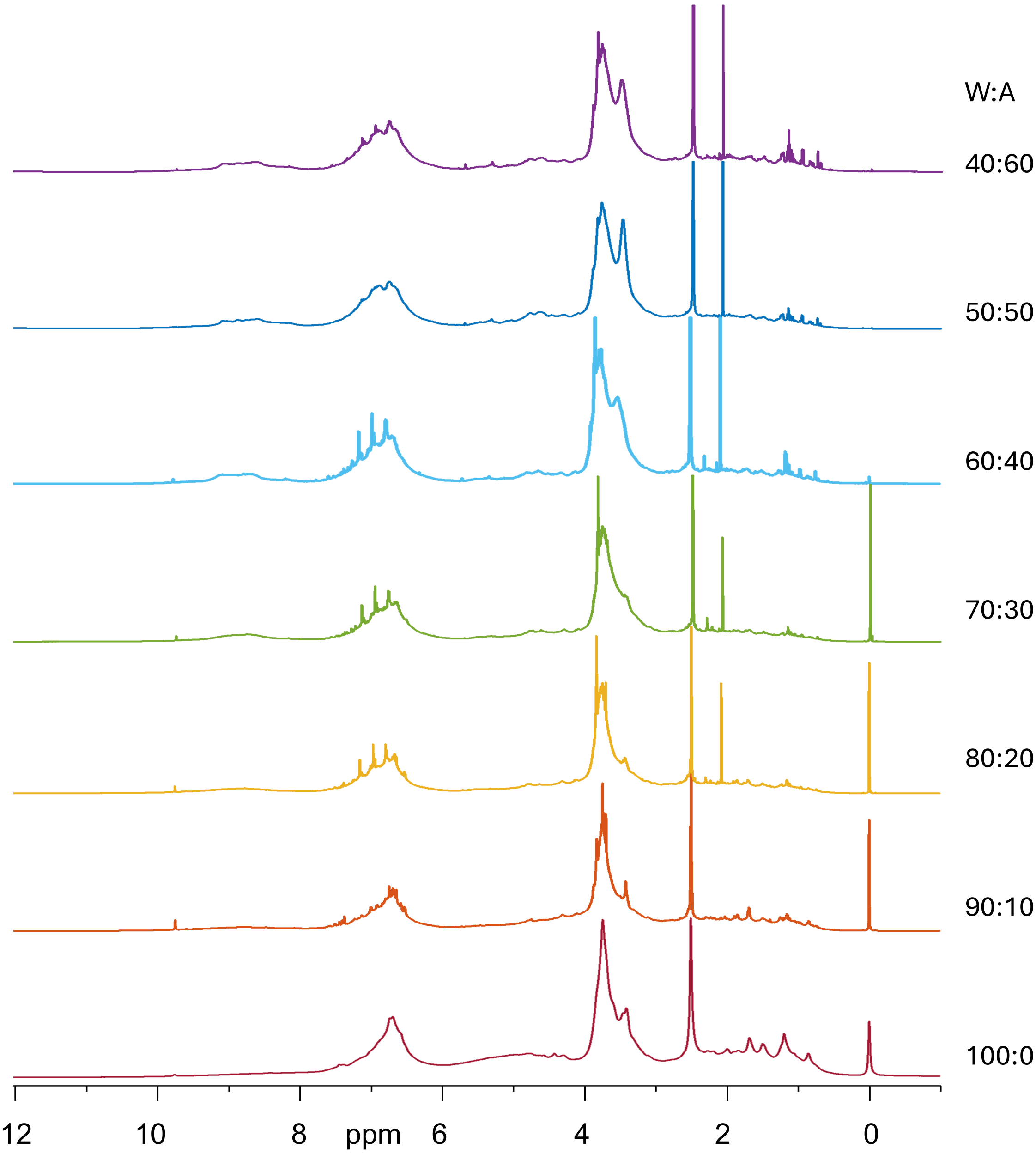

Figure S4: ^{1}H-NMR spectra of selected spruce kraft lignin fractions in DMSO-d_6. From bottom to top: W:A 100:0, 90:10, 80:20, 70:30, 60:40, 50:50, 40:60 (v/v). The signals of the NMR solvent (2.5 ppm), TMS (0 ppm) and acetone (2.08 ppm) were cut off in intensity. The signals for fraction W:A 100:0 (v/v) are broadened due to paramagnetic cations originating from the lignin.

S6. Quantitative ^{13}*C-NMR and DEPT spectra of a selected spruce kraft lignin fraction*

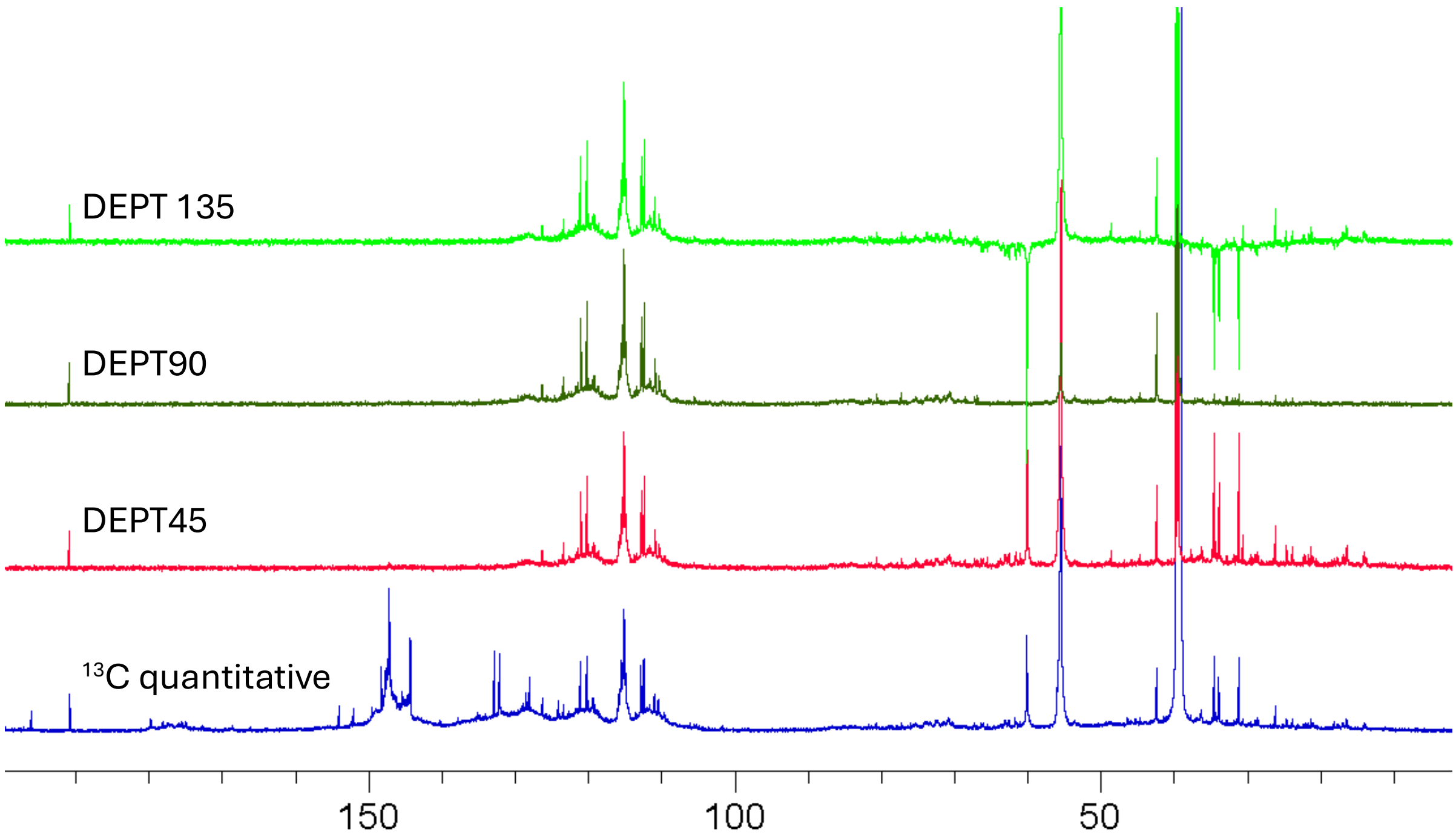

Figure S5: Quantitative ^{13}*C-NMR spectrum and DEPT spectra of spruce kraft lignin fraction W:A 90:10 (v/v). The signals of methoxy groups and DMSO-*d_6 *were cut off in intensity. The DEPT90 spectrum shows only the CH groups.*

The integral of the aromatic region (162 to 100 ppm) in the quantitative ^{13}C-NMR spectra was calibrated to a value of 6.12, corresponding to six aromatic carbons and an additional contribution of 0.12 for vinylic carbons. All other integrals were normalized to this reference value. Table S2 provides an overview of the integration regions used.

Table S4: Applied integration regions based on Capanema et al.[9] The intensity of the DMSO signal was subtracted from the alkyl region. Ar – aromatic.

Chemical shift region in ppm		Included structural elements
from	to	
210	200	non-conjugated CO
200	196	α-CO except ferulate
196	193	α-CO in ferulate, CO in cinnamyl aldehyde
193	190	aldehyde group
182	178	C_4 in spirodienone
180	176	COOH
176	169	COOR (R = aliphatic)
169	165	COOR (R = conjugated)
162	140	C_{Ar}-O
162	160	C4 in β-1‘
157	151	$C_3/C_5/C_6$ in 5-5', 5-O-4', cinnamyl aldehyde, spirodienone
144.5	142.5	C_3/C_4 in β-5', 5-5', 5-O-4'
140	125	C_{Ar}-C and C_{Ar}-H
125	100	C_{Ar}-H
90	58	C_{Alkyl}-O-R overall
90	77	C_{Alkyl}-O-Aryl, C_α-O-Alkyl
77	65	C_γ-O-Alkyl, CH-OH (secondary)
68	65	CH-O-R
65	58	CH-OH (primary), OCH_2
58	54	methoxy group (OCH_3)
54	52	C_β in β-5' + β-β'
51	1	C_{Alkyl} < 51 ppm
35	34	C_β in G (free), C_α in β-β'
32.5	31.5	C_α in G (free)

S7. Evaluation of all lignin-related spectral regions of the solid-state quantitative ^{13}C-NMR analyses

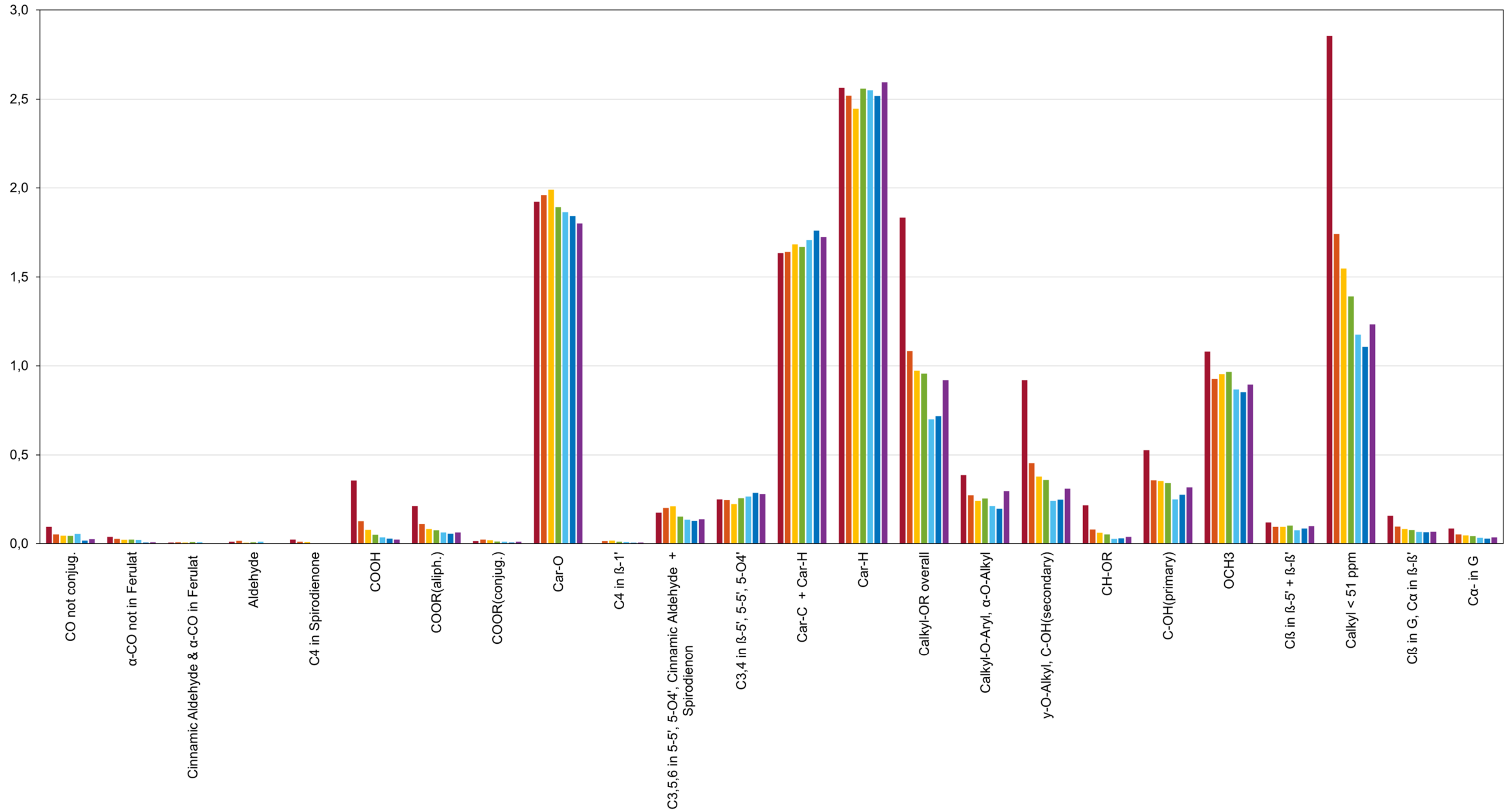

Figure S6: Distribution of structural elements within the selected spruce kraft lignin fractions according to quantitative ^{13}C-NMR, scaled to the intensity of the aromatic and vinylic carbon atoms (162 – 100 ppm, I = 6.12): W:A = 100:0, 90:10, 80:20, 70:30, 60:40, 50:50, 40:60 (v/v).

S8. Solid-state ^{13}C-NMR analyses of all spruce kraft lignin fractions

^{13}C-CP/MAS spectra of one fraction (W:A 70:30 (*v/v*)) were measured at different contact times and compared with the quantitative ^{13}C direct excitation (SP) MAS NMR spectrum of this sample. The best quantitative agreement between the CP and SP spectra was achieved with a contact time of 7 ms.

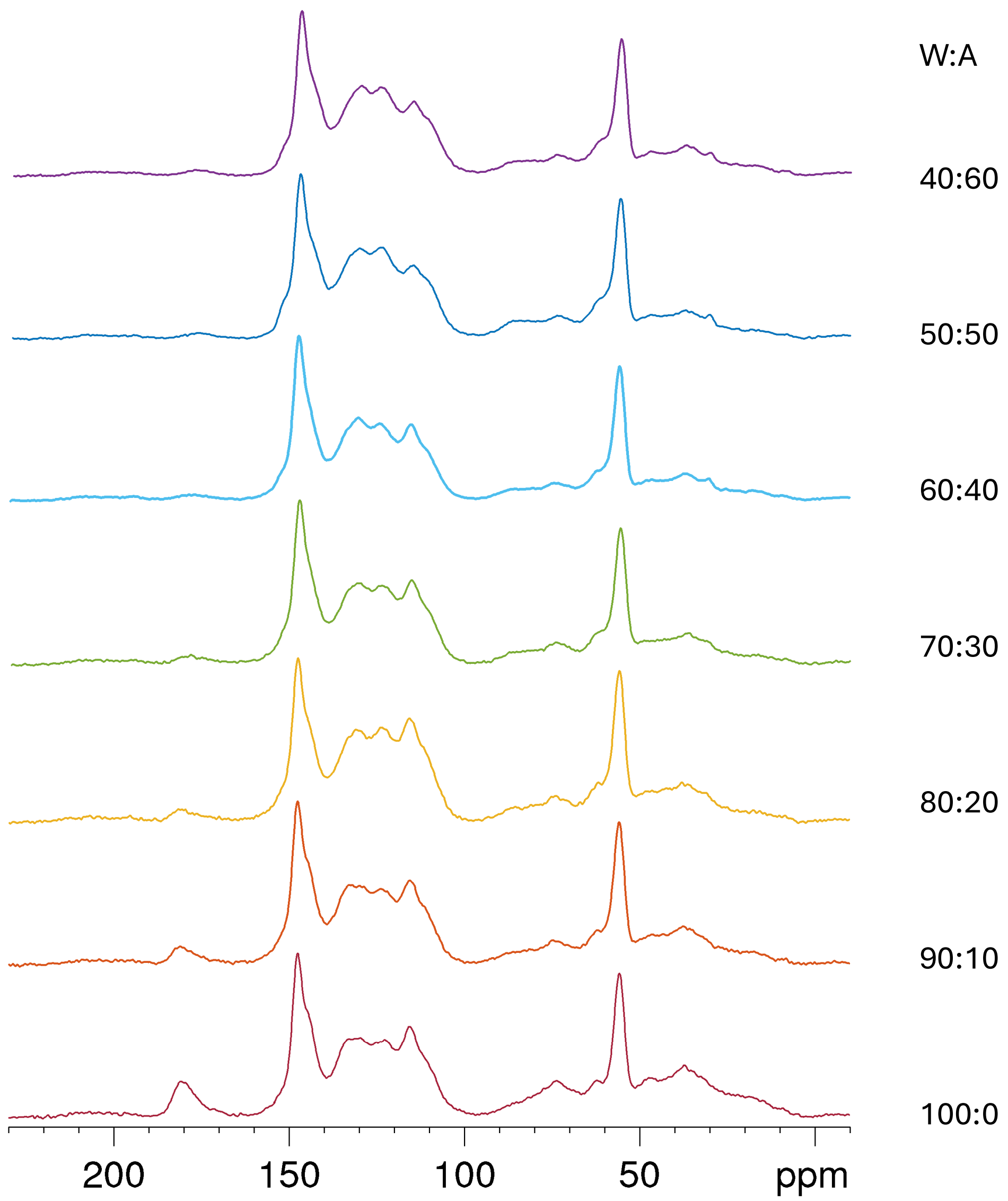

Figure S7: ^{13}C-CP/MAS spectra of selected spruce kraft lignin fractions at a contact time of 7 ms. From bottom to top: W:A 100:0, 90:10, 80:20, 70:30, 60:40, 50:50, 40:60 (v/v).

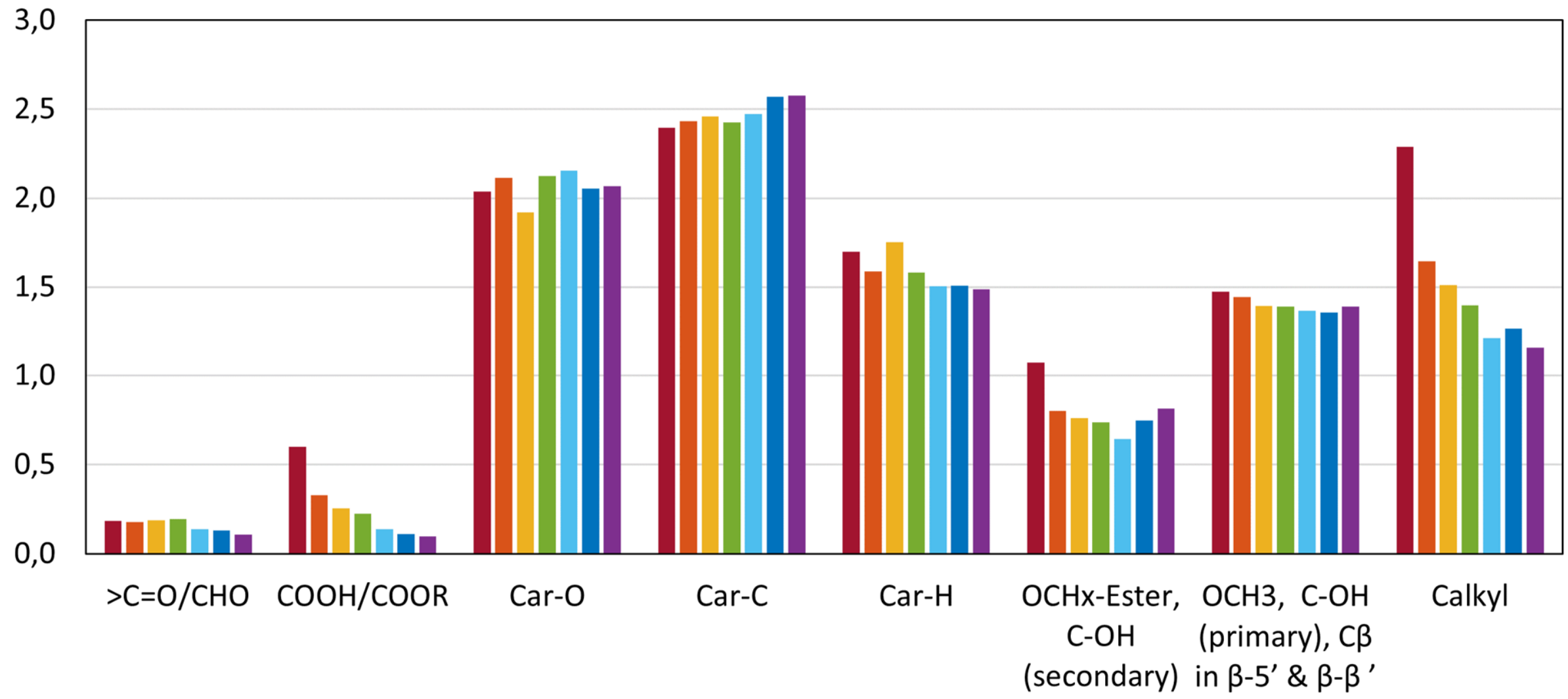

Figure S8: Distribution of structural elements within the selected spruce kraft lignin fractions according to ^{13}C-CP/MAS-NMR, scaled to the intensity of the aromatic and vinylic carbon atoms (162 – 100 ppm, I = 6.12.) W:A 100:0, 90:10, 80:20, 70:30, 60:40, 50:50, 40:60 (v/v).

S9. ^{1}H, ^{13}C-HSQC spectral data of a selected lignin fraction

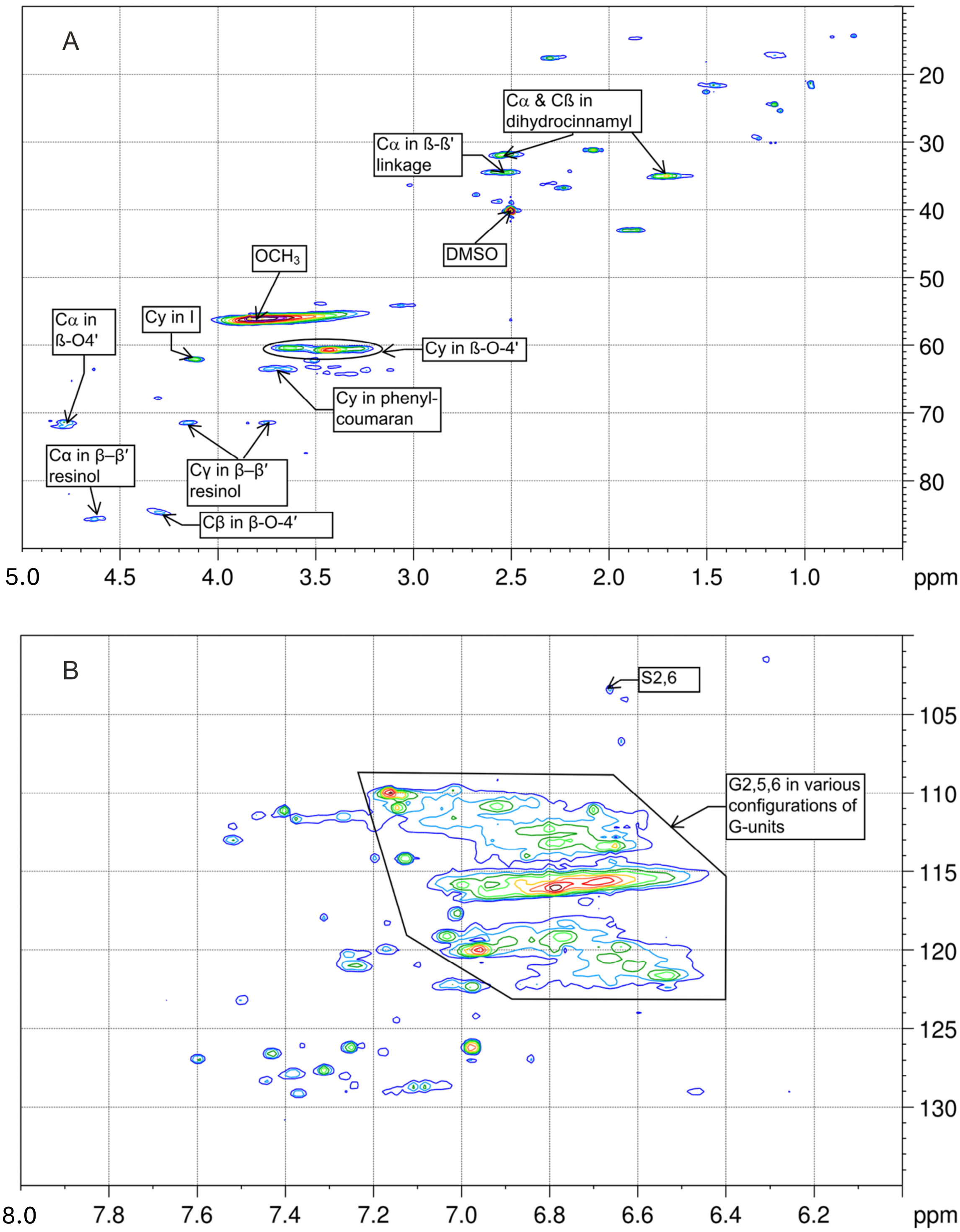

Figure S9: ^{1}H, ^{13}C HSQC spectrum of fraction W:A 70:30 (v/v) with assignments of the signals to structural groups according to [9-13]: A – aromatic part; B – aliphatic part of the spectrum.

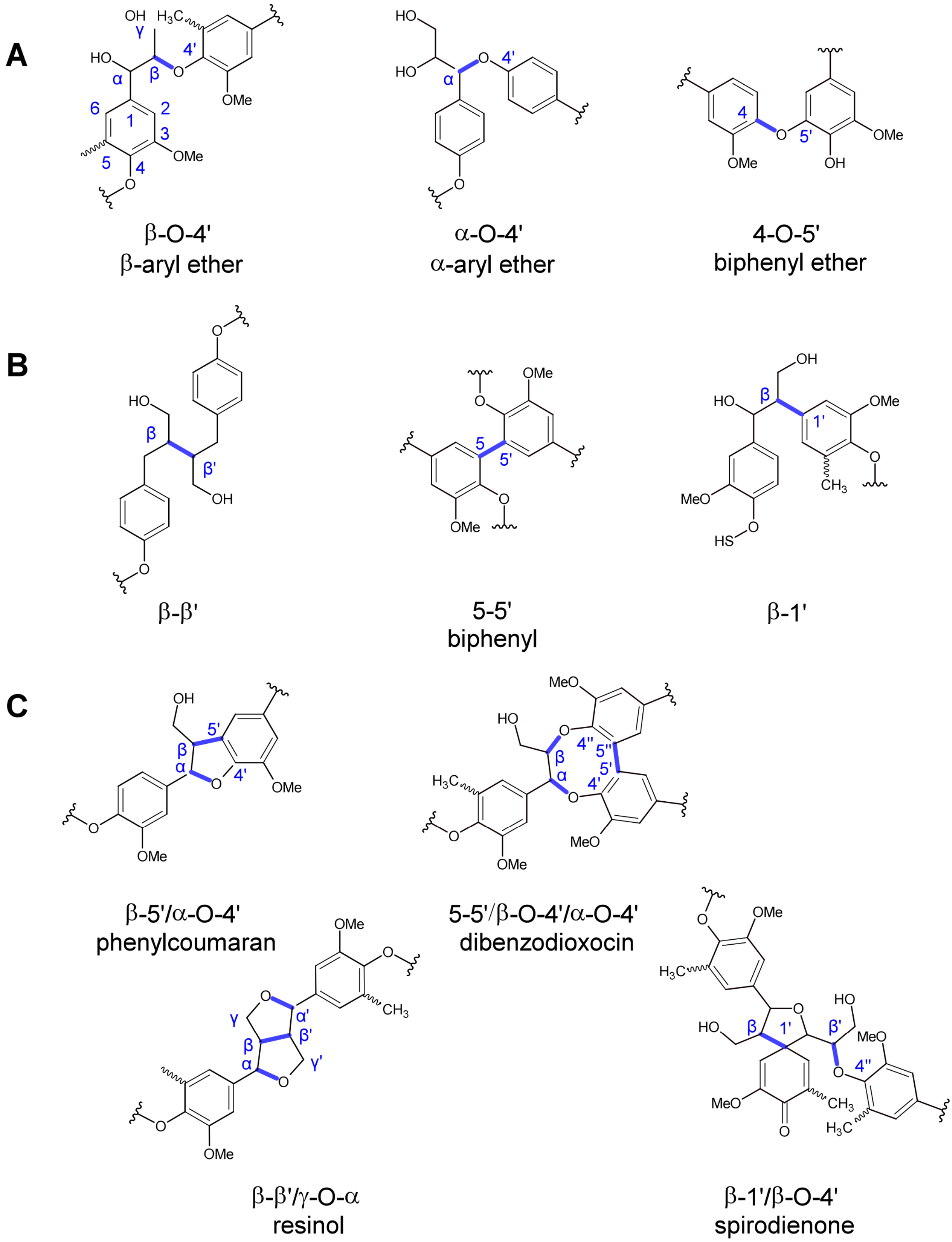

Figure S10: Linkage patterns in lignin structures: A: C-O-C bonds, B: C-C bonds, C: C-O-C and C-C bonds in cyclic structures.[14, 15]

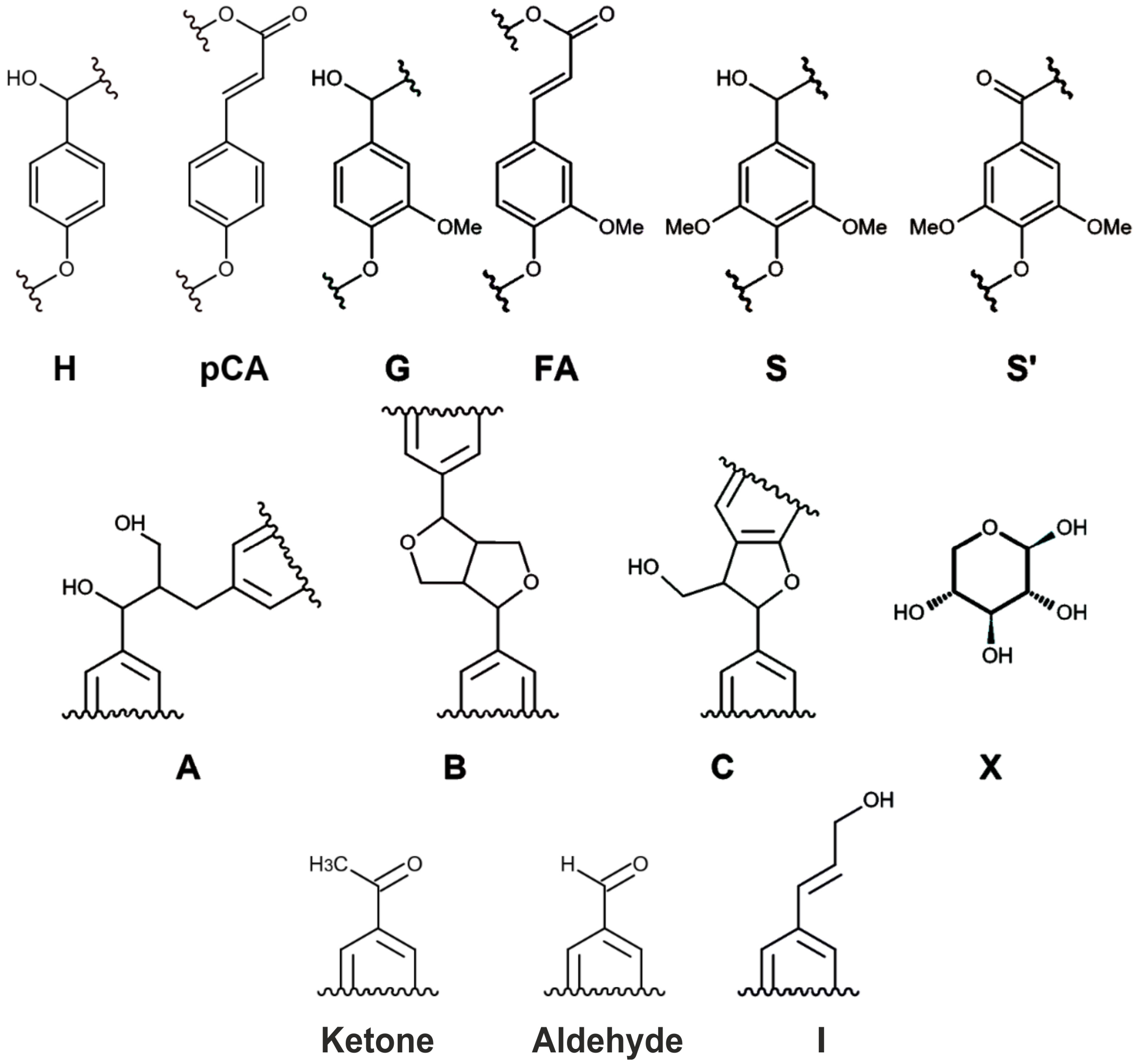

Figure S11: Important structural groups found in lignins. Upper row – aromatic units: p-hydroxyphenyl (H), p-coumarat (pCA), guaiacyl (G), ferulate (FA), syringyl (S), oxidized syringyl (S'). Middle row – sidechain units: β-O-4 aryl alkyl ethers (A), resinol (B); phenylcoumaran (C); xylane (X). Bottom row – end groups: ketone, aldehyde, cinnamyl alcohol (I).[10, 11]

S10. Further evaluation of mass spectral data of all analyzed spruce kraft lignin fractions

Table S5: FT-ICR-MS derived number average (M_n), weight average (M_w), and peak (M_p) molar masses for the solvent-fractionated spruce kraft lignin samples. Calculations are based on averaged mass spectra, considering only signals with S/N ≥ 7.

Analysis	M_n	M_w	M_p
W:A 100:0, ESI	471.335589	567.566793	361.129338
W:A 90:10, ESI	463.972447	638.914123	511.197180
W:A 80:20, ESI	507.183363	648.182629	507.165919
W:A 70:30, ESI	433.390486	658.109597	507.165939
W:A 60:40, ESI	474.379517	608.233355	507.165957
W:A 50:50, ESI	425.260781	673.041999	507.166045
W:A 40:60, ESI	431.268994	736.607500	507.166051
W:A 100:0, GALDI	499.303154	639.757546	511.197323
W:A 90:10, GALDI	477.585911	653.968773	511.197344
W:A 80:20, GALDI	479.622597	685.038175	507.166047
W:A 70:30, GALDI	489.012410	594.435078	507.166119
W:A 60:40, GALDI	551.855927	684.840800	507.166043
W:A 50:50, GALDI	498.142385	605.718912	575.171089
W:A 40:60, GALDI	447.276157	573.215231	551.171027

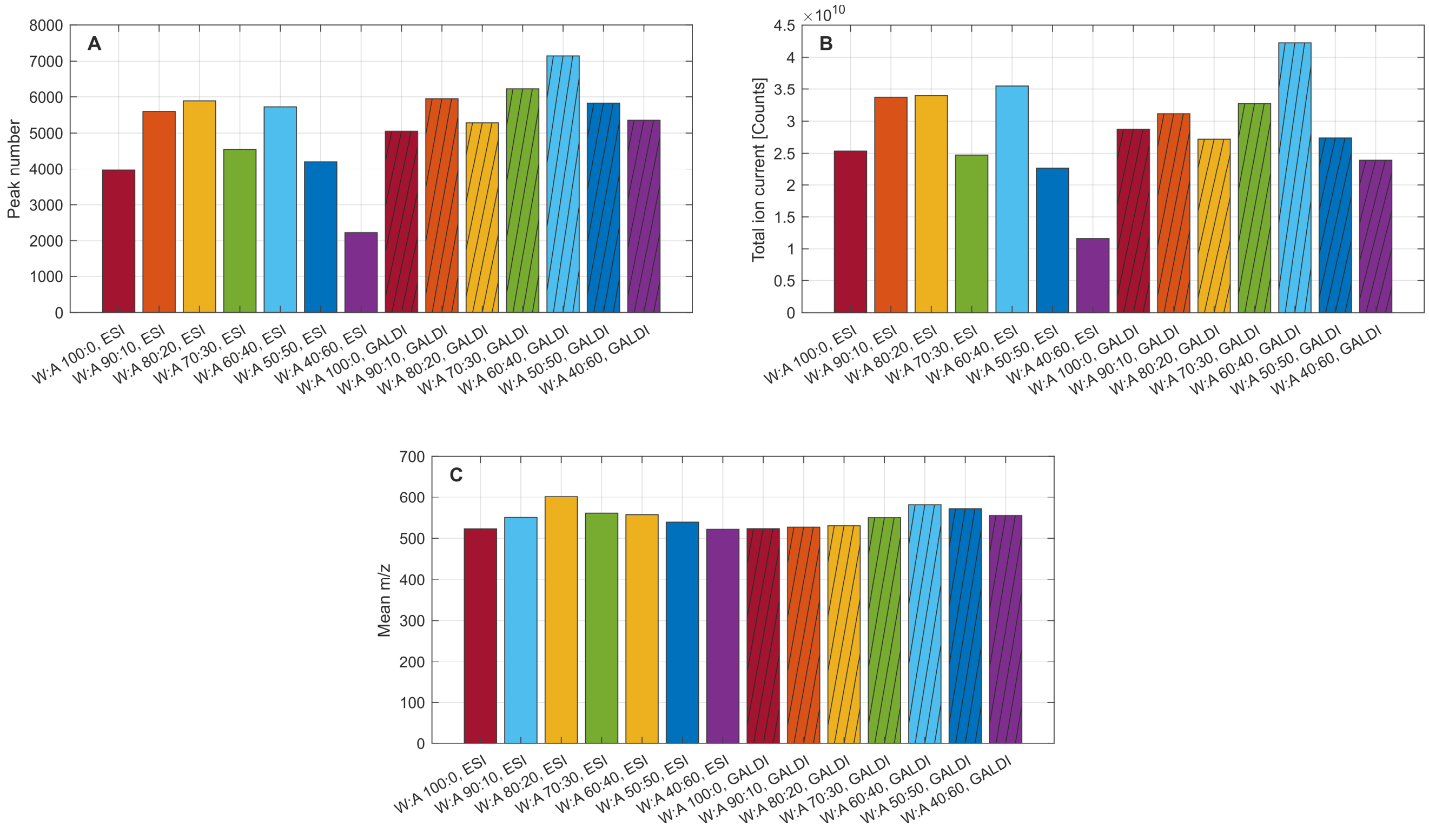

Figure S12: Comparison of peak number (A), total ion current (TIC) (B) and mean m/z (C) of the ESI- and GALDI-FT-ICR-MS analyses of the spruce kraft lignin fractions.

S11. Van Krevelen plots and overall number of compound classes of the spruce kraft lignin fractions

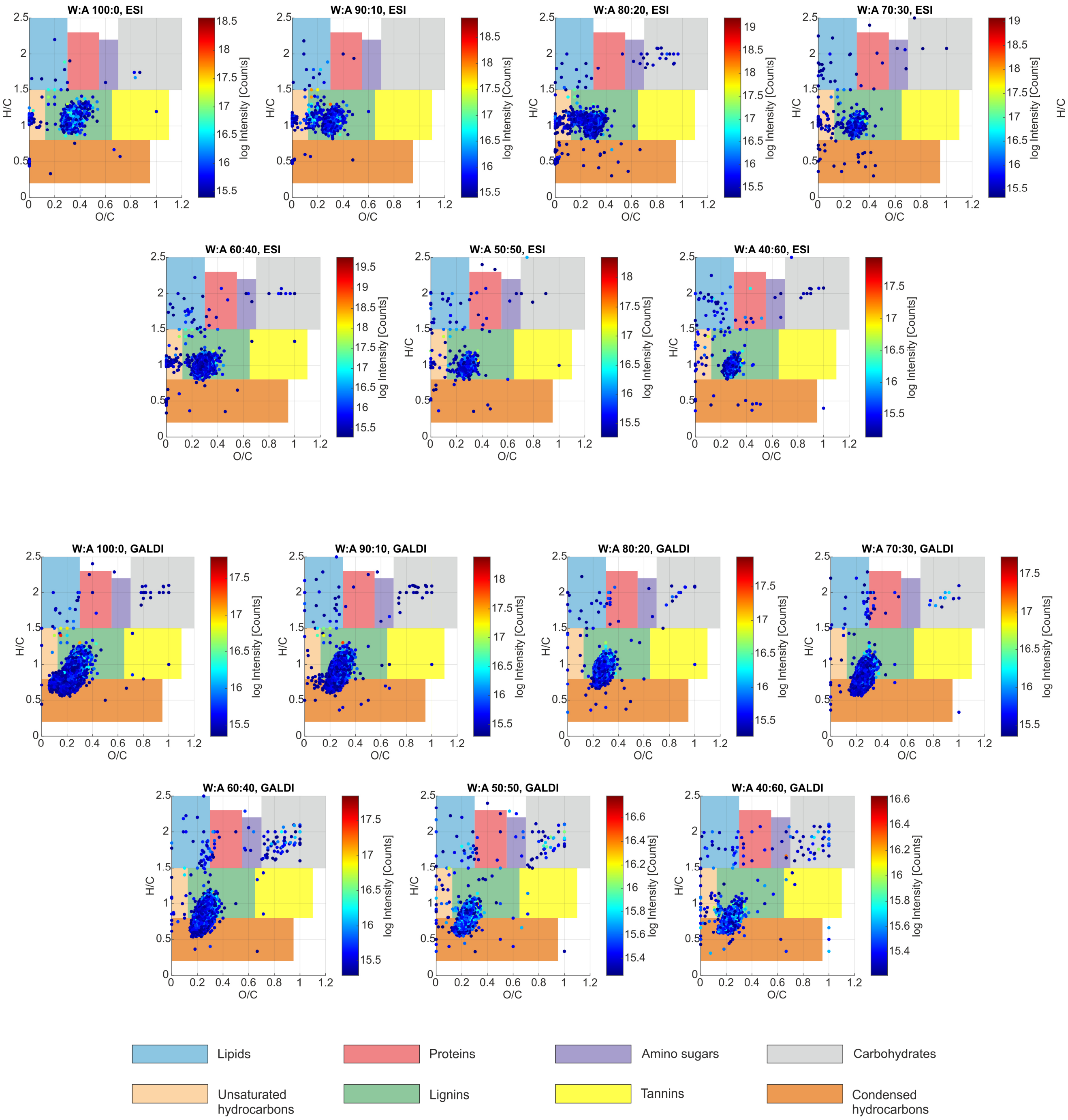

Figure S13: Van Krevelen plots generated from the FT-ICR-MS data of the different analyzed spruce kraft lignin fractions. The compound classes and their respective regions were assigned according to literature[16] and are displayed color-coded, as described at the bottom. The observed intensity is presented logarithmically and color-coded (blue: low intensity, yellow / green: medium intensity, red: high intensity, see color bar).

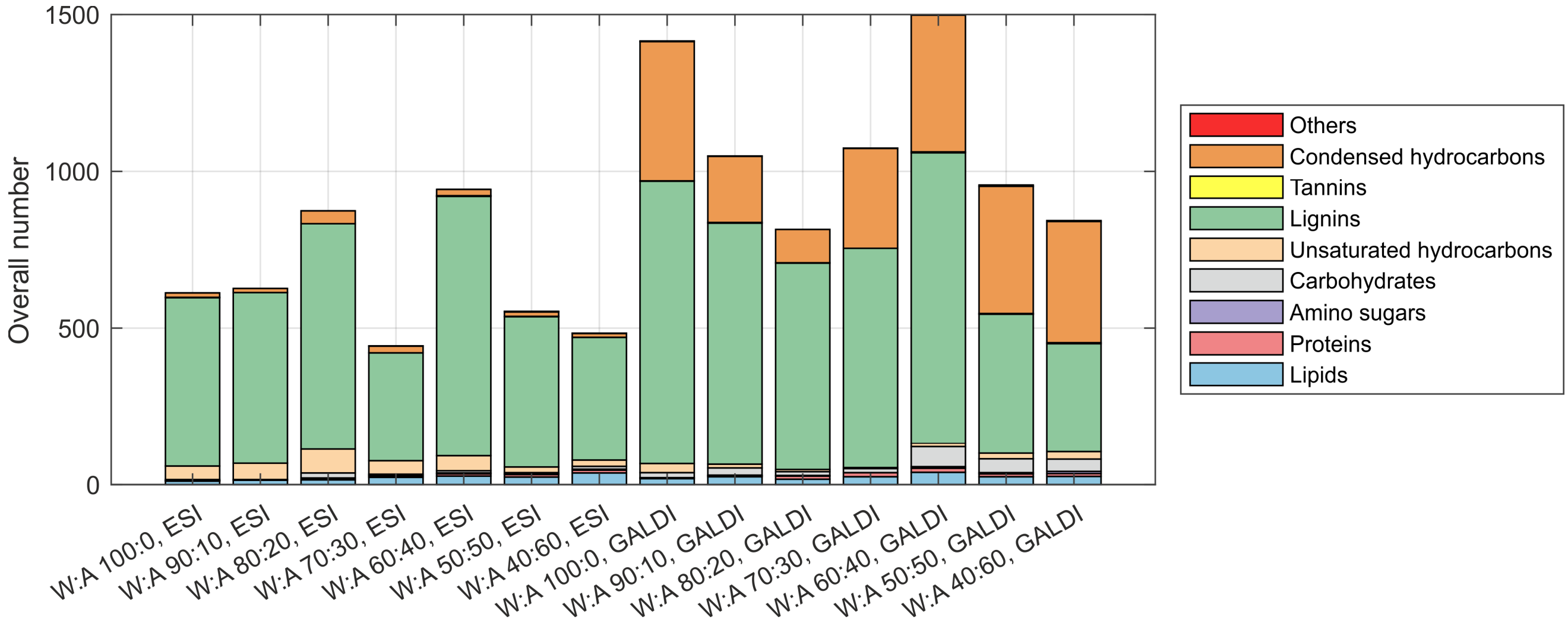

Figure S14: Overall number of molecular formulae per compound classes of the different spruce kraft lignin fractions, as calculated according to the van Krevelen plots of the FT-ICR-MS data. The compound classes and their respective regions were adapted from literature[16].

S12. Statistical comparison of heteroatomic classes of analyzed lignin fractions by ESI- and GALDI-FT-ICR-MS

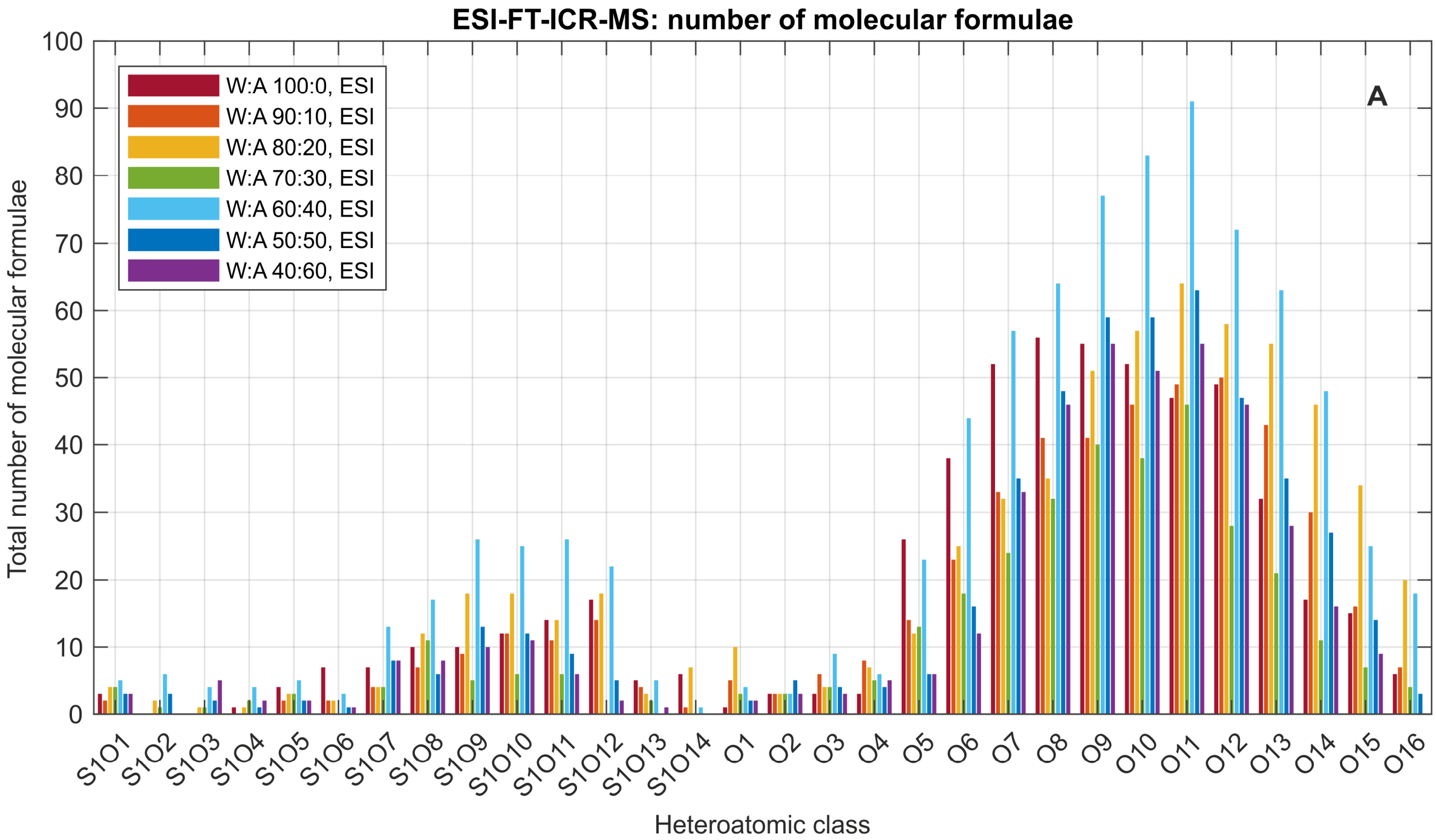

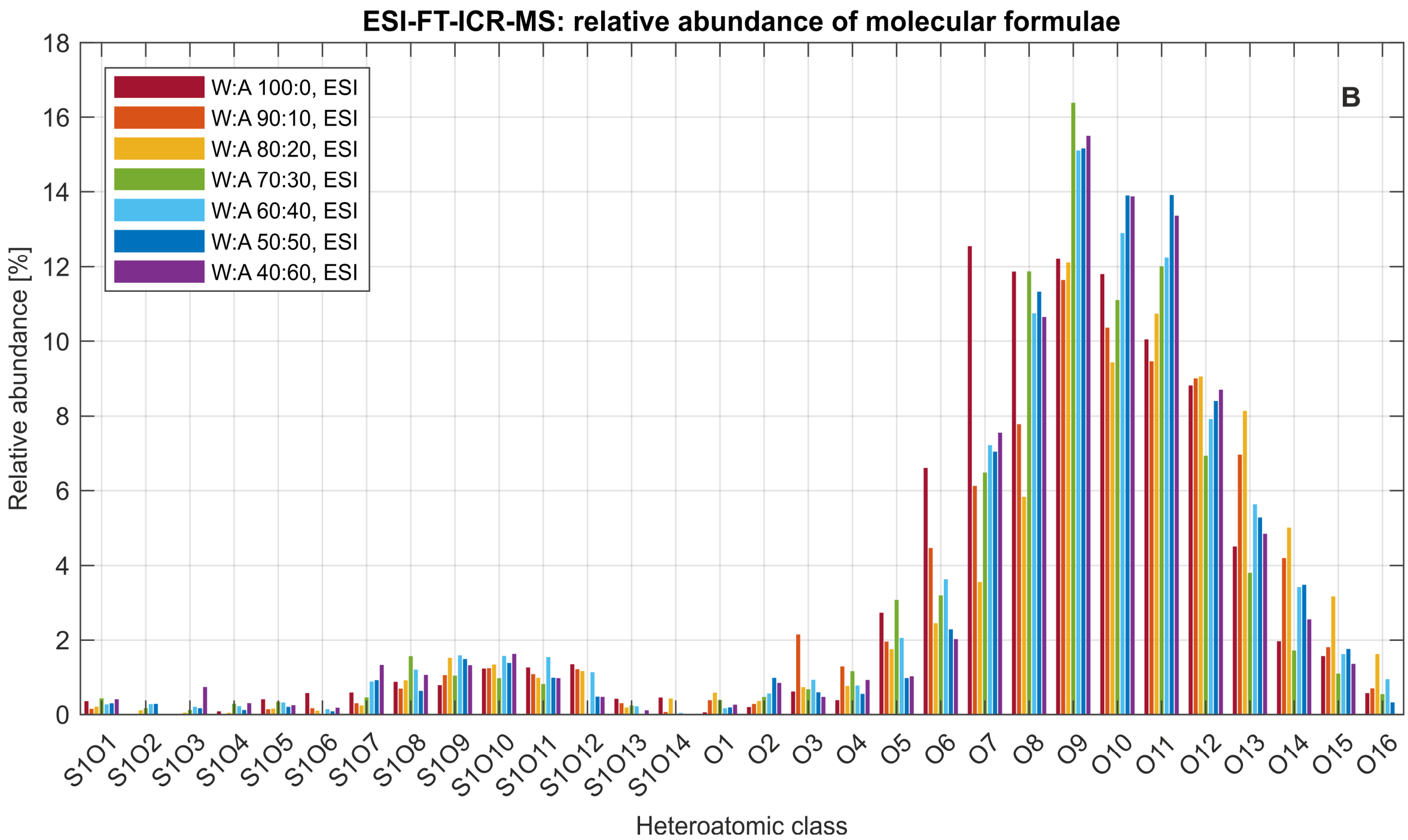

Figure S15: Statistical comparison of heteroatomic classes for all analyzed lignin fractions by ESI-FT-ICR-MS: (A) total number of assigned molecular formulae; (B) relative abundance of heteroatomic classes.

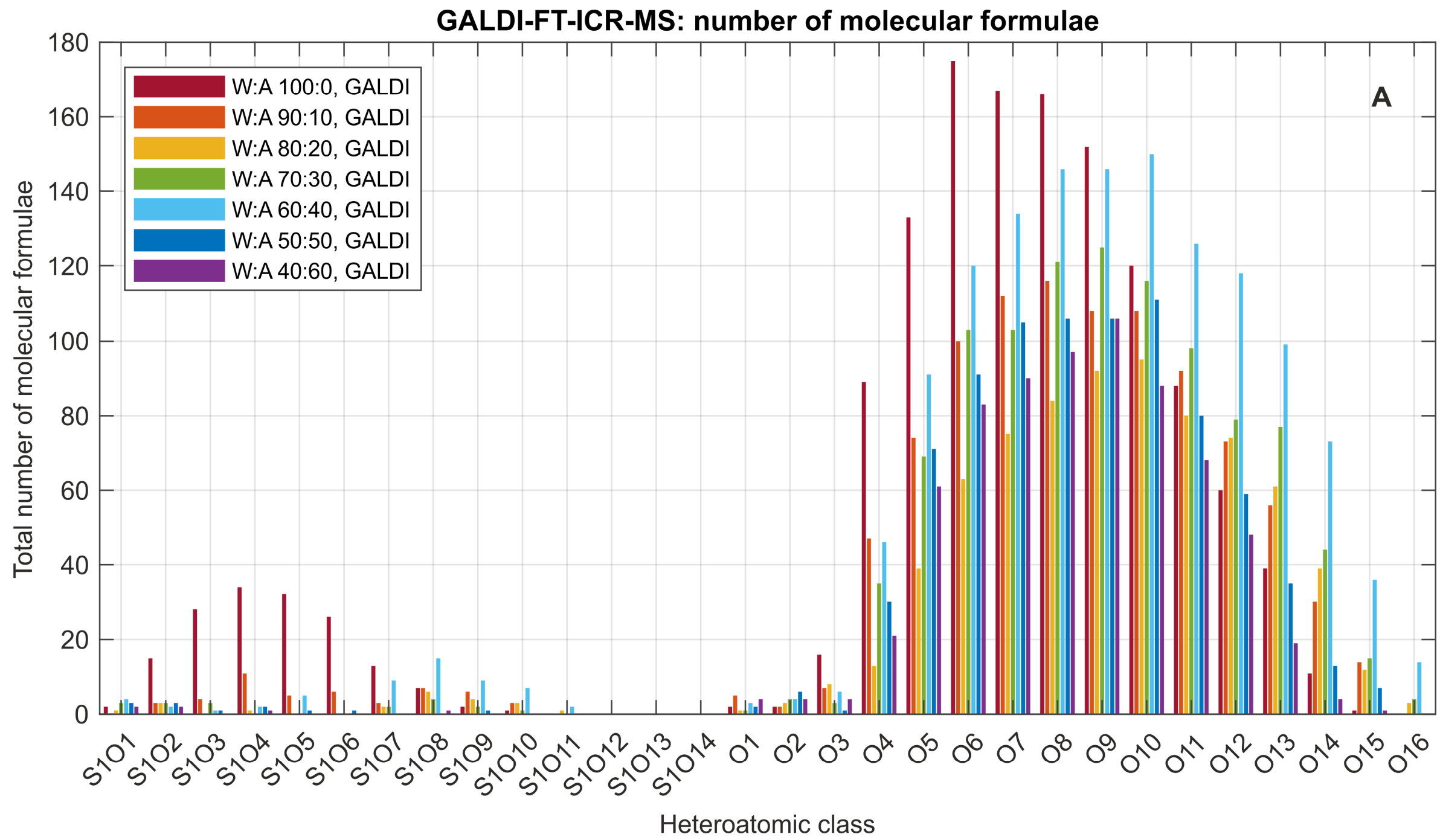

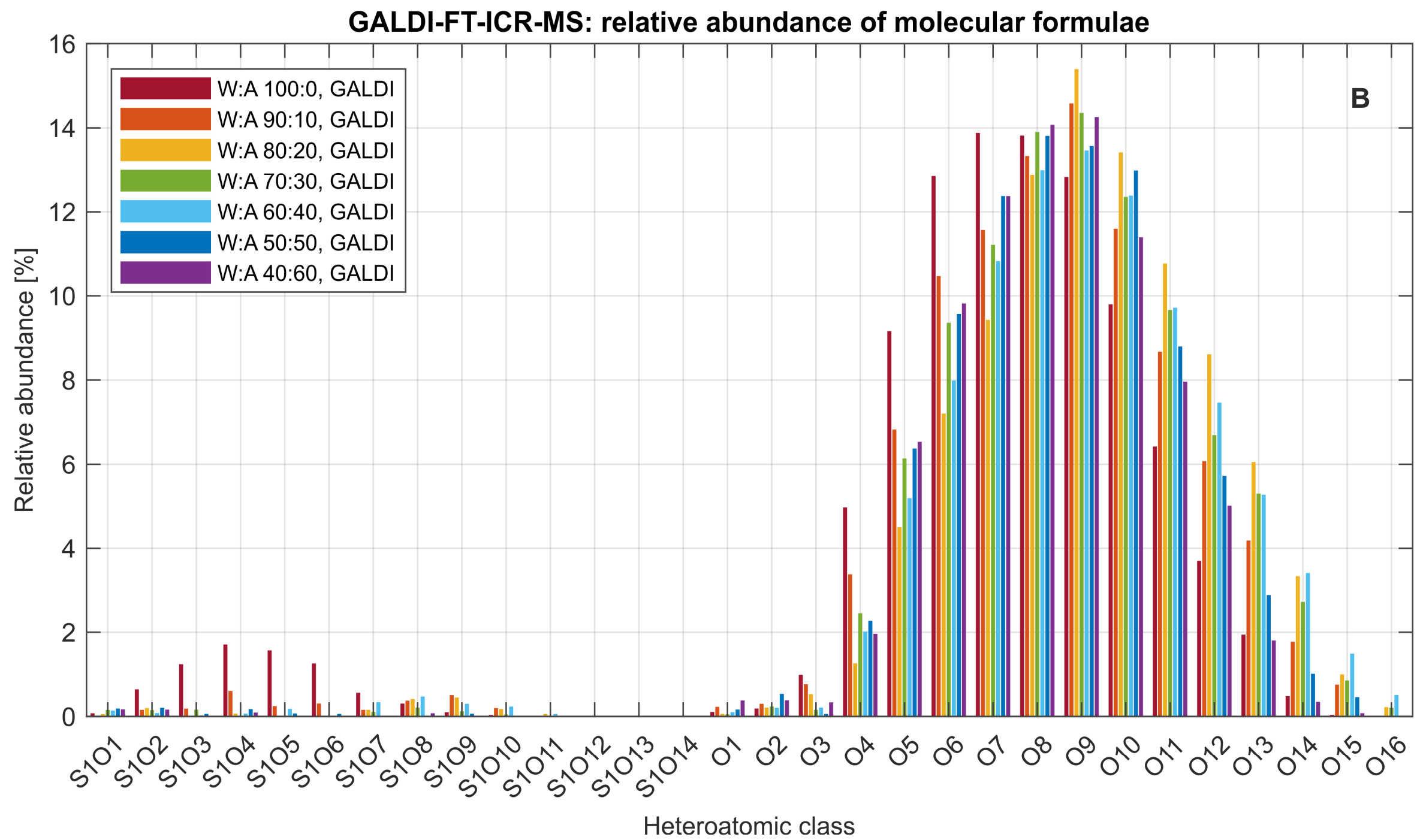

Figure S16: Statistical comparison of heteroatomic classes for all analyzed lignin fractions by GALDI-FT-ICR-MS: (A) total number of assigned molecular formulae; (B) relative abundance of heteroatomic classes.

S13. n_C-DBE plots of compound classes O_9 – O_{12} for the ESI- and GALDI-FT-ICR-MS data sets

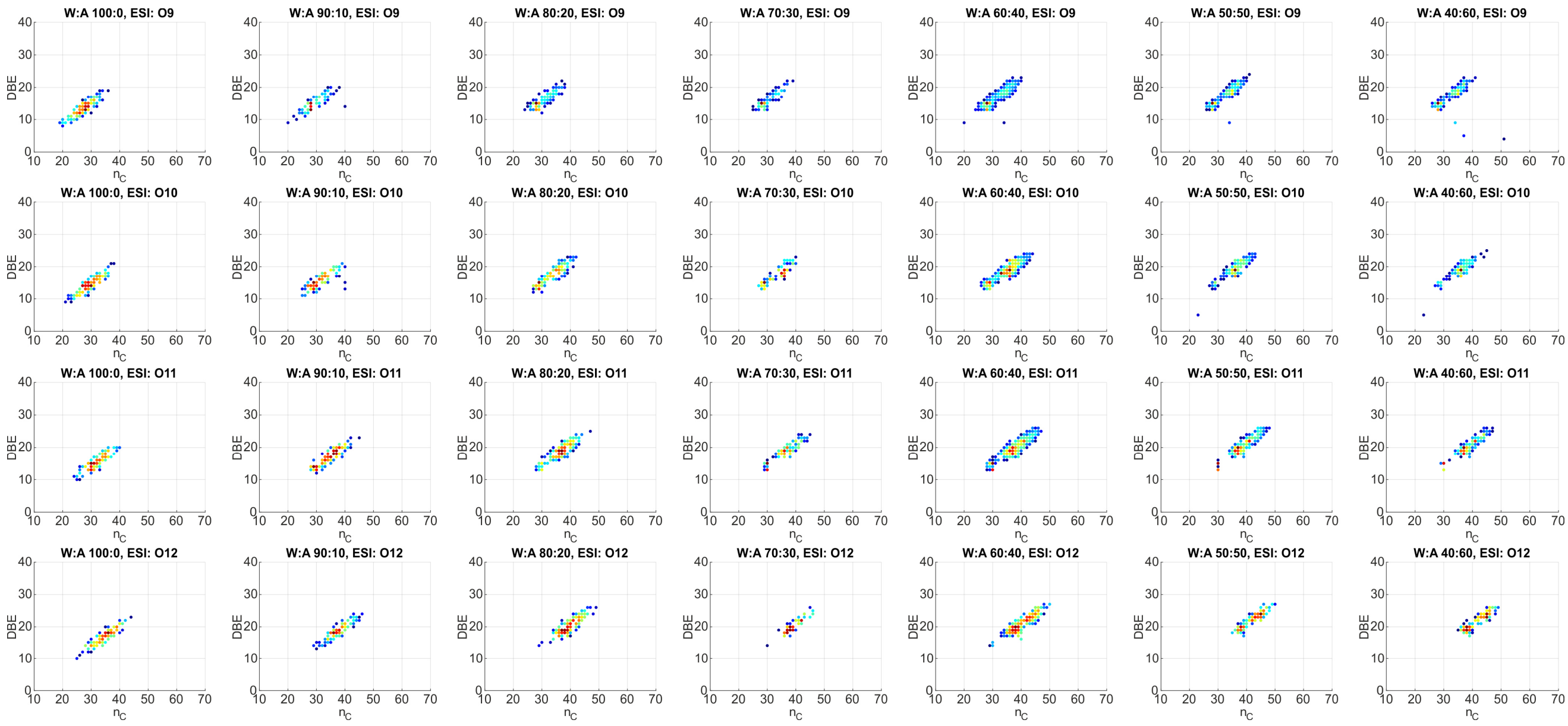

Figure S17: n_C-DBE plots of heteroatomic classes O_9 - O_{12} for the analyzed spruce kraft lignin fractions by ESI-FT-ICR-MS. The observed intensity is presented logarithmic and color-coded (blue: low intensity, yellow / green: medium intensity, red: high intensity).

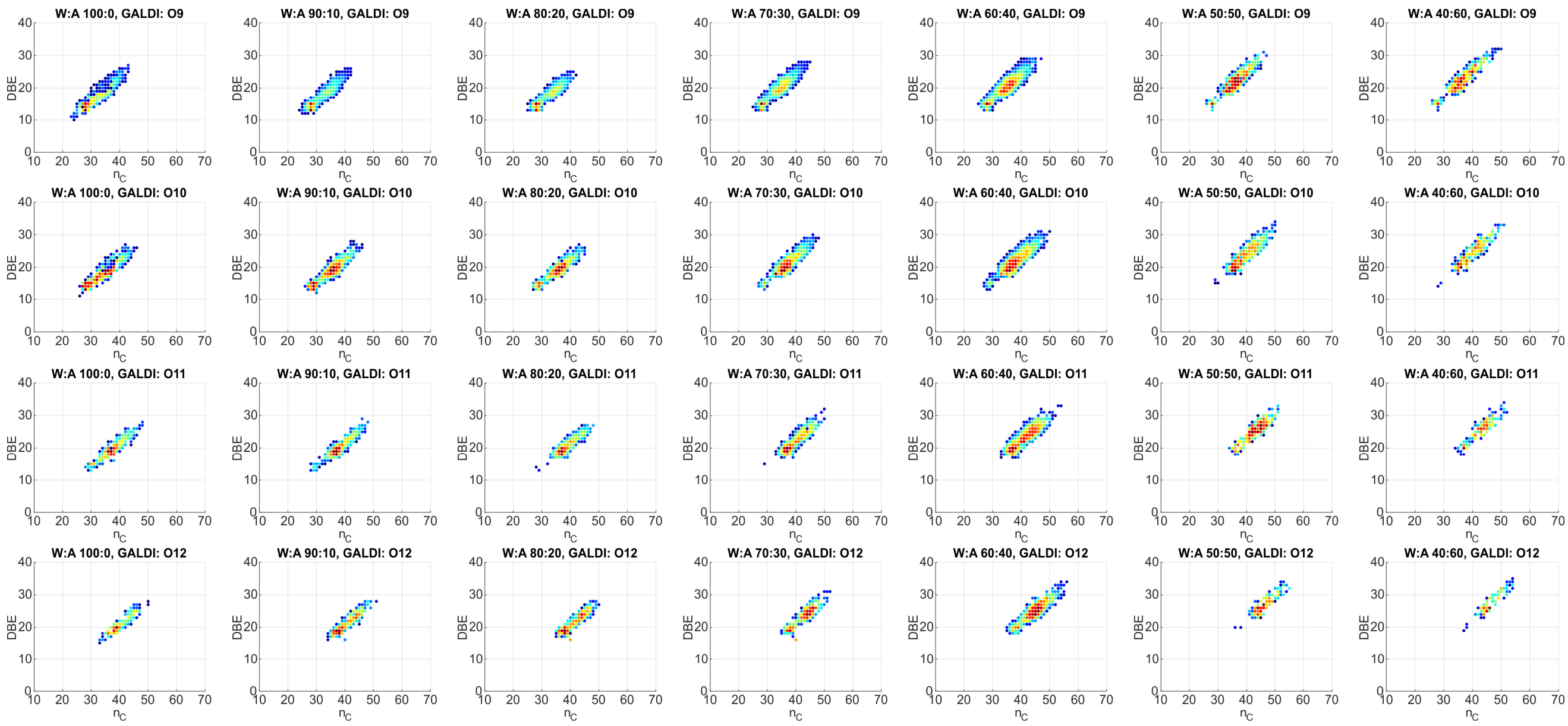

Figure S18: n_C-DBE plots of heteroatomic classes O_9 - O_{12} for the analyzed spruce kraft lignin fractions by GALDI-FT-ICR-MS. The observed intensity is presented logarithmic and color-coded (blue: low intensity, yellow / green: medium intensity, red: high intensity).

S14. Correlation of MS/MS fragmentation patterns of all selected precursor ions

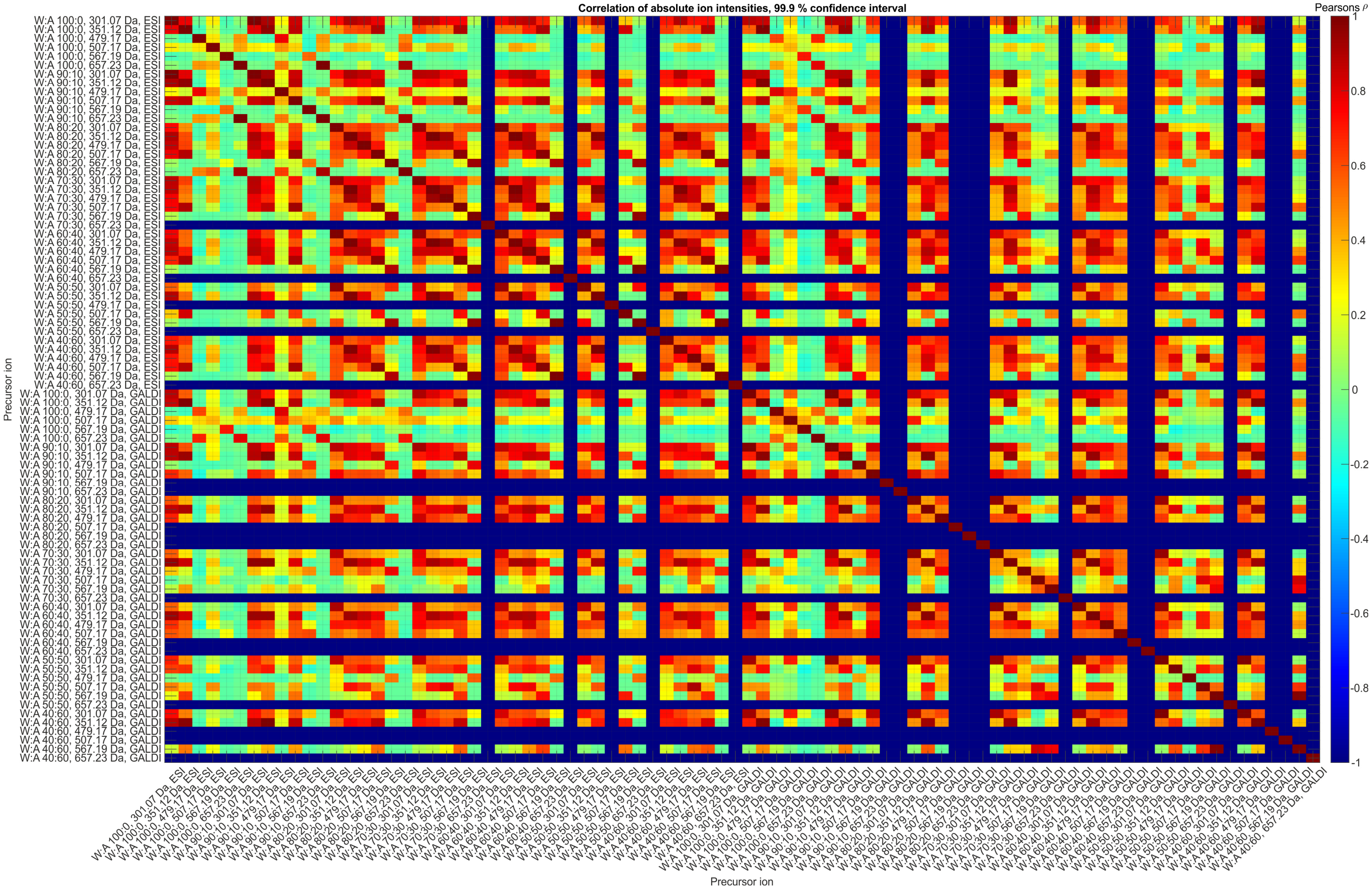

Figure S19: Correlation heatmap of the ESI-MS/MS and GALDI-MS/MS analyses of 84 precursor ions for all analyzed lignin fractions. For this chemometric evaluation the absolute intensity information and a confidence interval of 99.9 % were used.

S15. Chemometric evaluation of the fragmentation pattern of a specific precursor ion

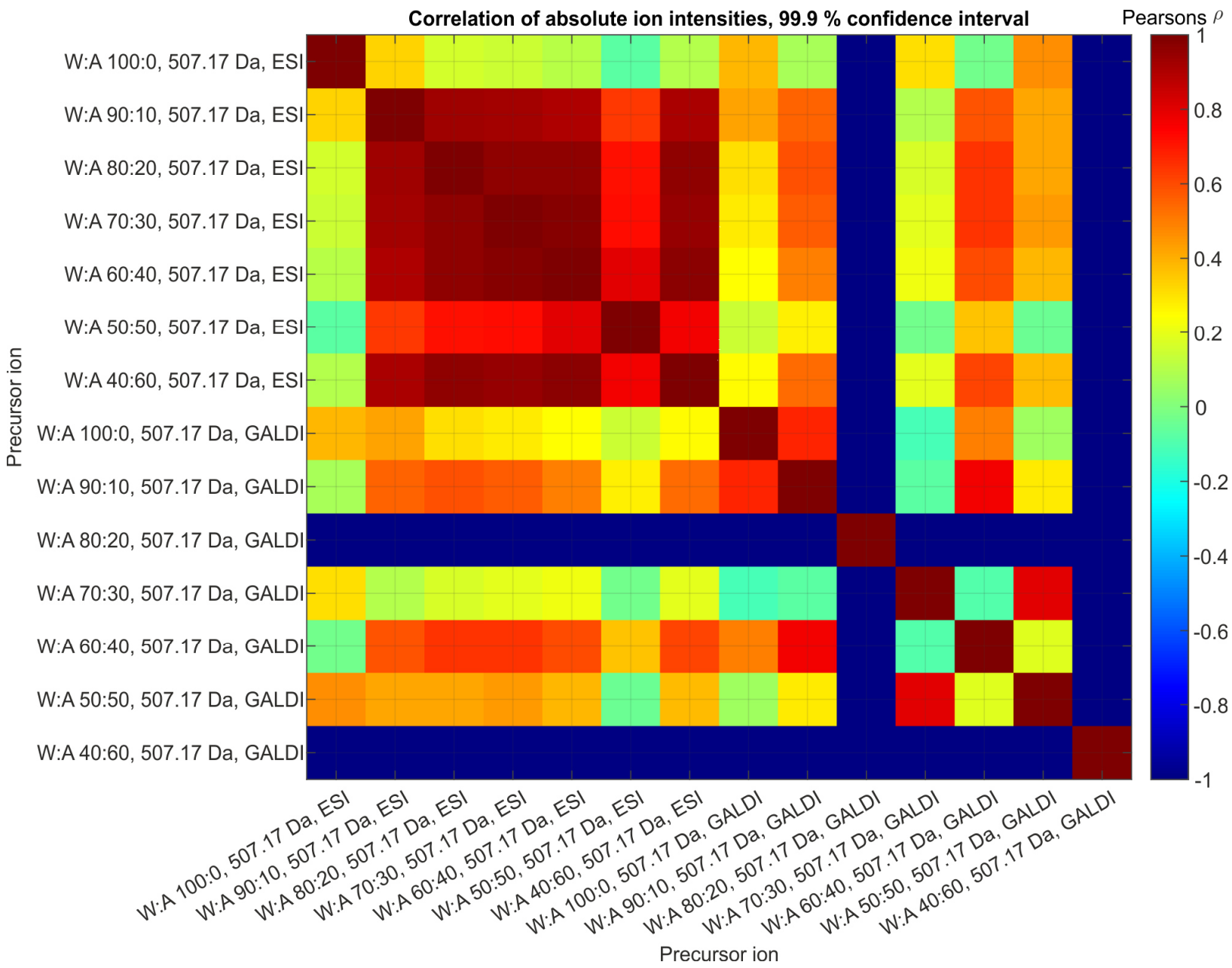

Figure S20: Correlation heatmap of the ESI-MS/MS and GALDI-MS/MS analyses of precursor ion 507.17 Da ($C_{28}H_{28}O_9$, $[M-H]^-$) for all analyzed spruce kraft lignin fractions. For this chemometric evaluation the absolute intensity information and a confidence interval of 99.9 % were used.

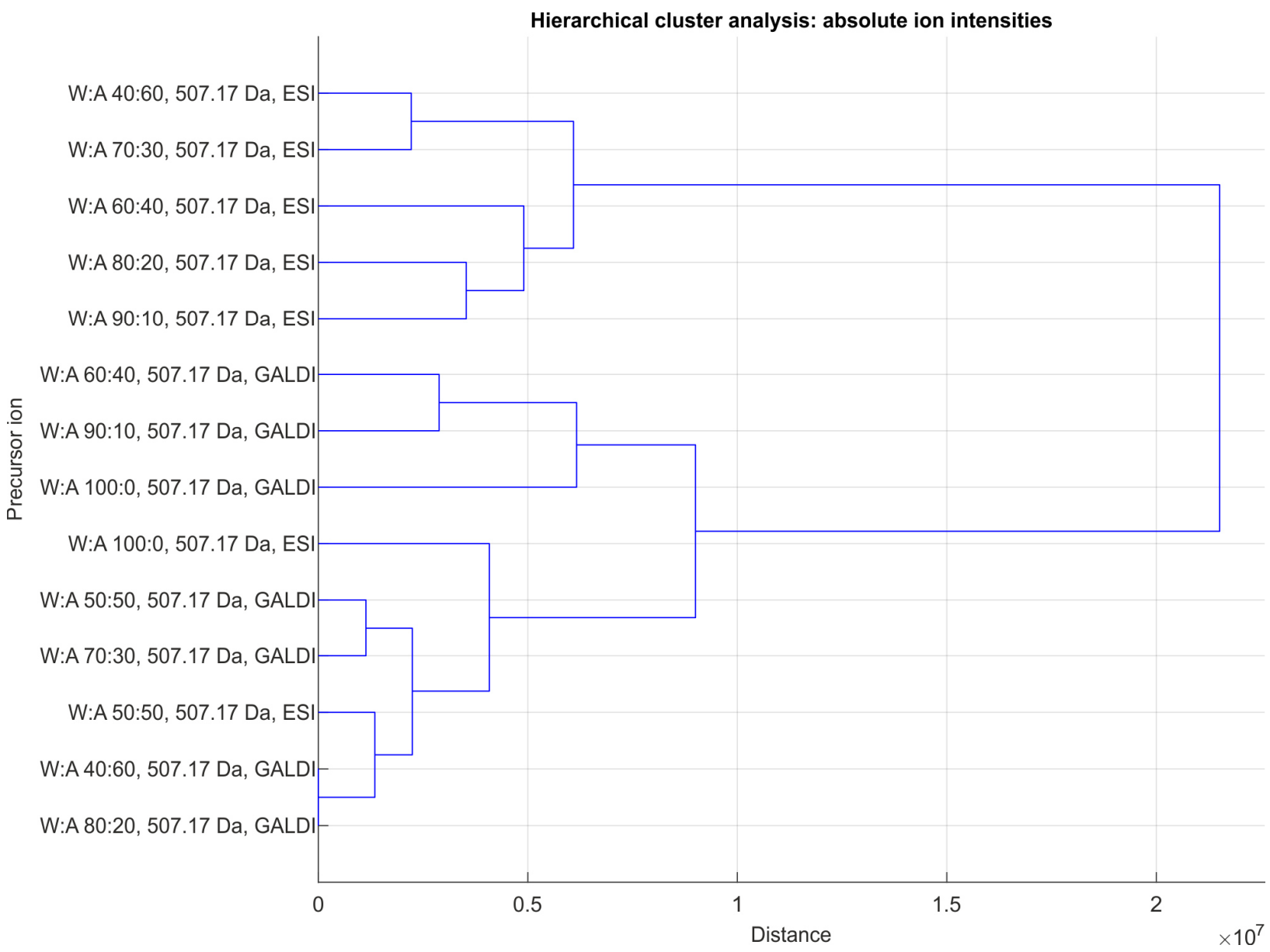

Figure S21: Dendrogram of the hierarchical cluster analysis of the ESI- and GALDI-MS/MS data sets of the seven lignin fractions for precursor ion 507.17 Da ($C_{28}H_{28}O_9$, $[M-H]^-$), using the relative intensity information of selected fragment ions. For the cluster analysis, Euclidean distance metric and Ward linkage for distance computing were applied.

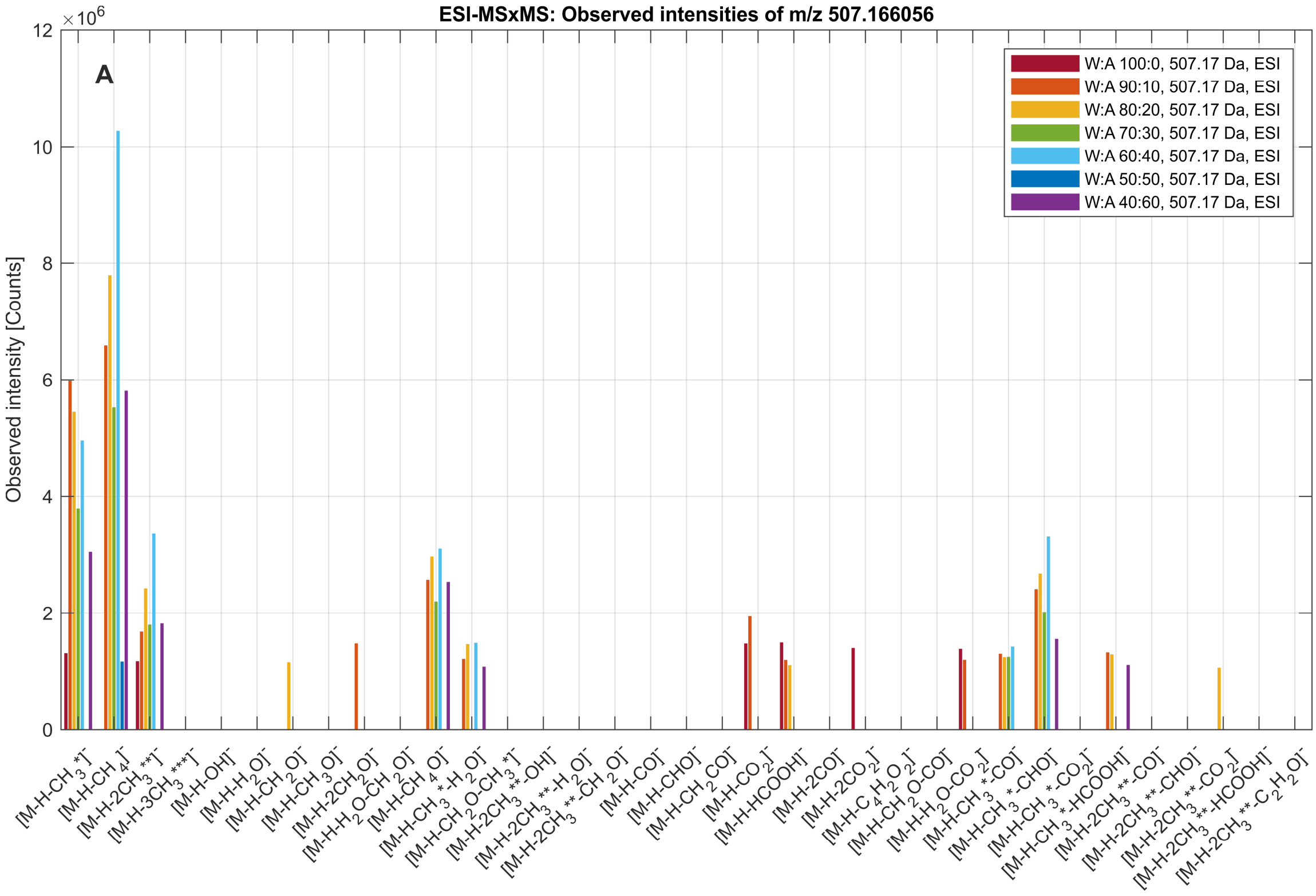

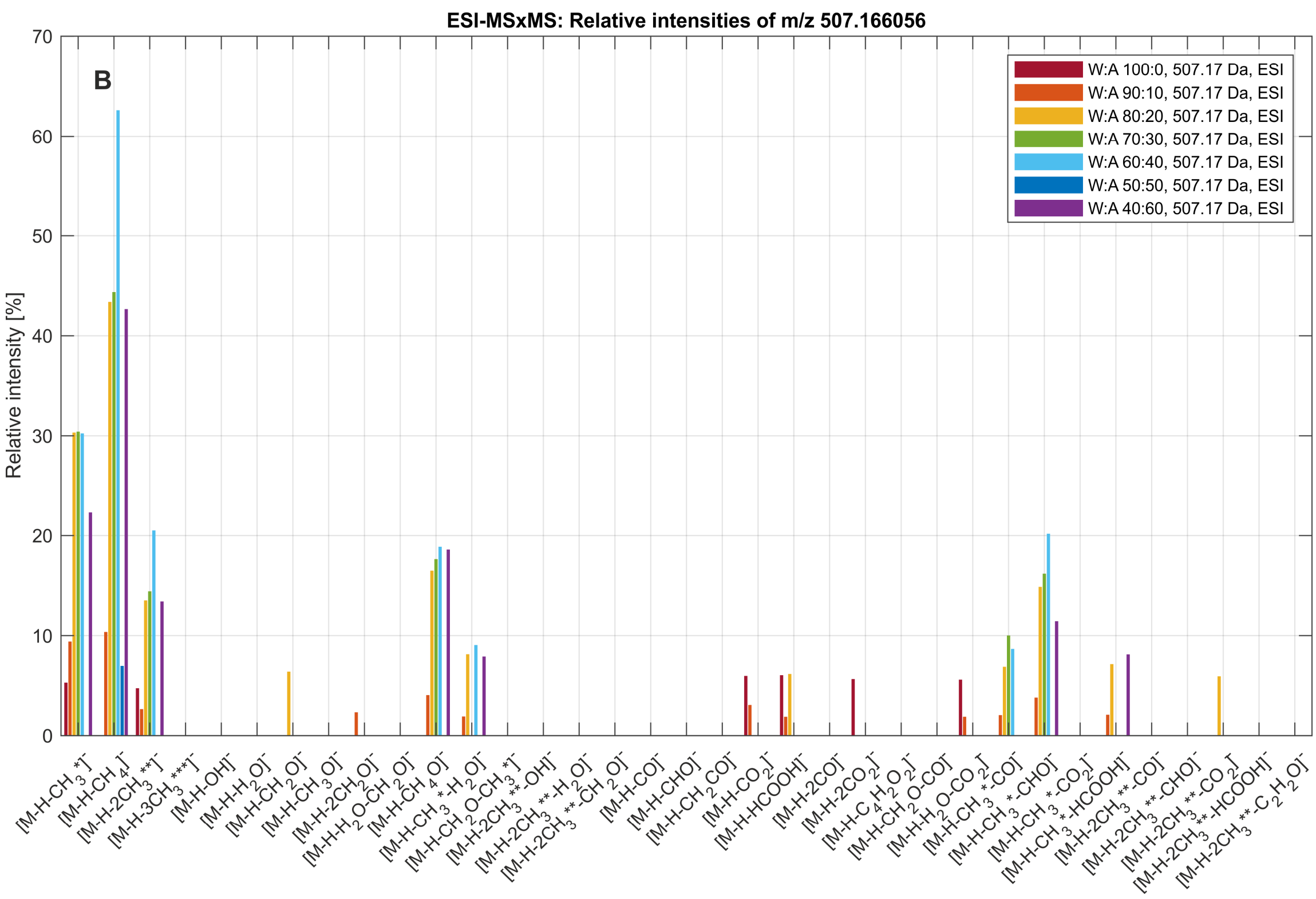

Figure S22: Comparison of the absolute (A) and relative (B) ion intensities of the 35 lignin characteristic product ions for precursor ion 507.17 Da ($C_{28}H_{28}O_9$, $[M-H]^-$), as detected by ESI-FT-ICR-MS/MS.

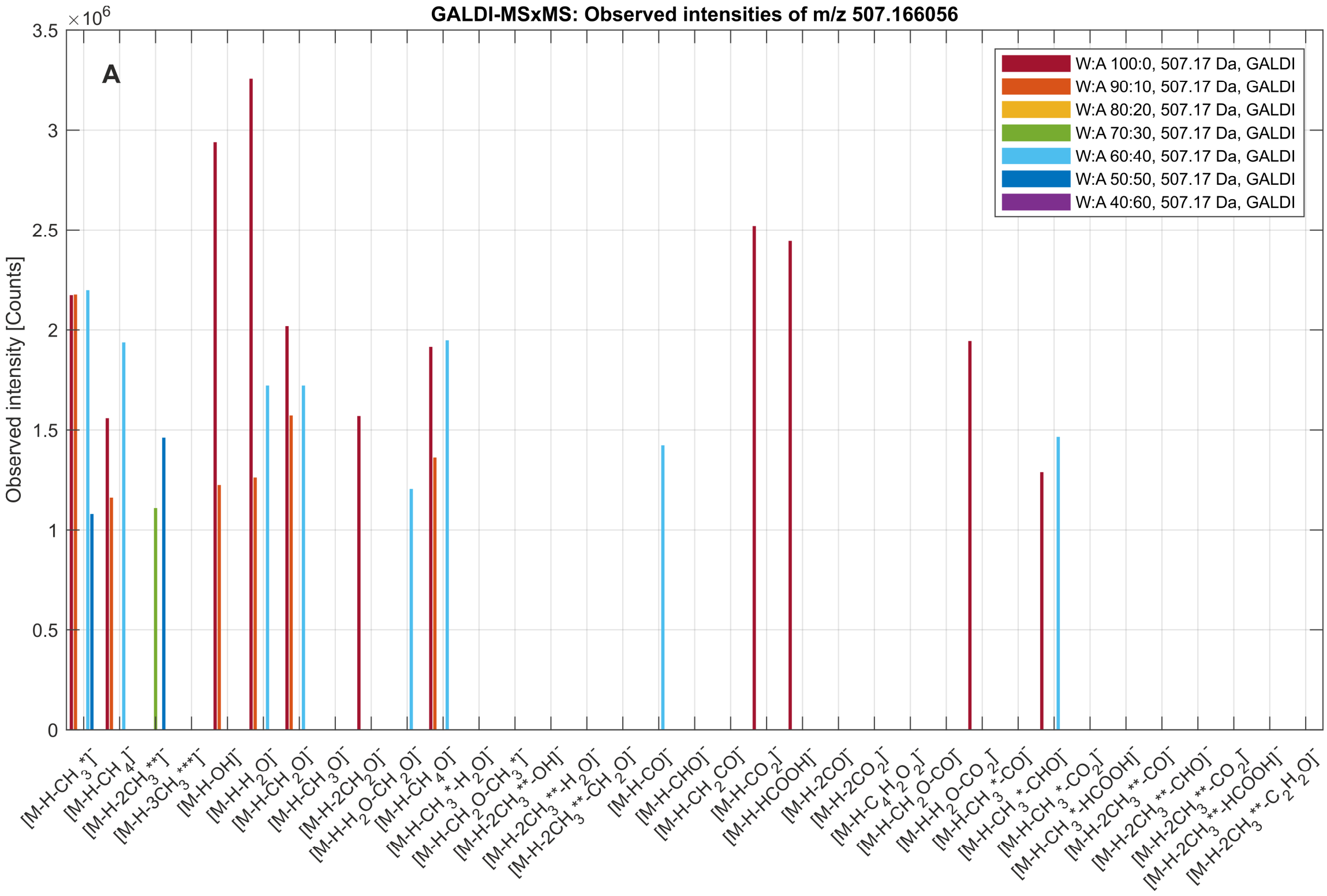

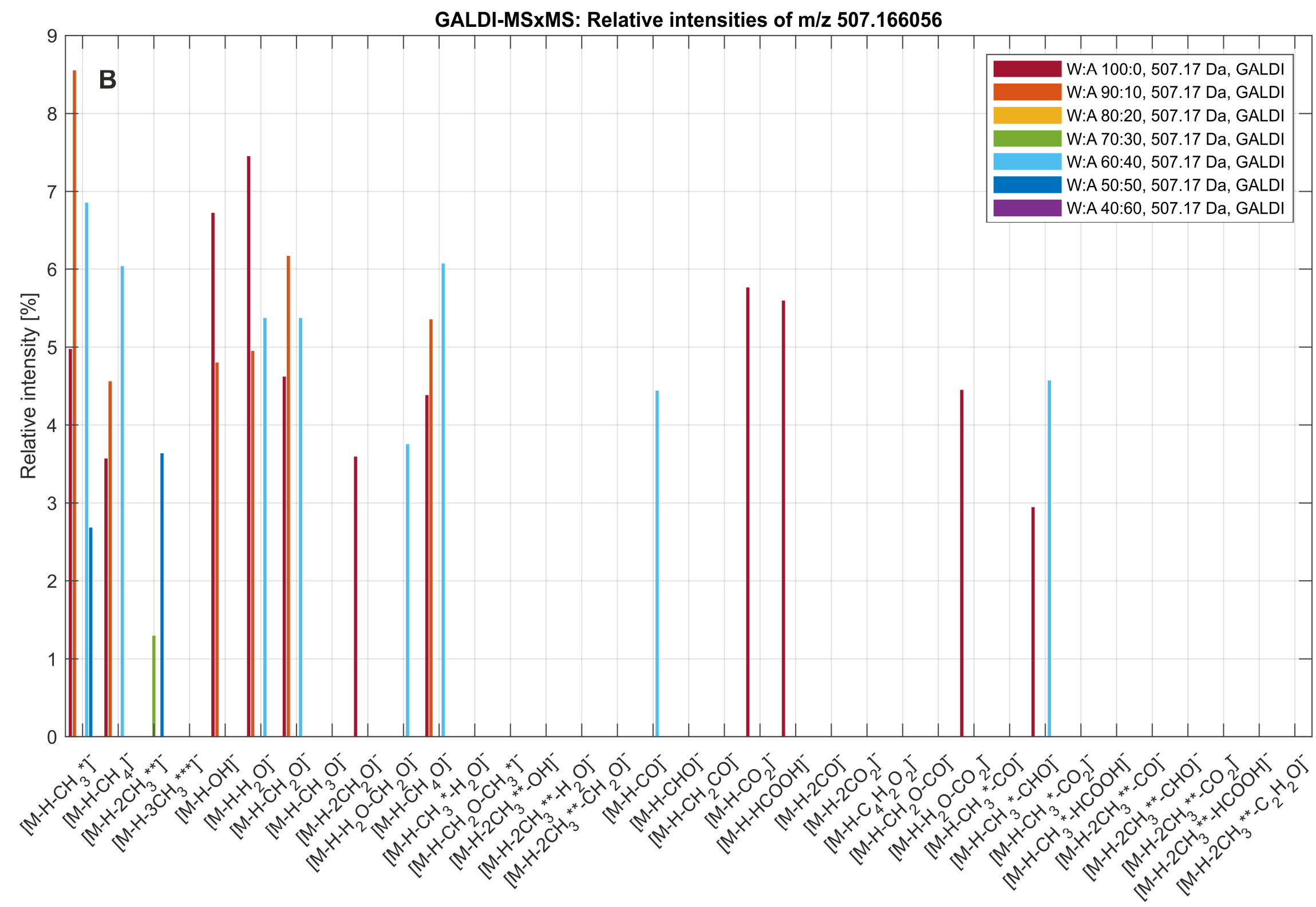

Figure S23: Comparison of the absolute (A) and relative (B) ion intensities of the 35 lignin characteristic product ions for precursor ion 507.17 Da ($C_{28}H_{28}O_9$, $[M-H]^-$), as detected by GALDI-FT-ICR-MS/MS.

A

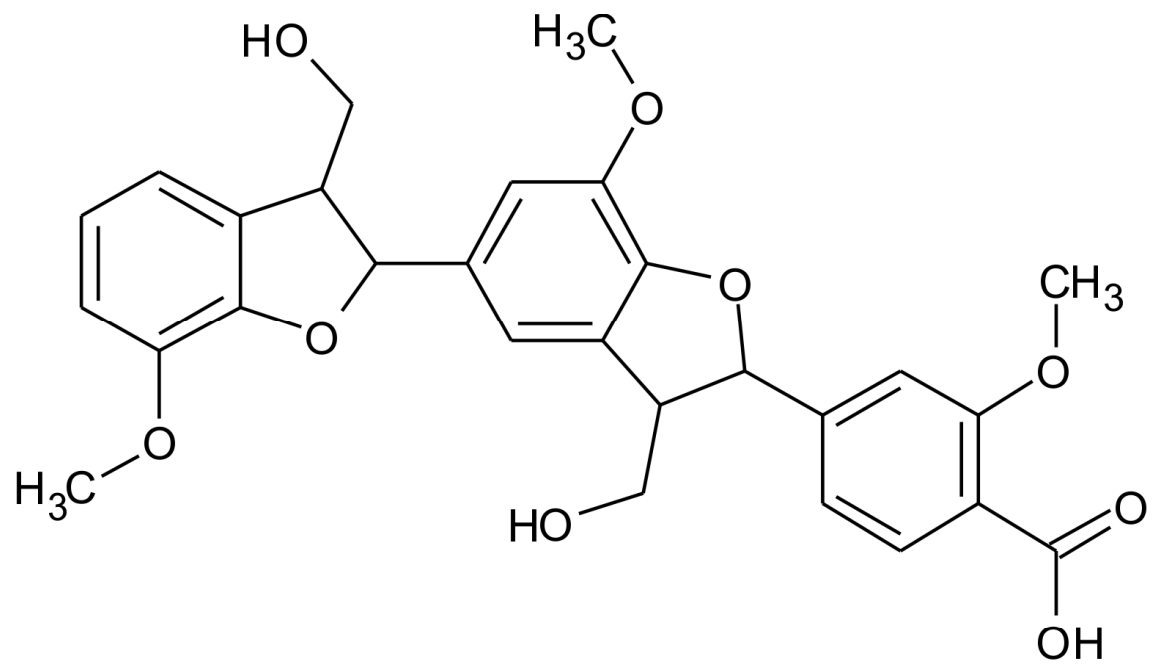

B

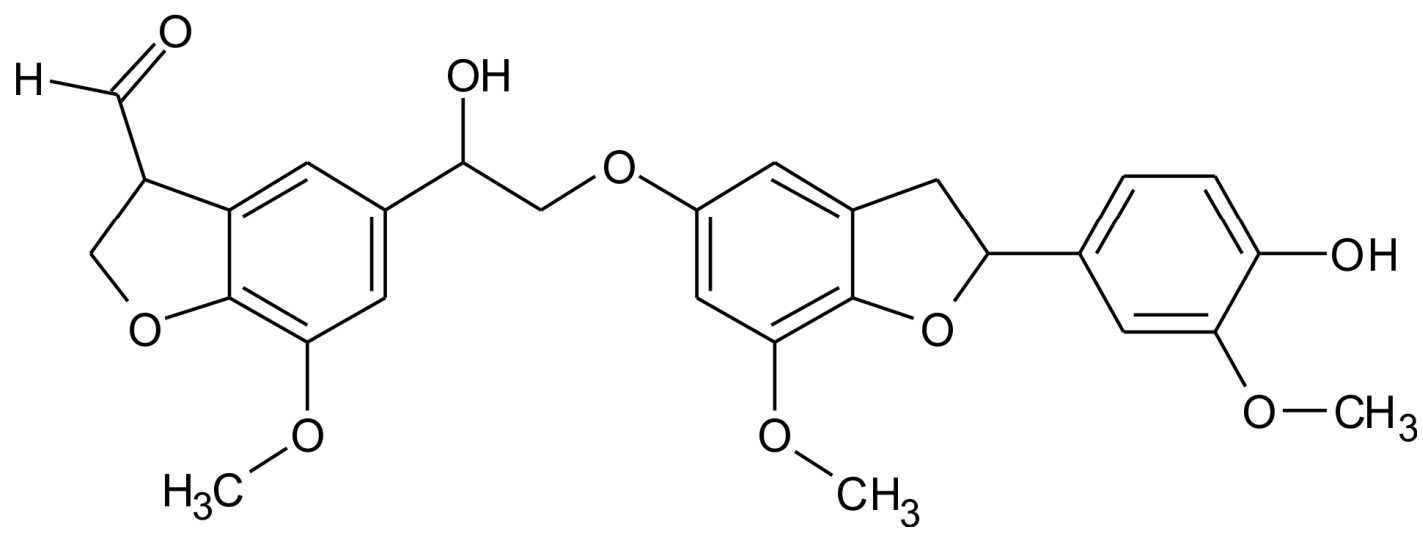

Figure S24: Proposed structures for a molecule with the molecular formula $C_{28}H_{28}O_9$, which was detected by ESI- and GALDI-FT-ICR-MS in all lignin fractions as $[M-H]^-$ ion and structurally studied using MS/MS. According to the MS/MS results, structure A is mainly supposable for the fractions W:A 100:0 – 80:20 (v/v), whereas structure B is more likely for the spruce kraft lignin fractions, generated with higher acetone amounts (W:A 70:30 – 40:60 (v/v)). Note that assigned molecular formulae might correspond to multiple structural isomers.

S16. Hetero spectroscopic correlation of ^{13}C-NMR and MS/MS data sets for a specific precursor ion

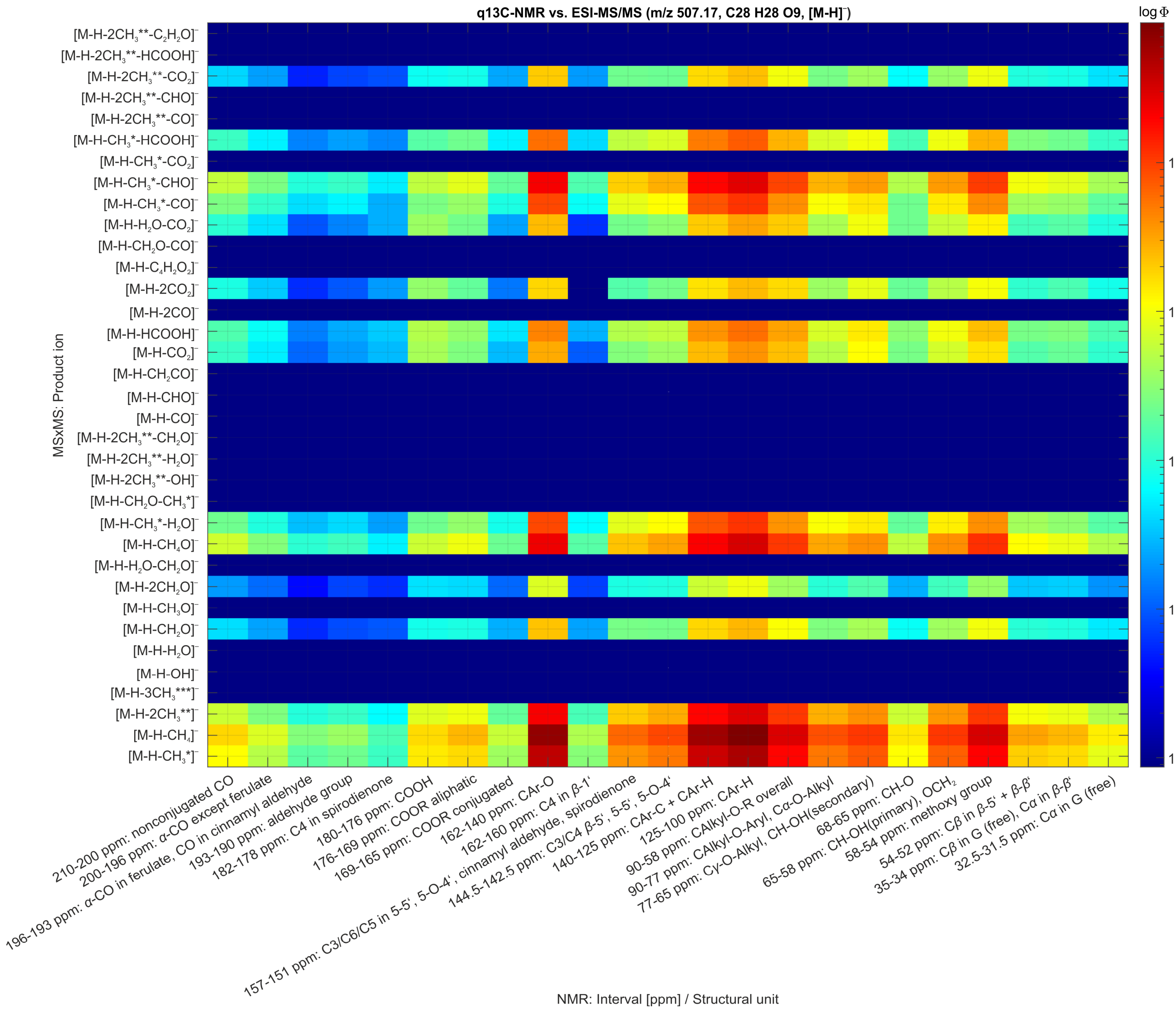

Figure S25: Correlation heatmap as a result of the hetero spectral combination of quantitative ^{13}C-NMR segments and ESI-MS/MS data sets for one selected precursor ion in all analyzed fractions (507.17 Da, $C_{28}H_{28}O_9$, $[M-H]^-$). The calculated correlation coefficients[17] Φ are presented logarithmic and color-coded.

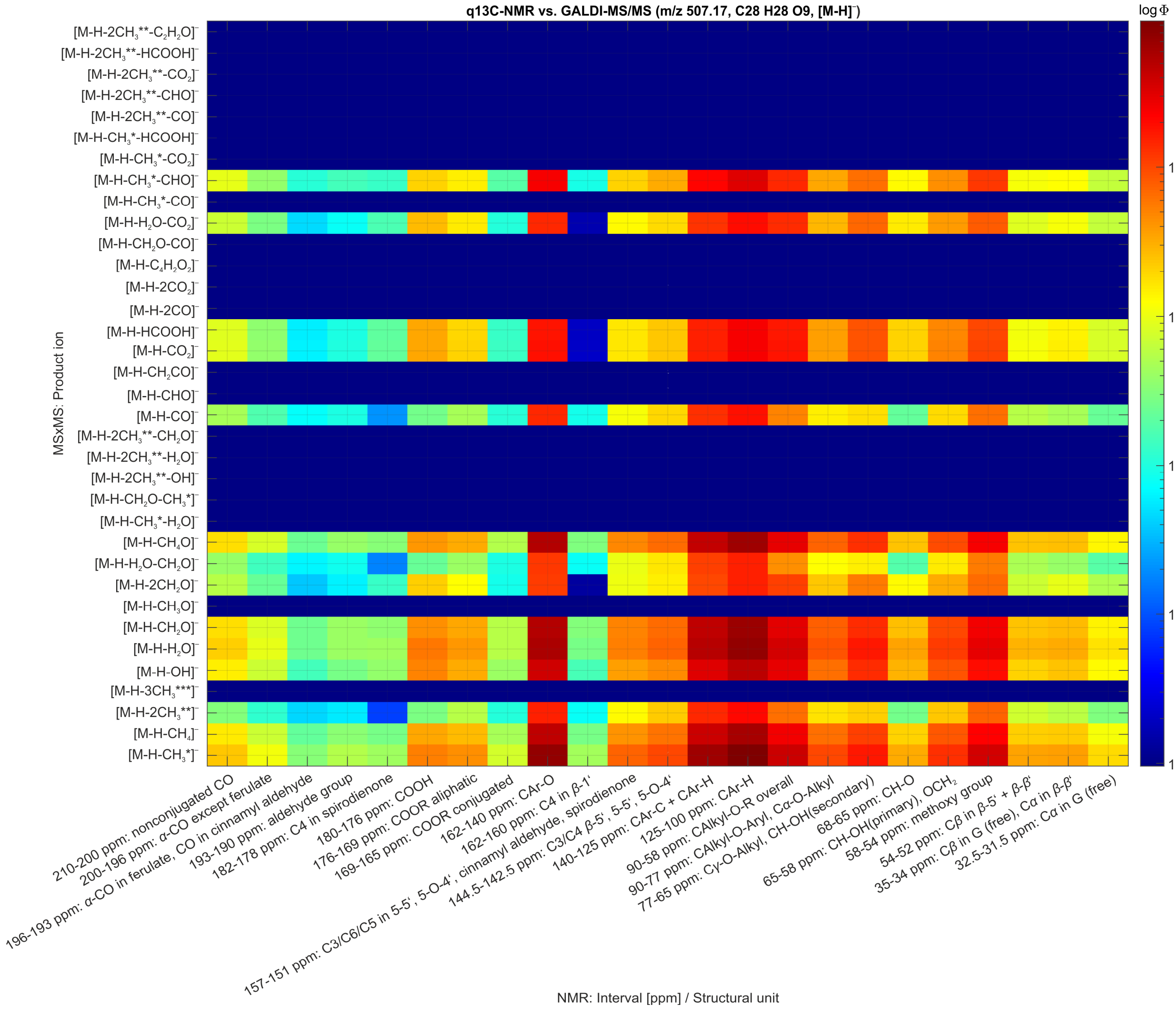

Figure S26: Correlation heatmap as a result of the hetero spectral combination of quantitative ^{13}C-NMR segments and GALDI-MS/MS data sets for one selected precursor ion in all analyzed fractions (507.17 Da, $C_{28}H_{28}O_9$, $[M-H]^-$). The calculated correlation coefficients[17] Φ are presented logarithmic and color-coded.

S17. Hetero spectroscopic correlation of FT-IR and MS/MS data sets

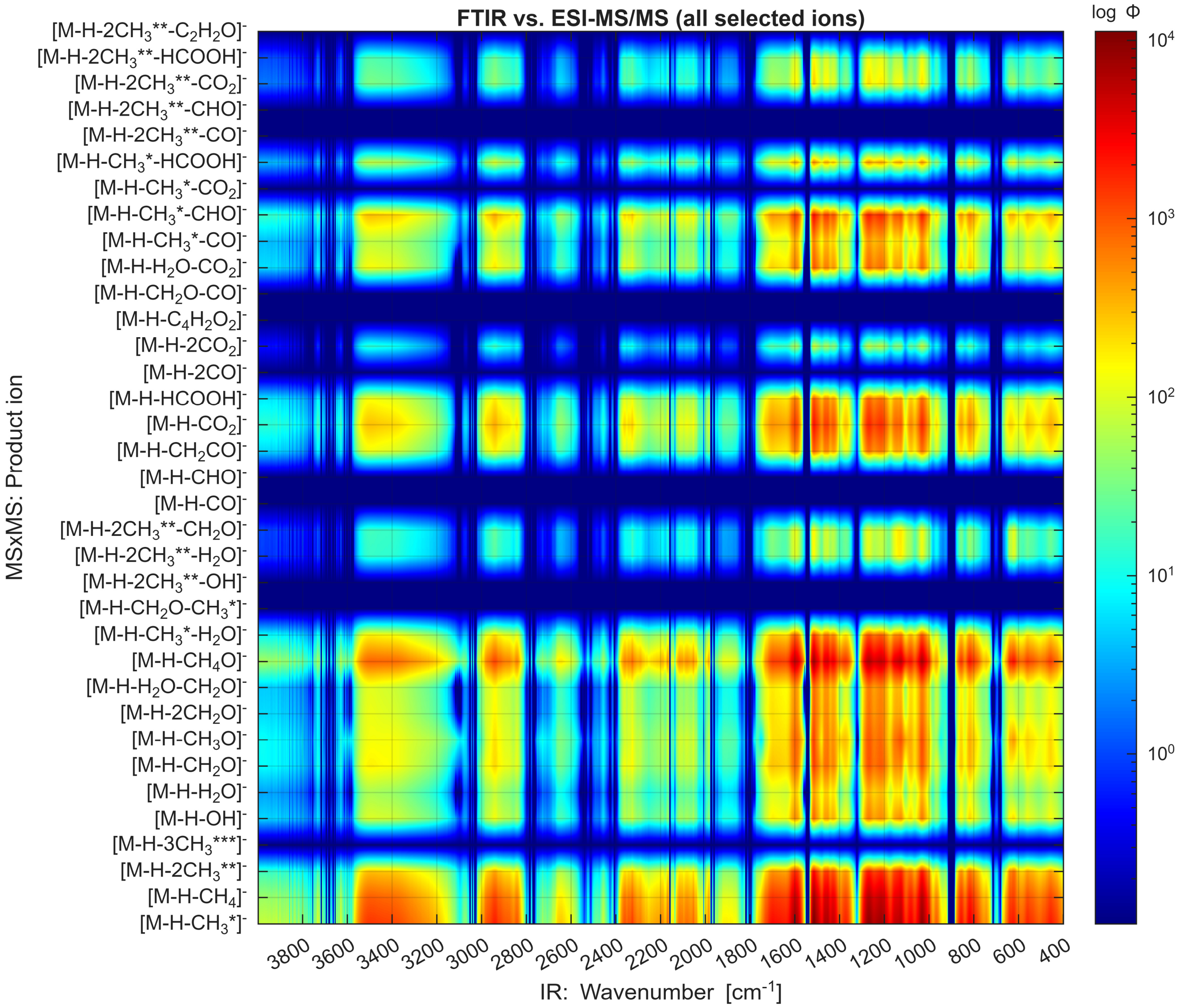

Figure S27: Correlation heatmap as a result of the hetero spectral combination of FT-IR and ESI-MS/MS data sets for all isolated precursor ions (42). The calculated correlation coefficients[17] Φ are presented logarithmic and color-coded.

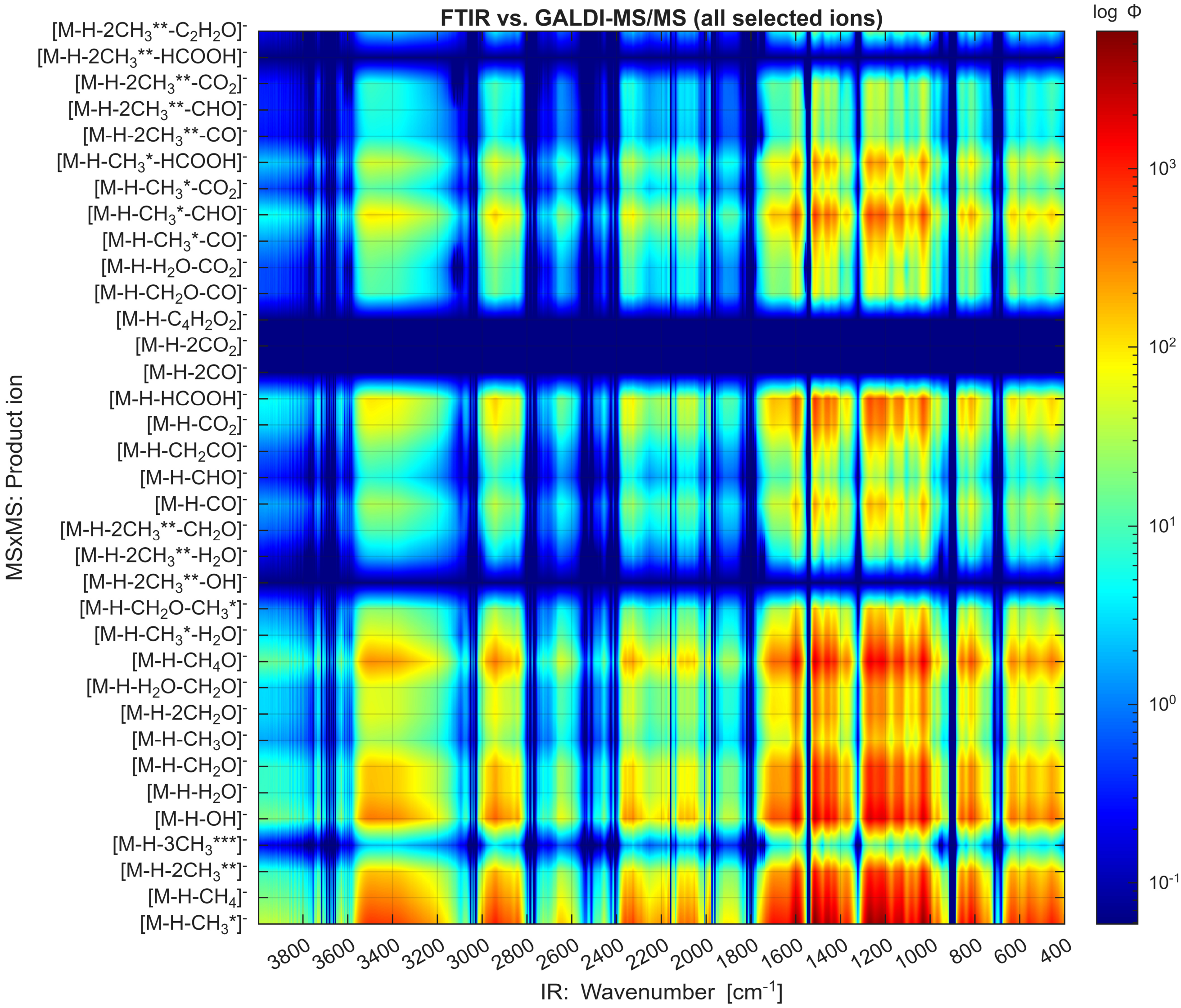

Figure S28: Correlation heatmap as a result of the hetero spectral combination of FT-IR and GALDI-MS/MS data sets for all isolated precursor ions (42). The calculated correlation coefficients[17] Φ are presented logarithmic and color-coded.

S18. Hetero spectroscopic correlation of FT-IR and MS/MS data sets for a specific precursor ion

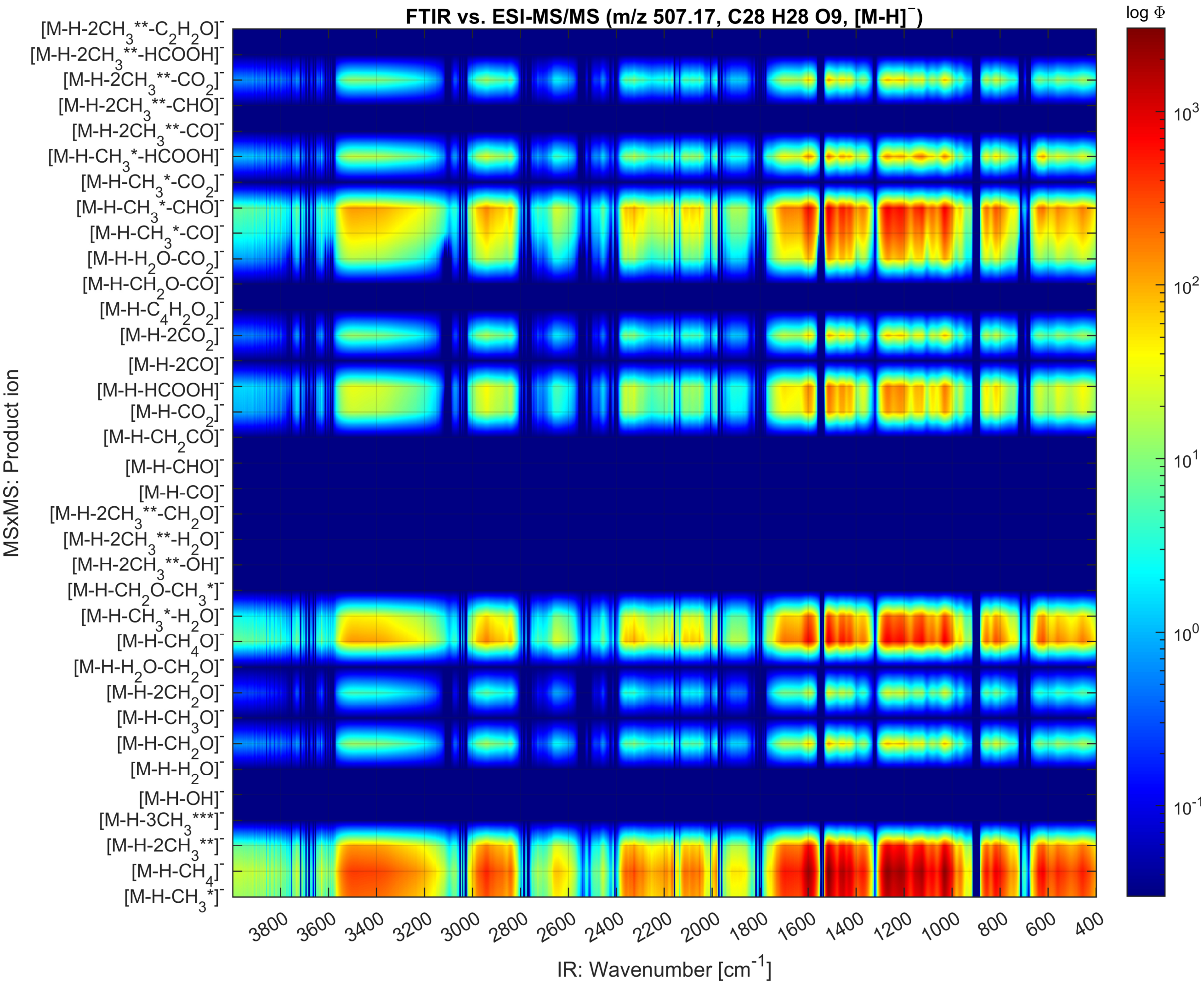

Figure S29: Correlation heatmap as a result of the hetero spectral combination of FT-IR and ESI-MS/MS data sets for one selected precursor ion in all analyzed fractions (507.17 Da, $C_{28}H_{28}O_9$, $[M-H]^-$). The calculated correlation coefficients[17] Φ are presented logarithmic and color-coded.

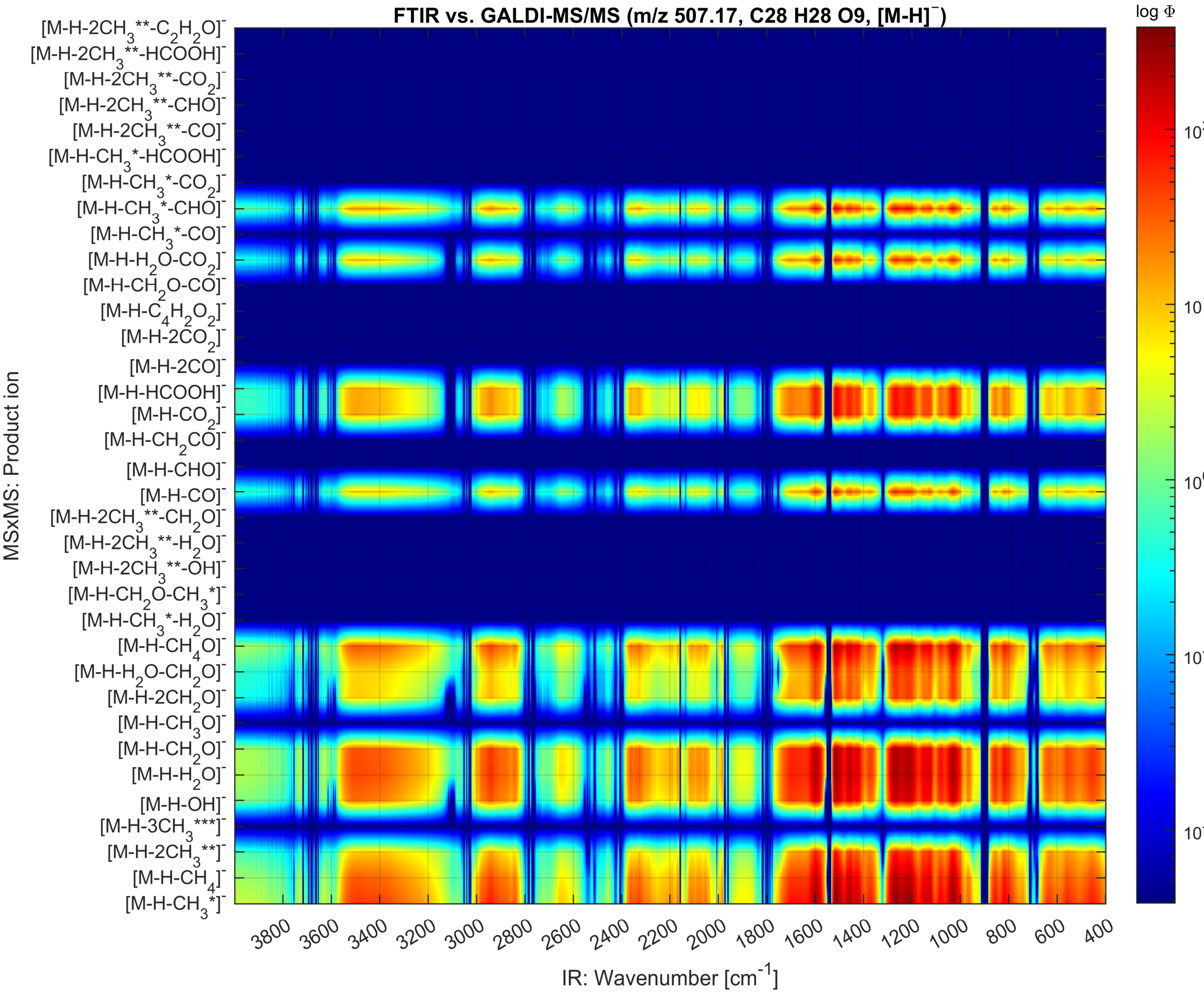

Figure S30: Correlation heatmap as a result of the hetero spectral combination of FT-IR and GALDI-MS/MS data sets for one selected precursor ion in all analyzed fractions (507.17 Da, $C_{28}H_{28}O_9$, $[M-H]^-$). The calculated correlation coefficients[17] Φ are presented logarithmic and color-coded.

References:

[1] Schwanninger, M.; Rodrigues, J. C.; Pereira, H.; Hinterstoisser, B. Effects of short-time vibratory ball milling on the shape of FT-IR spectra of wood and cellulose. *Vib. Spectrosc.* **2004**, *36* (1), 23-40. DOI: 10.1016/j.vibspec.2004.02.003.
[2] Sun, X.-F.; Sun, R.; Fowler, P.; Baird, M. S. Extraction and characterization of original lignin and hemicelluloses from wheat straw. *J. Agric. Food Chem.* **2005**, *53* (4), 860-870. DOI: 10.1021/jf040456q.
[3] Xu, F.; Yu, J.; Tesso, T.; Dowell, F.; Wang, D. Qualitative and quantitative analysis of lignocellulosic biomass using infrared techniques: A mini-review. *Appl. Energy* **2013**, *104*, 801-809. DOI: 10.1016/j.apenergy.2012.12.019.
[4] Socrates, G. *Infrared and Raman characteristic group frequencies: Tables and charts*, 3. ed.; Wiley, **2001**.
[5] Kačuráková, M.; Wellner, N.; Ebringerová, A.; Hromádková, Z.; Wilson, R.; Belton, P. Characterisation of xylan-type polysaccharides and associated cell wall components by FT-IR and FT-Raman spectroscopies. *Food Hydrocolloids* **1999**, *13* (1), 35-41. DOI: 10.1016/S0268-005X(98)00067-8.
[6] Stark, N. M.; Yelle, D. J.; Agarwal, U. P., Eds. *Techniques for Characterizing Lignin*; Elsevier, **2016**. DOI: 10.1016/B978-0-323-35565-0.00004-7.
[7] Boeriu, C. G.; Bravo, D.; Gosselink, R. J.; van Dam, J. E. Characterisation of structure-dependent functional properties of lignin with infrared spectroscopy. *Ind. Crops Prod.* **2004**, *20* (2), 205-218. DOI: 10.1016/j.indcrop.2004.04.022.
[8] Hatakeyama, H.; Nagasaki, C.; Yurugi, T. Relation of certain infrared bands to conformational changes of cellulose and cellulose oligosaccharides. *Carbohydr. Res.* **1976**, *48* (2), 149–158. DOI: 10.1016/S0008-6215(00)83211-5.
[9] Capanema, E. A.; Balakshin, M. Y.; Kadla, J. F. Quantitative characterization of a hardwood milled wood lignin by nuclear magnetic resonance spectroscopy. *J. Agric. Food Chem.* **2005**, *53* (25), 9639–9649. DOI: 10.1021/jf0515330.
[10] Wen, J.-L.; Sun, S.-L.; Xue, B.-L.; Sun, R.-C. Recent Advances in Characterization of Lignin Polymer by Solution-State Nuclear Magnetic Resonance (NMR) Methodology. *Materials* **2013**, *6* (1), 359–391. DOI: 10.3390/ma6010359.
[11] Del Río, J. C.; Rencoret, J.; Prinsen, P.; Martínez, Á. T.; Ralph, J.; Gutiérrez, A. Structural characterization of wheat straw lignin as revealed by analytical pyrolysis, 2D-NMR, and reductive cleavage methods. *J. Agric. Food Chem.* **2012**, *60* (23), 5922–5935. DOI: 10.1021/jf301002n.
[12] Capanema, E. A.; Balakshin, M. Y.; Kadla, J. F. A comprehensive approach for quantitative lignin characterization by NMR spectroscopy. *J. Agric. Food Chem.* **2004**, *52* (7), 1850–1860. DOI: 10.1021/jf035282b.
[13] Kringstad, K. P.; Mörck, R. 13 C-NMR Spectra of Kraft Lignins. *Holzforschung* **1983**, *37* (5), 237–244. DOI: 10.1515/hfsg.1983.37.5.237.
[14] Calvo-Flores, F. G., Ed. *Lignin and lignans as renewable raw materials: Chemistry, technology and applications*; Wiley series in renewable resources; Wiley, 2015. DOI: 10.1002/9781118682784.

[15] Nong, J. P. *Einfluss der Ligninstruktur auf die Sorptionseigenschaften von Ligninhydrogelen*. Dissertation, Technische Universität Dresden, Dresden, 2024. https://nbn-resolving.org/urn:nbn:de:bsz:14-qucosa2-906559.

[16] Ayala-Ortiz, C.; Graf-Grachet, N.; Freire-Zapata, V.; Fudyma, J.; Hildebrand, G.; AminiTabrizi, R.; Howard-Varona, C.; Corilo, Y. E.; Hess, N.; Duhaime, M. B.; Sullivan, M. B.; Tfaily, M. M. MetaboDirect: an analytical pipeline for the processing of FT-ICR MS-based metabolomic data. *Microbiome* **2023**, *11* (1), 28. DOI: 10.1186/s40168-023-01476-3.

[17] Noda, I. Recent developments in two-dimensional (2D) correlation spectroscopy. *Chin. Chem. Lett.* **2015**, *26* (2), 167-172. DOI: 10.1016/j.cclet.2014.10.006.
